# Structure-based macrocyclization of α-ketoamides leads to potent inhibitors of coronaviral and enteroviral proteases

**DOI:** 10.1038/s42004-026-02151-y

**Published:** 2026-08-06

**Authors:** Ravi Kumar Akula, Haifa El Kilani, Alina Metzen, Sanjata Joshi, Hilal Durmaz, Robin Veenstra, Judith Röske, Daniel L. Hurdiss, Frank J. M. Van Kuppeveld, Katharina Rox, Rolf Hilgenfeld, Mark Brönstrup

**Affiliations:** 1 Department of Chemical Biology, Helmholtz Centre for Infection Research, Braunschweig, Germany; 2 Institute of Molecular Medicine, University of Lübeck, Lübeck, Germany; 3 German Center for Infection Research (DZIF), Hamburg-Lübeck-Borstel-Riems Site, Lübeck, Germany; 4 German Center for Infection Research (DZIF), Hannover-Braunschweig Site, Braunschweig, Germany; 5 Research Group Pharmacokinetics/Pharmacodynamics Unit, Helmholtz Centre for Infection Research, Braunschweig, Germany; 6 Virology section, Division of Infectious Diseases & Immunology, Department of Biomolecular Health Sciences, Faculty of Veterinary Medicine, Utrecht University, Utrecht, The Netherlands; 7 Institute of Organic Chemistry and Biomolecular Drug Research Centre (BMWZ), Leibniz University Hannover, Hannover, Germany

**Keywords:** Structure-based drug design, X-ray crystallography

## Abstract

Viral proteases represent validated targets for direct-acting antivirals and the treatment of associated infections. In co-crystal structures of M^pro^ of SARS-CoV-2 with peptidomimetic inhibitors, we noticed a spatial proximity of sidechains filling the S1’ and S2 pockets, as well as those filling S3 and S1 pockets. To enhance molecular rigidity, the proximal residues were conformationally fixed by macrocyclization. We report the synthesis of two macrocyclic series, i.e. exocyclic nitriles with linked P3 and P1 residues and endocyclic α-ketoamides with linked P1’ and P2 residues, and characterize their binding modes and bioactivities. The 17-membered macrocyclic α-ketoamide **20 f** inhibited M^pro^ (IC₅₀ = 370 nM) and exerted anti-SARS-CoV-2 effects (EC₅₀ = 1.9 μM). Leveraging structural similarities between M^pro^ and the 3C^pro^ of enterovirus D68, we describe with two co-crystal structures how α-ketoamide macrocycles bound to and inhibited the enteroviral protease. Notably, **20 f** exhibited very potent antiviral activities with EC₅₀‘s of 33, 133, and 146 nM against EV-D68, EV-A71, and CVB3, respectively. The study demonstrates how broad-spectrum activity can be achieved with direct-acting antivirals.

## Abstract (web summary)

Viral proteases are key targets for developing direct-acting antivirals to combat infections like SARS-CoV-2. Here, the authors synthesize macrocyclic α-ketoamide inhibitors, demonstrating potent inhibition of SARS-CoV-2 and enteroviruses, highlighting macrocyclization’s potential to enhance antiviral efficacy and achieve broad-spectrum activity.

## Introduction

**Fig. 1:**
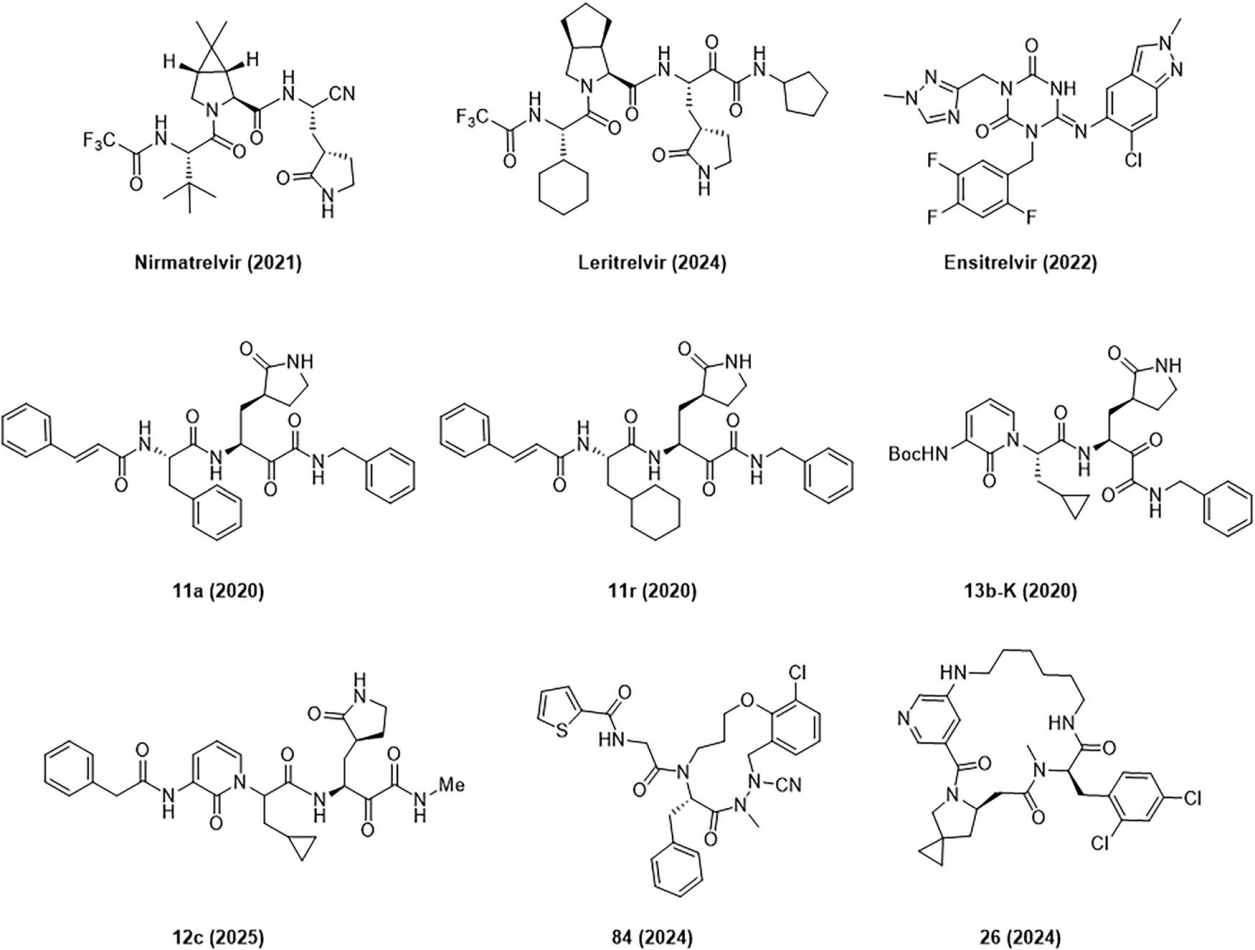
Structures of selected previously reported SARS-CoV-2 M^pro^ inhibitors. The covalent binders nirmatrelvir and leritrelvir and the non-covalent binder ensitrelvir have been licensed for COVID-19. The linear compounds **13b-K,** **12c,** **11a**, and **11r** were starting points of our work. Compounds **84** and **26** represent the first macrocyclic M^pro^ inhibitors. The year of first publication is given in brackets.

Severe Acute Respiratory Syndrome Coronavirus 2 (SARS-CoV-2), the etiological agent of the COVID-19 pandemic, critically depends on its main protease (M^pro^), also referred to as 3C-like protease (3 CL^pro^), for viral replication and maturation^1,2^. M^pro^ facilitates the proteolytic cleavage of the viral polyprotein at conserved recognition sites, generating functional proteins essential for the assembly and propagation of the virus^3^. Given its pivotal role in the viral life cycle and the absence of homologous proteases in humans, M^pro^ constitutes an attractive and selective target for the development of antiviral therapeutics. Significant efforts to inhibit M^pro^ have led to the discovery and development of numerous potent compounds that bind either covalently or non-covalently to the protease^2,4,5^. Covalent inhibitors exploit the highly nucleophilic catalytic cysteine residue (Cys145) within the active site of M^pro^, forming either reversible or irreversible covalent bonds that effectively block enzymatic activity. This binding mode led to approved drugs such as nirmatrelvir^6^, leritrelvir^7^, simotrelvir^8^, and other potent compounds in clinical trials^9–12^, notably these compounds either employ a nitrile or an α-ketoamide as a reactive warhead (Fig. 1). Also, an inhibition through non-covalent interactions is possible, as shown by the approved drug ensitrelvir^13^ and a series of other potent compounds^14–18^. Despite these advances, the search for improved M^pro^ inhibitors continues to enhance their selectivity, pharmacokinetic and safety profiles. The use of nirmatrelvir and the co-administered drug ritonavir is complicated due to drug-drug interactions and might be impaired due to resistant variants featuring the E166V and E166A mutation^19,20^. Recently, it has been reported that resistance to one M^pro^ inhibitor might increase susceptibility to another, suggesting that finding additional inhibitors is valuable^21^. By leveraging insights from existing compounds^7,9,14,22–23^ and expanding towards new chemical formats^24^, the pursuit of effective M^pro^ inhibitors remains a cornerstone of therapeutics against emerging SARS-CoV-2 variants and for a preparedness against future pandemic threats by coronaviruses.

Moreover, there are opportunities to expand such protease inhibitors to broader spectrum antivirals, because the 3C-like protease M^pro^ of coronaviruses shares structural similarities to the 3C proteases of enteroviruses (EV), including the 3C of the Coxsackievirus B3 (CVB3). Finding broad-spectrum inhibitors is non-obvious, because of pronounced differences in size, domain structure, S2 pocket specificity, or catalytic residues between the proteases^25^. But yet, we previously reported the peptidomimetics **11a** and **11r** as antiviral agents with activity against both coronaviruses and enteroviruses^26^, motivating us to investigate possible line extensions for such protease inhibitors^26,27^.

Macrocyclic drugs, first discovered from natural sources, have been recognized as essential medicines, e.g., due to their immunosuppressant, antibiotic or antitumor activities^28–31^. Their increased structural rigidity and diverse functionality within a pre-organized ring structure minimizes entropy loss upon binding, potentially enhancing target affinity. Furthermore, macrocyclization has been demonstrated to confer resistance to proteolytic degradation, facilitate intramolecular hydrogen bonding, and improve bioavailability, thus enabling compounds to effectively reach intracellular targets. These advantages have led to an intense exploitation of macrocycles beyond natural products by designing synthetic scaffolds in the past two decades^28,32,33^. Because macrocyclic peptides typically exhibit lower off-target interactions, higher membrane permeability, and improved proteolytic stability compared to linear peptides. A first successful application of the concept to antivirals has been demonstrated by ciluprevir, an inhibitor of the NS3/4A protease of hepatitis C virus (HCV) that was derived from a linear substrate analog. Further optimization led to the approved anti-HCV drugs simeprevir and grazoprevir^34^. Also, macrocycles against 3C proteases of hRV^35^ and enteroviruses have been reported^36^. Recently, the groups of Gütschow, Müller and Sofia found macrocyclic compounds inhibiting M^pro^ of SARS-CoV-2, i.e., the covalently binding azapeptide nitrile **84** and the noncovalently binding peptidomimetic **26**, respectively (Fig. 1)^37,38^. Our groups have investigated α-ketoamides as reversible, covalent binders of 3C proteases such as M^pro^ using structural biology, and reported the ketoamide **13b** as one of the first M^pro^ inhibitors against SARS-CoV-2 in early 2020. Building on insights from co-crystal structures of related ketoamides^26,39,40^, such as **13b-K** or **11a** (Fig. 1), we designed two series of covalently binding, macrocyclized compounds. We prepared four nitriles with linked P3 and P1 residues and eight α-ketoamides with linked P1’ and P2 residues, and studied their binding modes by co-crystal structures, as well as their structure-activity relationships (SAR). Finally, we investigated their ability to inhibit enteroviral 3C proteases and found a second indication for the macrocycles as antivirals against enteroviruses. A first profiling of ADME properties suggests potential for further preclinical development.

## Results

### Design rationale

**Fig. 2:**
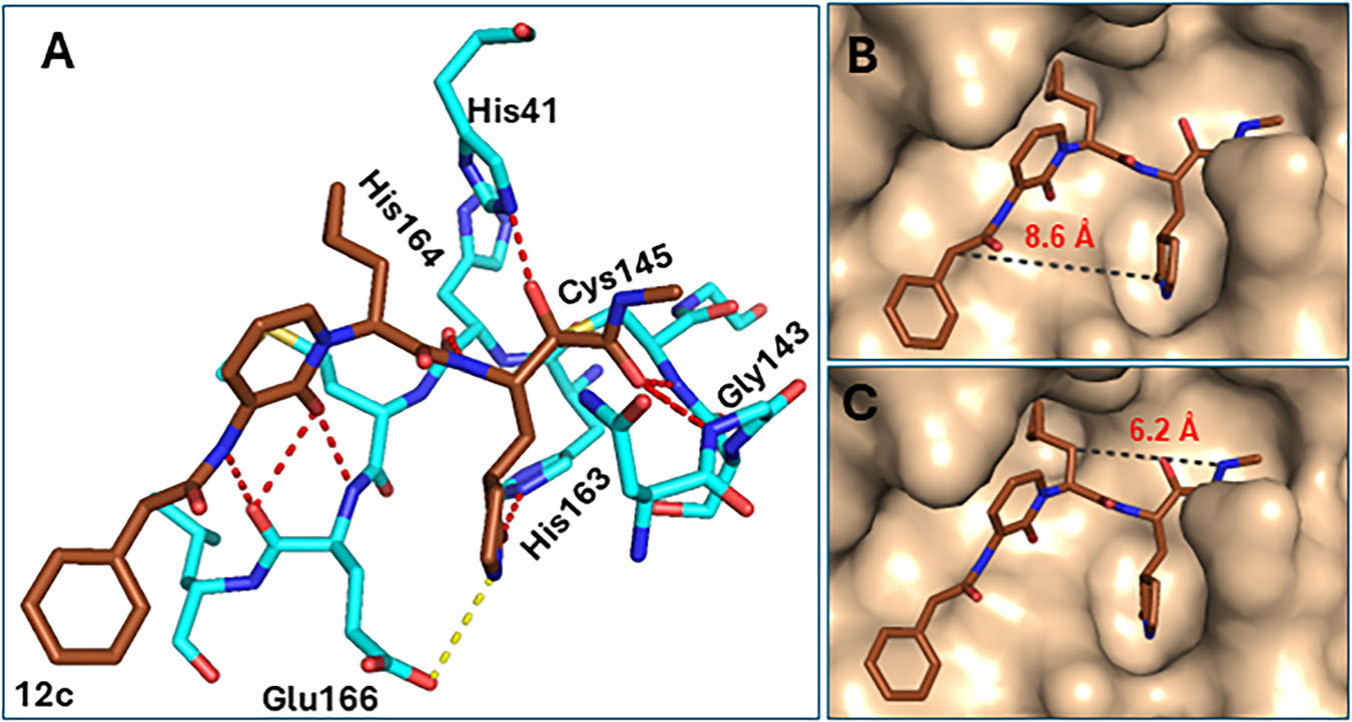
Co-crystal structure of M^pro^ in complex with 12c (PDB: 8AIZ). **A** Compound **12c** is colored in brown, and M^pro^ residues are colored in cyan. H-bonds between **12c** and the corresponding M^pro^ residues are colored in red; yellow dashes highlight all other interactions. **B** The γ-carbon of the lactam side chain in P1 and the benzylic position of the P4 substituent are separated by a distance of 8.6 Å. **C** The β-carbon of the cyclopropyl residue from the P2 site and the amide nitrogen atom of the P1’ site are separated by a distance of 6.2 Å.

**Fig. 3:**
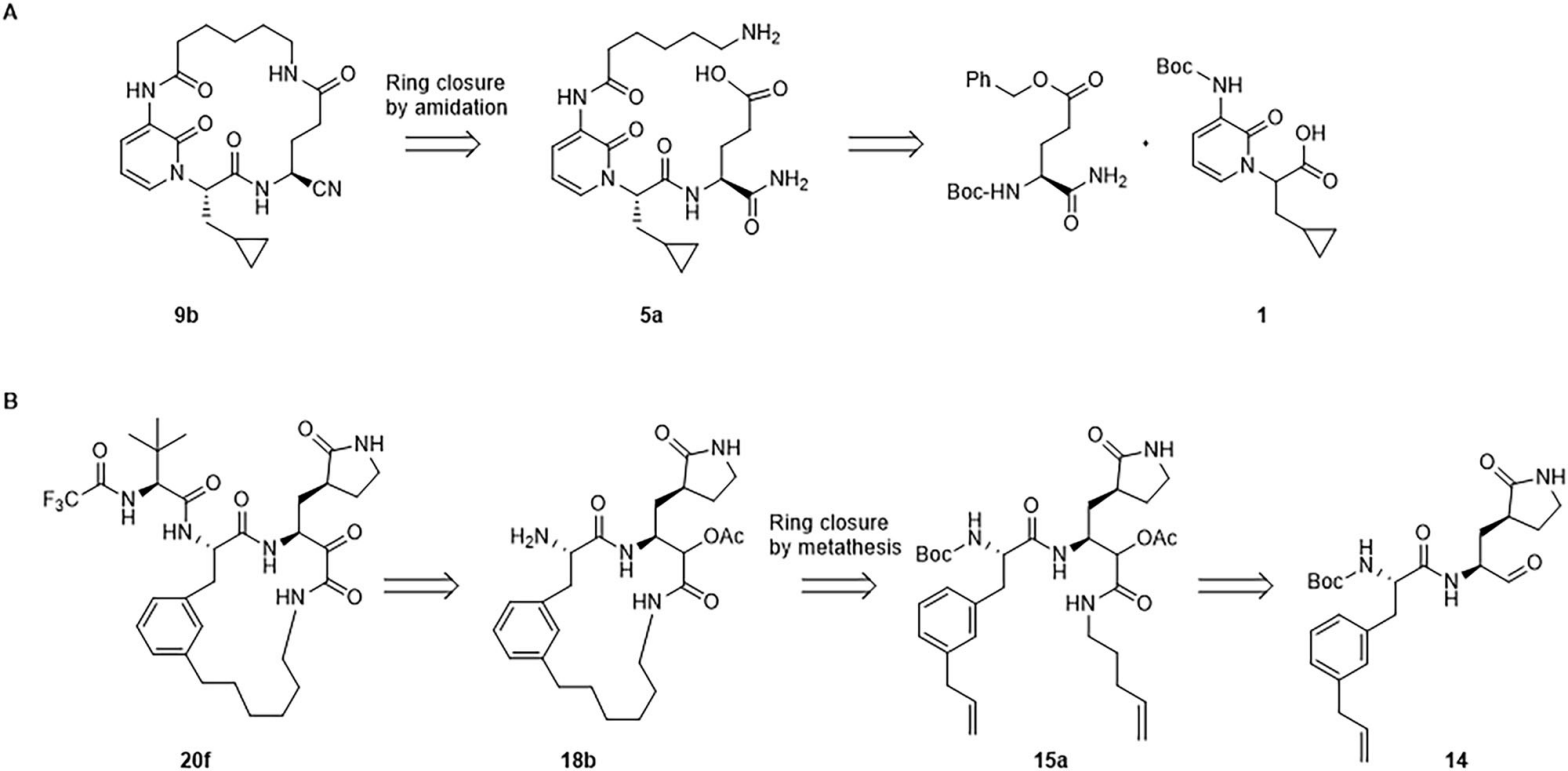
Structures and retrosynthesis of M^pro^-targeting macrocycles. **A** P1–P3-linked macrocycles, exemplified by **9b**, with an exocyclic nitrile warhead. **B** P2-P1’-linked macrocycles, exemplified by **20f**, with an endocyclic α-ketoamide warhead.

**Scheme 1:**
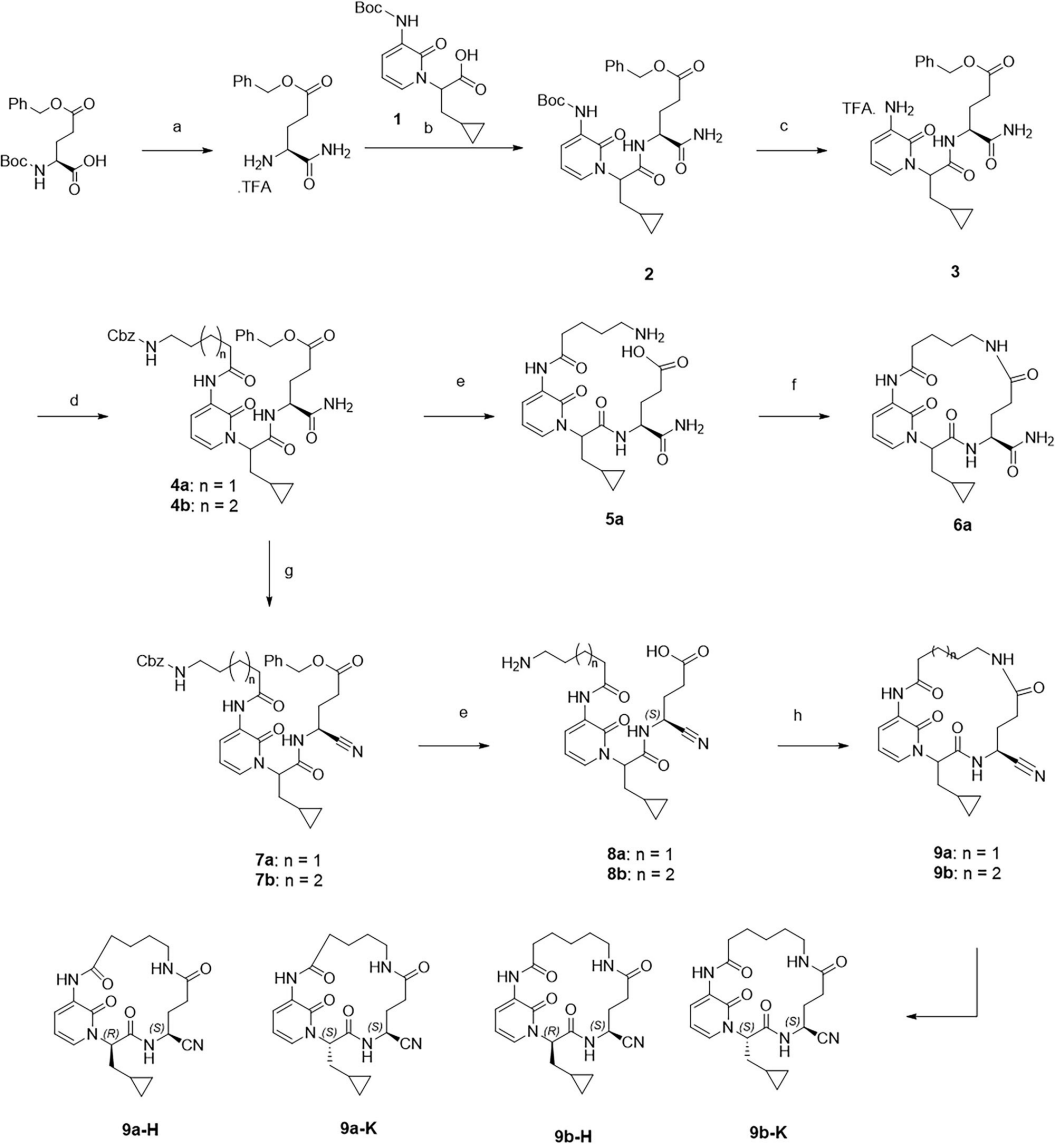
Synthesis of macrocyclic nitriles 9a-b^a^. ^*a*^**Reagents and conditions:** **a** 1. (BOC)_2_O/Py-NH_4_CO_3_, 1,4-dioxane, 2. TFA, CH_2_Cl_2_, 0 °C–rt, 5 h, quant. over two steps; **b** HATU/TEA, DMF, 0 °C–rt, 6 h, 90%; **c** TFA, CH_2_Cl_2_, 0 °C–rt, 5 h, quant.; **d** 5-(((benzyloxy) carbonyl) amino)pentanoic (or hexanoic) acid, HATU/TEA, DMF, 0 °C–rt, 10 h, 65–74%; **e** Pd/C, H_2_ gas, CH_3_OH, 5 h, 80%; **f** HATU/TEA, DMF, 0 °C–rt, 16 h, 75%; **g** Burgess reagent, DCM, rt, 4 h, 51–75%; **e** Pd/C, H_2_ gas, CH_3_OH, 5 h, 82–85%; **h** HATU/TEA, DMF, 0 °C–rt, 3 h, 43–64%.

A close inspection of crystallographic data on **12c** bound to M^pro^ (PDB: 8AIZ) revealed an 8.6 Å spatial separation between the γ-carbon of the lactam side chain in P1 and the benzylic position of the P4 substituent (Fig. 2A, B). This observation suggested that a P1–P3 linkage by macrocyclization to enhance conformational rigidity and preorganization for target binding might be feasible. For this purpose, glutamine was chosen to occupy the P1 position, as in natural substrates of M^pro^. We selected nitrile as a less reactive covalent warhead, because its high efficacy has been clinically validated (e.g., in nirmatrelvir^6^ or simotrelvir^8^), and because the synthesis could build on readily available L-glutamate. This led to the P1–P3-linked macrocyclic nitriles as target structures for synthesis (Fig. 3A and Scheme 1).

When assuming a different perspective on the co-crystal structure of **12c** to M^pro^ (PDB: 8AIZ), it became evident that also the β-carbon of the cyclopropyl residue from the P2 site and the amide nitrogen atom of the P1’ site were separated by a distance of only 6.2 Å (Fig. 2C). This implied that a second macrocyclization was possible between those sites. For practical reasons, we replaced the cyclopropane residue of P2 with a phenyl ring. Such a change was tolerated according to previous data obtained with the peptidomimetic **11a**^26^ on the M^pro^ of SARS-CoV. Moreover, a co-crystal structure between **11a** and M^pro^ suggested that a *meta*-substitution of the aryl ring would be optimal for a linkage to the P1’ site. For the latter, structural data, with our as well as other ketoamides (e.g., leritrelvir, Fig. 1), gave confidence that an alkyl chain was tolerated at the ketoamide nitrogen. In order to optimize interactions with the P4 site, the *N*-terminal amine was “capped” with different moieties, such as phenylacetyl that were identified in our previous optimization studies^40^. We therefore aimed synthesizing the P2-P1’-linked macrocycles as depicted in Fig. 3B.

### Synthesis of macrocyclic nitriles with linked P3 and P1 residues

Our retrosynthetic plan for the synthesis of macrocyclic nitriles envisioned an amide coupling with HATU under basic conditions for the ring closure and a dehydration reaction with Burgess reagent to convert the exocyclic amide to a nitrile (Fig. 3A). The central intermediate **5a** would be derived from an amide coupling reaction with the protected pyridone-derived dipeptide acid and the glutamatic acid amide.

In the forward direction, *N*-Boc-D-Glu(OBn)-OH was converted to the corresponding amide by treating it with Boc anhydride, pyridine, and ammonium carbonate in 1,4-dioxane at room temperature for 16 h (Scheme 1)^41^. Subsequently, the amide was deprotected to afford (S)-1-amino-5-(benzyloxy)-1,5-dioxopentan-2-aminium trifluoro acetate in quantitative yields. The latter^42^ was treated with the pyridone-containing peptidomimetic dipeptide **1**^40^ to the tripeptide **2** in 90% yield using HATU/TEA coupling conditions in DMF. Following deprotection of the Boc-group with TFA in DCM to give **3**, the tetrapeptides **4a**, **b** were obtained under optimized acid–amine coupling conditions with the ω-amino-substituted carboxylic acids.

The fully protected amide **4a** was transformed into the corresponding de-protected intermediates by treatment with Pd/C under a hydrogen atmosphere for 5 h, affording **5a** in 80% yield. Intermediate **5a** was then cyclized to the macrocyclic amide using standard peptide coupling conditions under high dilution to minimize dimer formation, yielding the desired macrocycle **6a** in 75% yield. However, attempts to convert the exocyclic amide into the corresponding nitrile using Burgess reagent in various solvents were unsuccessful, likely due to the high polarity of the compounds. Also, the use of other dehydrating agents such as TFFA/DMF was unsuccessful. Consequently, we adopted an alternative synthetic approach, converting the fully protected amides **4a**, **b** to the corresponding nitriles **7a**, **b** in 51–75% yields. The nitriles were de-protected to the corresponding free amino acids (82–85% yield) and then successfully cyclized to the desired macrocyclic nitriles **9a**, **b** with 43–64% yields. At the final stage, the respective diastereomers were separated and isolated using preparative HPLC purifications.

**Fig. 4:**
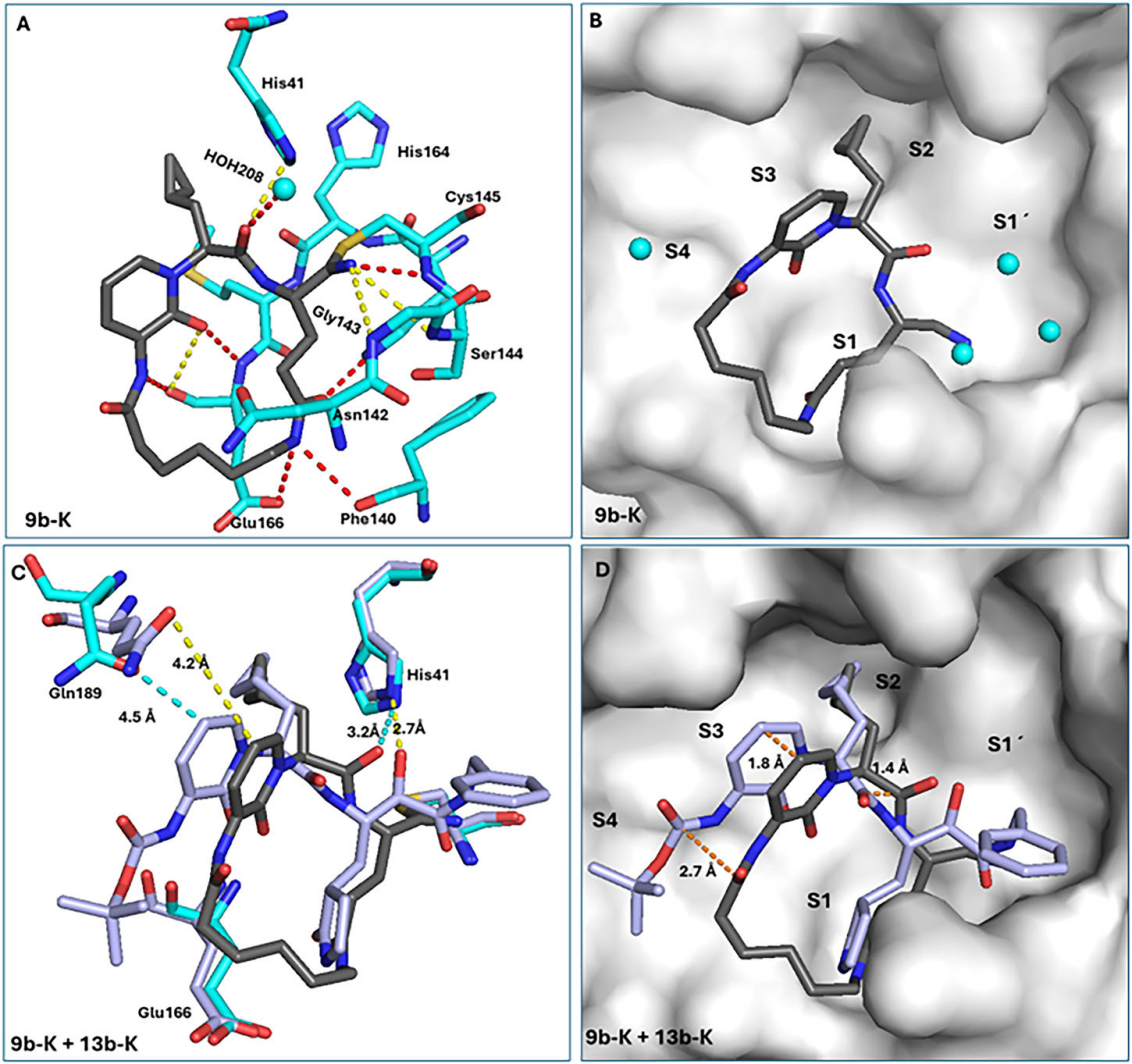
Co-crystal structure of M^pro^ in complex with 9b-K (PDB: 9SSN). (**A** Shown as sticks, **B** as surface) Amino acids (sticks) and water molecules (spheres) are colored in cyan, compound **9b-K** is colored in gray, and all H-bonds between the inhibitor and the corresponding M^pro^ residues are colored in red. Yellow dashes highlight all other interactions. **C**, **D** Superposition of the **9b-K** (colored in gray) with **13b-K** (colored in light blue), with key differences in the placement given by orange dashed lines.

A stereochemical assignment was made with the help of a co-crystal structure of **9b-K** in complex with M^pro^, that revealed an (*S,S*)-configuration for that diastereoisomer (Fig. 4). In consequence, **9b-H** possesses an (*R,S*)-configuration. We then identified the stereochemistry of the **9a** derivatives using NMR spectroscopy. The **9b-K** macrocycle displayed a triplet at 5.06 ppm (t, *J* = 7.7 Hz, 1H) together with a doublet of doublets at 4.97 ppm (dd, *J* = 9.9, 4.7 Hz, 1H). In contrast, the **9b-H** macrocycle showed two distinct doublets of doublets at 4.99 ppm (dd, *J* = 9.7, 5.8 Hz, 1H) and 4.65 ppm (dd, *J* = 10.2, 4.0 Hz, 1H) in the same region. The corresponding amide NH protons exhibited only minor chemical shift perturbations upon a change of stereoconfiguration (8.21 and 8.17 ppm). Similarly, in the **9a-K** macrocycle, the two CH protons appeared as a single multiplet at 4.93 ppm (2H), whereas in the **9a-H** isomer, these protons resonated as two separate doublets of doublets at 4.99 and 4.65 ppm (1H each), respectively. Thus, matching the chemical shifts observed in the **9b** pair to those in the **9a** pair allowed the assignment of the stereochemistry. The downfield NH proton at ~8.2 ppm was present in both **9a** isomers as well, with only minor differences in chemical shift and coupling pattern (Supporting Information, S11–14).

### Functional and structural characterization of interactions between macrocyclic nitriles and M^pro^

The macrocyclic nitriles were characterized with respect to their ability to inhibit the proteolytic activity of SARS-CoV-2 M^pro^. For this purpose, the compound-mediated reduction in the cleavage rate of a model substrate was measured. Among the tested compounds, only **9b-K** exhibited a measurable inhibition of 62% at 20 µM, while **9a-H,** **9a-K**, and **9b-H** were inactive (enzyme inhibitory activities in Table S1). In spite of the moderate potency observed for **9b-K**, we determined its co-crystal structure in complex with M^pro^ at a resolution of 1.5 Å, in order to shed light on its binding mode (Fig. 4).

The association of **9b-K** was strengthened by 11 hydrogen bonds to M^pro^ residues. Moreover, the sulfur atom of Cys145 bound covalently to the P1´carbon of 1.8 Å in the S1´ subsite. An overall structural alignment of the **9b-K** (9SSN) with **13b-K** (PDB: 6Y2G) reveals root mean square (r.m.s.) deviations of 1.4, 1.8, and 2.7 Å for the Cα positions in the S2, S3, and S4 subsites, respectively (Fig. 4D). The P1´ imine occupied the oxyanion hole and make three interactions with the backbone amides of Cys145 (2.9 Å), Ser144 (3.5 Å; 3.0 Å for **13b-K**) and Gly143 (3.5 Å; 3.0 Å for **13b-K**). The adjacent amide oxygen formed a hydrogen bond with a water molecule HOH208 (2.9 Å) and a weak interaction with His41 Nε2 (3.2 Å; 2.7 Å for **13b-K**). In the S1–S2 subsite, hydrogen-bonding interactions to His163 Nε2 (2.8 Å; 2.6 Å for **13b-K**), the backbone carbonyl oxygen of Phe140 (3.1 Å; 3.0 Å for **13b-K**) and the Glu166 Cε2 remained despite the absence of the γ-lactam. In the S3 subsite, the pyridone played the same role as **13b-K** and make strong interaction with the backbone amide (2.9 Å) of Glu166 and weak interaction with the backbone carbonyl (3.6 Å; 3.4 Å for **13b-K**) of the same residue. However, the interaction to Gln189 became weaker in **9b-K** (4.5 Å vs. 4.2 Å for **13b-K**). Finally, the P3 amide nitrogen of **9b-K** interacted also with main chain carbonyl of Glu166 (3.3 Å; 3.0 Å for **13b-K**). In summary, the macrocyclic ring of **9b-K** showed only partial fits to the S3-S4 subsite, and multiple interactions were weaker in comparison to for **13b-K**; this experimental binding mode thus provides an explanation for the poor inhibitory potency of **9b-K**.

### Synthesis of macrocyclic α-ketoamides with linked P1’ and P2 residues

To obtain macrocycles with an endocyclic α-ketoamide linking P1’ and P2 residues, our retrosynthesis envisaged the attachment of the P4 residue in the last step (Fig. 3B). The ring closure should involve a Grubbs metathesis reaction from the open-chain intermediate **15a**. The latter should be obtained through a Passerini reaction between the aldehyde **14** and a terminally unsaturated isonitrile. A variation of the isonitrile chain length would allow an adjustment of the macrocyclic ring size.

**Scheme 2:**
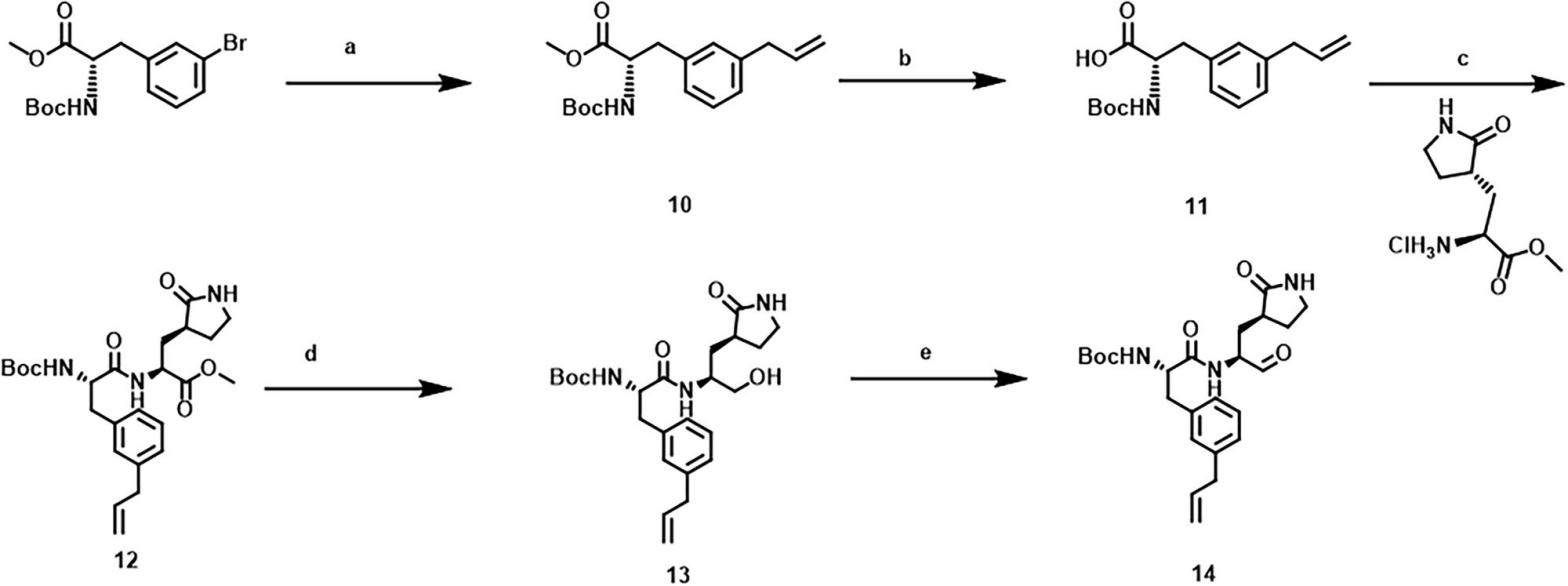
Synthesis of intermediate aldehyde 14. ^*a*^Reagents and conditions: **a** Pd(PPh_3_)_4_, CsF, 2-allyl-4,4,5,5-tetramethyl-1,3,2-dioxaborolane, THF, reflux, 17 h, 93%; **b** LiOH.H_2_O, aq.CH_3_OH, 2 h, 80%; **c** HATU/TEA, DMF, 0 °C–rt, 16 h, 74%; **d** NaBH_4_, CH_3_OH, 5 h, 82%; **e** DMP, CH_2_Cl_2_, rt, 3 h, 65%. Note that **11,** **12**, and **13** are different from the previously reported compounds **11a,** **11r,** **12c**, or **13b-K** shown in Fig. 1.

**Scheme 3:**
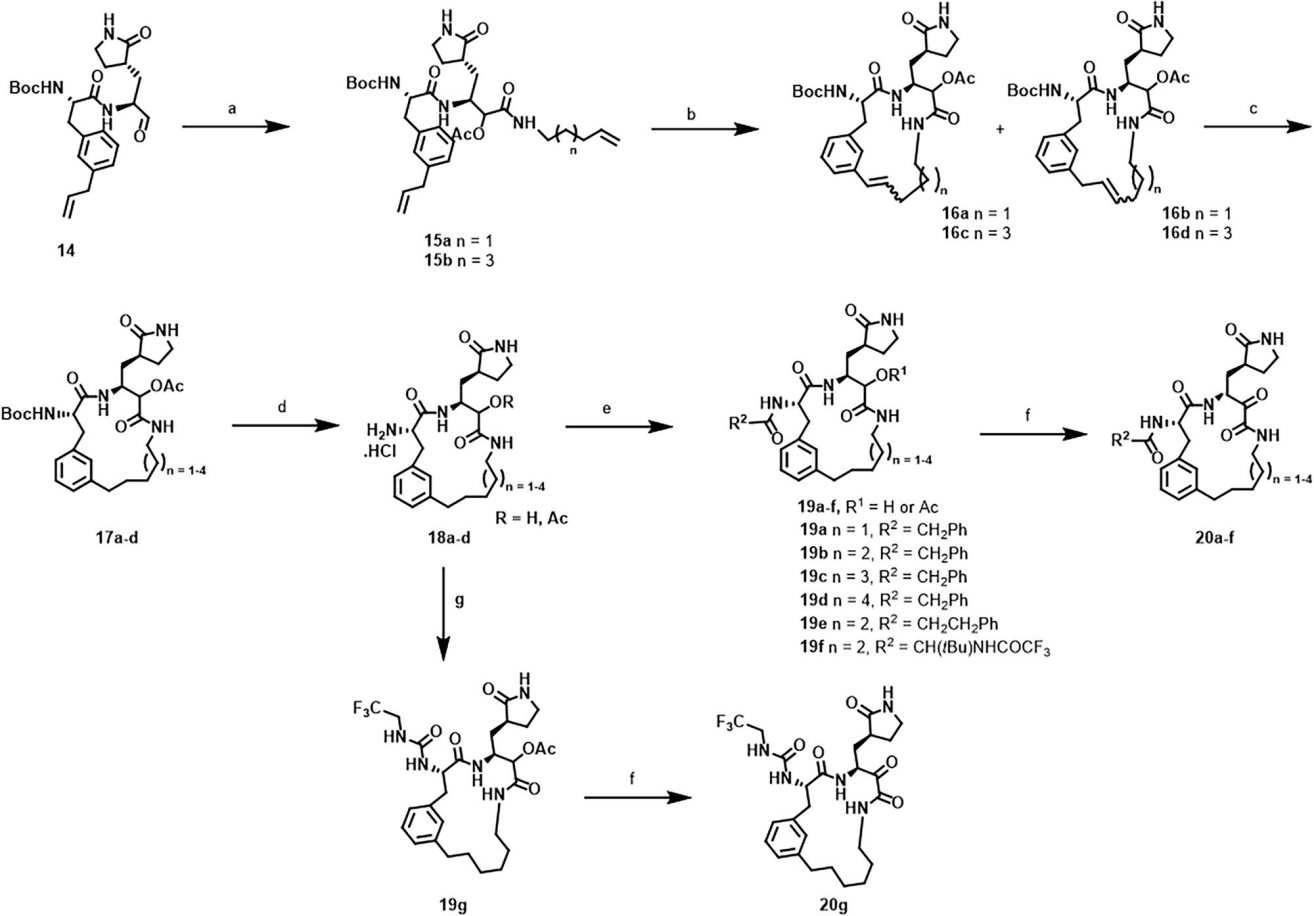
Synthesis of macrocyclic α-ketoamides 20a–g. ^*a*^Reagents and conditions: **a** alkenyl isonitriles, CH_3_COOH, CH_2_Cl_2_, rt, 17 h, 55–57%; **b** Hoveyda–Grubbs II, ClCH_2_CH_2_Cl, 18 h, 43–54%; **c** Pd-C (10%), H_2_ gas, rt, 5 h, 82–87%; **d** Conc. HCl, CH_2_Cl_2_, 0 °C–rt, 5 h, 65–93%; **e** R-COOH, HATU, TEA, DMF, 0 °C–rt, 3 h, 35–63%; **f** (i) LiOH.H_2_O, CH_3_OH, H_2_O, 0 °C–rt, 1.5 h; (ii) DMP, DMF, rt, 2 h, 53–69% over two steps; **g** CF_3_CH_2_NCO, DIPEA, DMF, 0 °C–rt, 24 h, 53%.

**Scheme 4:**
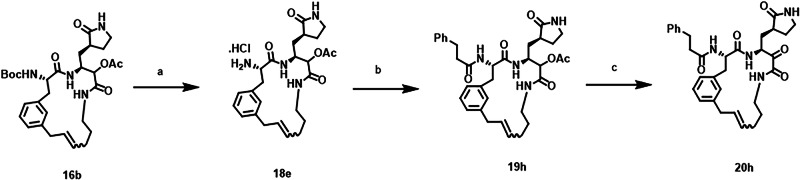
Synthesis of macrocyclic α-ketoamide 20 h. ^*a*^Reagents and conditions: **a** 4 M HCl, CH_2_Cl_2_, 5 h, 73%; **b** 3-phenylpropanoic acid, HATU, TEA, DMF, 0 °C–rt, 5 h, 48%; **c** (i) LiOH.H_2_O, CH_3_OH, H_2_O, 0 °C– rt, 1.5 h; (ii) DMP, DMF, rt, 2 h, 65% over two steps.

In the forward direction, the synthesis started with the allylation of Boc- and methyl-protected *m*-bromophenylalanine^43^ through a Suzuki–Miyaura cross-coupling to give the intermediate **10** (Scheme 2). The use of CsF as a base was important for an efficient outcome of the reaction^44^. Reaction conditions were further optimised to use *m*-bromo phenylalanine ester with less catalyst loading. Alternatively, a Stille coupling with allylstannane, conducted on a gram scale, gave comparable yields (Supporting Information S5). Following hydrolysis with lithium hydroxide, the carboxylic acid **11** was obtained in a yield of 80%. The intermediate **11** was coupled with methyl-(*S*)-2-amino-3-((*S*)-2-oxopyrrolidin-3-yl)propanoate HCl salt to give the dipeptide ester **12** using HATU in TEA as a base (74% yield). The ester functionality in **12** was further converted to the aldehyde **14** in two steps^40^. The aldehyde **14** represented the last common intermediate before diversification. It was reacted with pent-4-en-isonitrile or hept-5-en-isonitrile and acetic acid in a Passerini reaction to give the acetoxy amides **15a** and **15b**, respectively (Scheme 3). Macrocyclization was achieved by a ring-closing metathesis reaction using the Hoveyda–Grubbs-II catalyst, which converted **15a** to the macrocyclic **16b** as the major product as a mixture of *E/Z* diastereomers in a yield of 54%. In addition, **16a**, whose cycle was shortened by one methylene unit, was formed as a side product in a yield of 9%. We assume that the Ru catalyst induced an isomerization of the allyl-substituted phenyl ring to a styrene prior to the metathesis reaction^45^. The equivalent reaction with **15b** gave the products **16d** and **16c**. Because main and side product could be separated by prep HPLC in both pairs, macrocycles of four different ring sizes **16a–d** were available for SAR studies. The alkenes **16a–d** were hydrogenated at room temperature, catalyzed by palladium on carbon (10%) to the corresponding alkanes **17a–d** in yields of 82–87%. Upon removal of the Boc group to give **18a–d**, a partial hydrolysis of the α-acetoxy moiety to α-hydroxyamides occurred, which were tolerated at this stage. The free primary amine was then equipped with P4 substituents. For this purpose, phenylacetic acid, a P4 group that was favorable in linear α-ketoamides^40^, was activated with HATU and TEA over 3.5 h and reacted with all four macrocycles to give **19a–d**. Three more analogs were prepared starting from intermediate **18b**. The introduction of a larger phenylpropionyl moiety as an S4 group led to **19e**, the use of trifluoroacetyl-*tert*-leucine, reflecting the P3/P4 groups of nirmatrelvir, gave **19f**, and the reaction with 1,1,1-trifluoro-2-isocyanatoethane gave the urea **19g**. The last steps comprised the complete basic hydrolysis of the acetoxyamides and a Dess–Martin oxidation to the final α-ketoamides **20a–f** in yields of 53–69% over two steps. In order to probe whether the reduction of the double bond from **16** to **17** was beneficial or detrimental, it was kept in **20h**. Analog **20h** carried the phenylpropionyl side chain and was prepared as an *E/Z* mixture as shown in Scheme 4. All compounds were analytically characterized by NMR, HR-MS, and HPLC. In sum, macrocyclic α-ketoamides were prepared in 11–12 steps with typical overall yields of 3.2–1.5%.

### Structural characterization of interactions between macrocyclic α-ketoamides and M^pro^

**Fig. 5:**
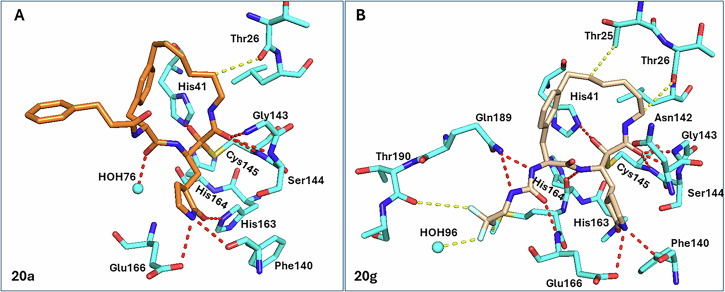
Overall co-crystal structure of Mpro in complex with 20a (A PDB: 9T5E) and with 20g (B PDB: 9SSO). **A** Amino acids (sticks) and **B** water molecules (spheres) are colored in cyan, **20g** is colored in gold and **20a** is colored orange. All H-bonds between the inhibitor and the corresponding M^pro^ residues are colored in red. Yellow dashes highlight all other interactions.

**Fig. 6:**
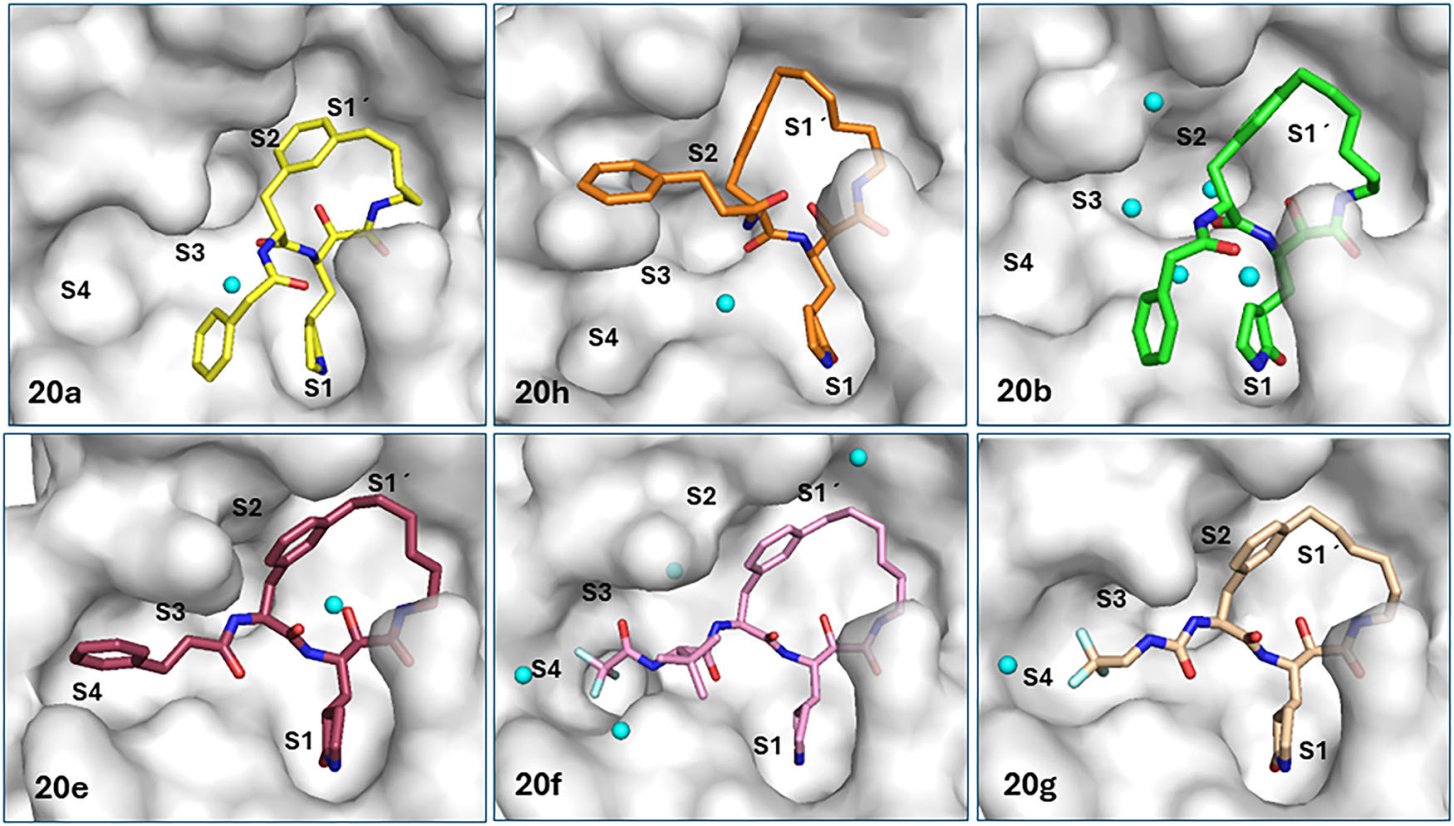
Co-crystal structure of macrocyclic ketoamides 20a (PDB: 9T5E), 20h (PDB: 9T72), 20b (PDB: 9T52), 20e (PDB:9T55), 20f (PDB: 9T5F) and 20g (PDB: 9SSO). The views illustrate the occupation of the S4 pockets on the left sides by **20e–g**.

Purified SARS-CoV-2 M^pro^ was co-crystallized with the macrocycles **20a**, **20b**, and **20e**–**h** (Figs. 5 and 6, data collection and refinement statistics in Tables S2 and S3). The crystal structures of all inhibitors in complex with M^pro^ revealed that the sulfur atom of Cys145 formed a covalent bond (1.7–1.9 Å) with the electrophilic carbon of the P1´, establishing an *R*-configuration. Within the S1´ subsite of the enzyme, the P1´ carbonyl oxygen occupied the oxyanion hole and accepted three hydrogen bonds (2.8–3.3 Å) from the backbone amides of Cys145, Ser144, and Gly143 for all inhibitors (Figs. 5 and 6). The thiohemiketal formed a strong hydrogen bond (2.5–2.7 Å) with His41 Nε2 for **20b** and **20e–g**, but a weak interaction for **20a** and **20h**. All macrocyclic inhibitors showed a weak interaction of 3.3–3.6 Å between the macrocyclic ring and the Cγ2 of Thr25. Furthermore, a weak interaction (3.3–3.6 Å) was observed between the oxygen atom of the carbonyl group of Thr26 and the macrocyclic ring of all inhibitors except **20b** and **20h**. This suggests a relative decrease in the flexibility of the macrocyclic ring due to the additional double bond in **20h**. While **20h** was prepared as a mixture of *E-* and *Z-* diastereomers, only the *E-*isomer co-crystallized with M^pro^.

With regards to the S1 and S2 subsites, all macrocycles formed interactions to His163 Nε2 (2.6 Å), to the backbone carbonyl oxygen of Phe140 (3.1–3.3 Å) and to the carboxylate of Glu166 (3.1–3.3 Å). However, interactions between the endocyclic main chain amide and the carbonyl oxygen of His164 (3.0–3.1 Å) as well as the hydrogen bond between the carbonyl oxygen of the inhibitor and the backbone amide of Glu166 (2.7–2.9 Å), were only found for **20f,** **20g**, and **20e**.

Finally, only **20e–g** occupied the S4 subsite, although **20e** did not engage in specific hydrogen-bonding interactions (Fig. 6). **20g** showed additional hydrogen-bonding interactions to Gln189 Oε1 (2.9 Å) and Gln189 Nε2 (3.0 Å). In contrast, a water molecule (HOH 98) formed a bifurcated hydrogen-bond with the main-chain amide of **20f** (3.0 Å) and the Gln189 Oε1 (2.7 Å).

### In vitro activities

The ability of the α-ketoamides to inhibit the enzymatic activity of M^pro^ was tested next. The analogs **20a–d** all had a phenylacetyl residue, but the alkyl linker between the aryl and the amide moieties varied systematically, covering pentyl, hexyl, heptyl or octyl groups. These four analogs inhibited the enzyme with IC_50_ values of 8.2, 1.37, 3.0, and 4.57 µM, respectively (Table 1). Thus, the size of the macrocycle had a clear impact on activity, and the hexyl linker, corresponding to a 17-membered macrocycle, was optimal. Therefore, subsequent analogs were prepared with this ring size. Also, the nature of the P4 residue turned out to be relevant for activity: The elongation of the phenylacetyl group to phenylpropionyl as in **20e** led to an increase of activity from 1.37 to 0.5 µM. The urea **20g** and the *tert*-leucine analog **20f** were the most potent analogs in the series with IC_50_’s of 0.36 and 0.37 µM, respectively. This increase in potency can be traced back to their ability to populate the S4 site, as observed in the co-crystal structures. An unsaturated hexenyl linker was incorporated in analog **20h**, carrying a phenylpropionyl moiety in the P4 position. With an activity of 2.2 µM, the analog was weaker than the saturated analog **20e** (IC_50_ = 0.5 µM). This may reflect the unfavorable orientation of alkene functionality in the S1’ and S2 subsites and the reduced number of interactions observed in the co-crystal structure. In summary, the SAR studies showed that a 17-membered, saturated macrocycle was an optimal M^pro^ inhibitor, with analogs populating S4 sites such as **20e**–**g** reaching activities in the nanomolar range.

**Table 1:** Enzyme inhibition, antiviral activities and cytotoxicity of macrocyclic α-ketoamides


Cmpd	L	R	Ring size	M^pro^ IC_50_^a^ (µM)	SARS-CoV-2 EC_50_^b^ (µM)	CC_50_^c^ (µM)	EV-D68 3C^pro^ IC_50_^a^ (µM)	EV-D68 EC_50_^d^ (µM)
**20a**	pentyl		16	8.2 ± 1.9	n.d.	n.d.	17% inh. @100 µM	n.d.
**20b**	hexyl		17	1.37 ± 0.36	23.3 ± 3.9	>50	2.8 ± 0.6	5.0 ± 0.7
**20c**	heptyl		18	3.0 ± 0.69	n.d.	n.d.	10% inh. @100 µM	n.d.
**20d**	octyl		19	4.57 ± 0.73	n.d.	n.d.	2.9 ± 0.4	n.d.
**20e**	hexyl		17	0.50 ± 0.09	7.0 ± 2.7	>50	1.4 ± 0.2	4.3 ± 0.3
**20f**	hexyl		17	0.37 ± 0.07	1.9 ± 0.3	>50	2.7 ± 0.2	0.033 ± 0.004
**20g**	hexyl		17	0.36 ± 0.12	>50	>50	2.9 ± 0.4	1.5 ± 0.1
**20h**	hex-2-enyl^e^		17	2.2 ± 0.68	47.6 ± 1.7	>50	n.d.	n.d.
Nirmatrelvir^f^				0.015 ± 0.07	0.07 ± 0.02	>10	46.8 ± 25.5	n.d.
Rupintrivir				>30	n.d.	78.9 ± 11.0	0.28 ± 0.03	0.005 ± 0.001

^a^ ± standard deviation (SD), *n* = 3.

^b^ tested on SARS-CoV-2-ZG/A549^ACE2+TMPRSS2^ cells.

^c^ tested on A549^ACE2+TMPRSS2^ cells by Cell Counting Kit-8; n.d.-not determined, ± standard deviation (SD).

^d^ tested on RD cells, ± standard deviation (SD).

^e^ tested as an E/Z mixture.

^f^ Taken from ref. ^41^.

### Antiviral activities against SARS-CoV-2

Based on the above SAR studies, we further evaluated the antiviral activities of selected macrocyclic α-ketoamides. For this purpose, human A549 lung alveolar epithelial cells transfected with ACE2 + TMPRSS2^46^, referred to as A549-AT cells herein, were infected with a SARS-CoV-2 wildtype strain. Their ATP content was quantified by CellTiterGlo reagent to detect virus-mediated cytopathic effects and their prevention by the inhibitors. We found that **20b**, equipped with a hexyl linker and an *N*-terminal phenylacetyl group, exhibited a moderate activity with an EC_50_ of 23.3 µM. The change of the *N*-terminal group had drastic effects on activity: A phenylpropionyl group was considerably more potent (EC_50_ = 7.0 µM). In view of its high in vitro potency, we were surprised to find the urea **20g** as inactive at concentrations up to 50 µM. Analog **20h**, carrying a phenylpropionyl group and a hexenyl linker, also displayed little activity (EC_50_ = 47.6 µM). The most potent macrocycle was the trifluoroacetyl-*tert*-leucine analog **20f** with an EC_50_ of 1.9 µM (Table 1).

### Cellular accumulation

To get a deeper understanding of the links of biochemical and cellular activity, intracellular concentrations of selected macrocyclic α-ketoamides were determined in A549-AT cells and utilized to calculate cellular accumulation (*K*_*p*_ values) by taking the cell number and extracellular compound concentration into account. Additionally, nonspecific binding during the experiments was considered to receive the final *K*_*p*_ values (Table 2 and cellular accumulation data in Table S6). We observed intracellular accumulation for inhibitors **20b,** **20e,** **20f**, and **20h**, as reflected by *K*_*p*_ values greater than 1, with compounds **20e** (*K*_*p*_ = 3.57) and **20h** (*K*_*p*_ = 3.50) showing the highest intracellular levels. Similar to the cellular antiviral activity, the *N*-terminal phenylpropionyl group in **20e** increased cellular accumulation in comparison to the phenylacetyl group contained in **20b**. Despite its potent antiviral activity, the tested intracellular concentration of **20f** was moderate with a resulting *K*_*p*_ value of 1.67. The urea analog **20g**, inactive in cellular antiviral studies against SARS-CoV-2 despite potent biochemical activity, was found to be the only derivate which did not accumulate in cells (*K*_*p*_ = 0.85). This potentially explains the weak cellular activity. Overall, the cellular accumulation were not predicted in the same rank order as cellular antiviral activities. However, cellular accumulation did not account for protein binding in the cytosol or distribution into sub-cellular organelles. It is also conceivable that uptake is altered under infection conditions. These non-quantified contributors to target engagement might account for the differences in rank order.

**Table 2:** Cellular accumulation of selected macrocyclic α-ketoamides

Cmpd	*K*_*p*_ value^a^
**20b**	2.42
**20e**	3.57
**20f**	1.67
**20g**	0.85
**20h**	3.50

^a^tested in A549^ACE2+TMPRSS2^ cells. See also Table S6.

### Expansion of enzymatic and antiviral activities of macrocycles to enteroviruses

**Fig. 7:**
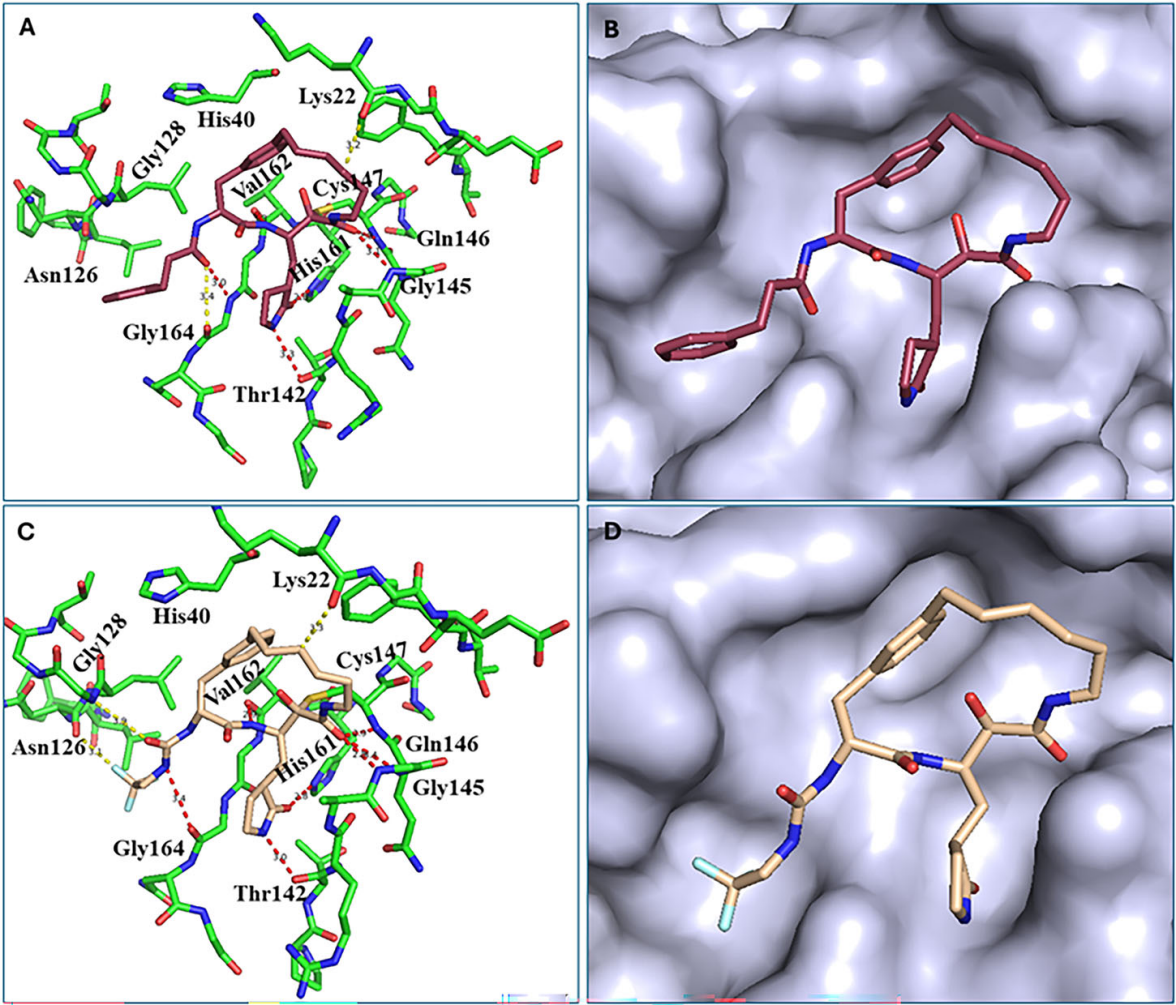
Co-crystal structure of macrocyclic ketoamides in complex with 3C^pro^ of EV-D68. Upper panels show the complex with **20e** (**A** shown as sticks, **B** as surface; PDB:29CT), lower panels show the complex with **20g** (**C** shown as sticks, **D** as surface; PDB:29DA).

The main protease M^pro^ of SARS-CoV-2 and the 3C proteases (3C^pro^) of enteroviruses share structural similarities, are both cysteine proteases and cleave after glutamine residues. A distinction lies within the S2 binding pocket, which is enclosed in M^pro^, whereas it is comparatively open in 3C^pro^ and preferentially recognizes phenylalanine rather than leucine residues at this position^25^. Because the macrocyclic ketoamides incorporate a phenylalanine residue at P2, we hypothesized that tight binding interactions to 3C^pro^ should be feasible. To verify this, the purified EV-D68 3Cpro was also co-crystallized with the macrocycles **20e** and **20g** (Fig. 7, data collection and refinement statistics in Table S3). The crystal structures of both inhibitors in complex with 3C^pro^ of enterovirus D68, solved at resolutions of 1.91 and 2.08 Å, respectively, reveal that the sulfur atom of Cys147 formed a covalent bond (1.8 Å each) with the electrophilic carbon of the P1´, analogous to the SARS-CoV-2 M^pro^ complex. Within the S1´ subsite of the enzyme, the P1´ carbonyl oxygen occupied the oxyanion hole and accepted three hydrogen bonds (2.8–3.4 Å) from the backbone amides of Cys147, Gln146 and Gly145 for the complex with **20g**, but only two hydrogen bonds for the complex with **20e** (from Cys147 and Gly145) (Fig. 7). The strong hydrogen bond between the thiohemiketal and His41 shown in complex with M^pro^ was lost for corresponding His40 residue in the 3C^pro^ complex, because His40 was oriented outside the pocket. The γ-lactam in the S1 pocket had only two hydrogen bonds with His161 (3.0–3.3 Å) and Thr142 (3.0–3.3 Å) for **20e** and **20g**, whereas the complex with M^pro^ was stabilized by three hydrogen bonds (to His163, Phe140, and Glu166). In the S2 subsite, the endocyclic phenyl groups of **20e** and **20g** extend more deeply into the pocket compared to the M^pro^ complexes, where the phenyl groups face outwards: The distance between the endocyclic phenyl groups of **20g** or **20e** and the first residue in the S2 subsite was larger for M^pro^ (5.1 and 4.9 Å to Met49, respectively) than for 3C^pro^ (4.0 and 3.8 Å to Pro38, respectively) (Structures with comparisons of the binding modes in Fig. S2). This improved occupation reflects the substrate specificity for Phe in 3C^pro^, while M^pro^ recognized Leu at this position. In the S2 and S3 subsites, interactions between the carbonyl oxygen of the inhibitor and the main chain amide of Val162 (3.0–3.1 Å) as well as a hydrogen bond to Gly164 (3.0–3.4 Å) were observed for both **20e** and **20g**. M^pro^-complexes had corresponding interactions to His164 and His163, respectively. Finally, two weaker interactions between the trifluoroacetyl group of **20g** and Gly128 (3.8 Å) and Asn126 (3.4 Å) were present in the S3-S4 subsite, but not with the phenylpropionyl group of **20e**. In summary, the structural characterization of ketoamide macrocycles demonstrated multiple similarities in their binding to 3C^pro^ and M^pro^. While less interactions were exerted at the S1 and S1’ sites of 3C^pro^, the occupancy of the S2 site was improved. This provides a rational and understanding why the compounds may act as dual inhibitors of these distinct viral proteases.

In order to provide functional evidence, we probed the ability of the macrocycles to inhibit the 3C^pro^ of the enterovirus D68 in a FRET-based enzymatic assay. Five compounds inhibited the 3C^pro^ of D68 with IC_50_ values of 1.4–2.9 µM (Table 1). For **20b** and **20e**, we could also show inhibition of the 3C^pro^ of the enterovirus EV-A71 with IC_50_ values of 6.9 and 4.4 µM, respectively (enzymatic inhibition data in Table S7). The most potent inhibitor was **20e**, carrying a hexyl linker and a phenylpropionyl group at the P4 position. Thus, a dual inhibition of coronaviral and enteroviral proteases could be achieved. Beyond that, we also observed inhibition of human cathepsins, structurally related cysteine proteases, for the tested inhibitors **20b,** **20e**, and **20h** (inhibition of human cathepsins in Table S8). The inhibition was particularly strong for cathepsin L. Because cathepsin L plays a role in the entry of SARS-CoV-2 Omicron variants, such as the BA.5 strain, its inhibition has been regarded as favorable to enhance the antiviral effect^9,47^; other cathepsins are rather seen as off-targets.

We also tested macrocycles from the first series with an exocyclic nitrile warhead. Both **9b-K** and **9a-K** were inactive against EV-D68 3C^pro^. Thus, the insufficient activity of the exocyclic nitriles against M^pro^ was also reflected in the related enteroviral protease.

**Fig. 8:**
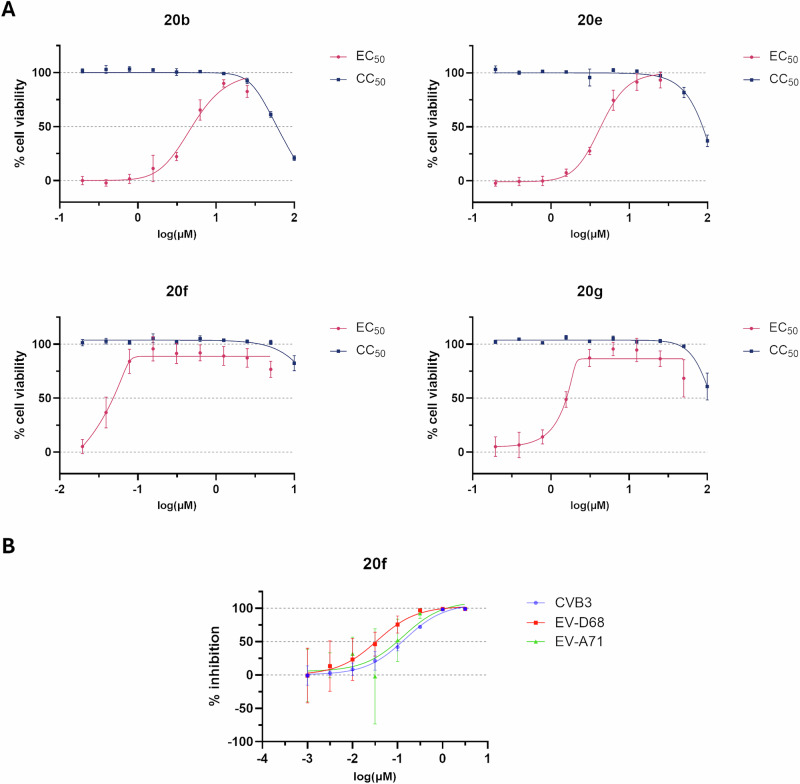
Antiviral effects of macrocyclic ketoamides on EV-D68, EV-A71 and Coxsackievirus-B3. **A** Concentration-response curves of the macrocyclic inhibitors **20b** and **20e–g**. The antiviral effect against EV-D68 in RD cells was measured by a cell viability assay using crystal violet. **B** Antiviral effects of **20f** detected by the luciferase activity of corresponding reporter viruses. All measurements were performed with *n* = 3 biologically independent experiments. The mean values are depicted with error bars representing the standard deviation (SD).

We finally tested the macrocycles **20b** and **20e**–**g** in cellular assays for anti-enteroviral activity. RD cells were infected with EV-D68, and the activity of the compounds was determined by a cell viability assay using crystal violet. The analogs **20b,** **20e** and **20 g** inhibited this virus with EC₅₀‘s of 5.0 ± 0.6, 4.3 ± 0.3, and 1.5 ± 0.1 µM, respectively. To our surprise, **20f** exhibited very potent activity with an EC₅₀ of 0.033 ± 0.004 µM against EV-D68 (Fig. 8A). We therefore expanded the profiling of **20f** in HeLa-R19 cells that were infected with EV-D68, EV-A71 and Coxsackievirus CVB3 reporter viruses. Again, **20 f** was able to potently inhibit these viruses with EC₅₀‘s of 0.035, 0.13, and 0.15 µM, respectively (Fig. 8B, antiviral effects of **20f** on EV-D68, EV-A71 and Coxsackievirus-B3 in Supplementary Fig. 3). Given that the cellular cytotoxicity of the compound was >100 µM, the selectivity indices (SI) of **20 f** for the antiviral activities against EV-D68, EV-A71 and Coxsackievirus CVB3 were >1030, >769, and >667.

In summary, an extension of macrocycles rationally designed M^pro^ inhibitors towards enteroviral 3C proteases was possible. It was associated with some loss of in vitro potency, but a remarkably high cellular antiviral activity in different assay systems for the analog **20f**.

### In vitro ADME properties of 20f

As **20f** exhibited broader spectrum activity against SARS-CoV-2 and three distinct enteroviruses, we were interested in its in vitro ADME properties. We found that **20f** was metabolically stable in both mouse and human microsomes and exhibited excellent plasma stability. **20f** showed high plasma protein binding of around 99% in mouse and 99.1% in human plasma (Table 3). Future studies would aim to lower plasma protein binding while preserving the excellent bioactivity profile.

**Table 3:** ADME properties of 20f

Property	Value
Cl_int_ [µl/min/mg protein] mouse	< 23
*t*_1/2_ [h] mouse microsomes	> 60
Cl_int_ [µl/min/mg protein] human	< 23
*t*_1/2_ [h] human microsomes	> 60
*t*_1/2_ [h] mouse plasma	> 240
*t*_1/2_ [h] human plasma	> 240
plasma protein binding mouse [%]	99.0 ± 0.3
plasma protein binding human [%]	99.1 ± 0.4

## Discussion

We explored the feasibility of macrocyclization for rationally designed inhibitors of the coronaviral main protease. Based on the previously reported linear compound **12c**, a first series of analogues bearing an exocyclic nitrile warhead and a macrocycle bridging P1 and P3 was designed. Four compounds were isolated as pure diastereomers by preparative HPLC and displayed only low inhibitory activity against M^pro^ of SARS-CoV-2, and none against 3C^pro^ of EV-D68. In spite of this, a co-crystallization of **9b-K** with M^pro^ at a high resolution of 1.5 Å was successful. The structure confirmed that the compound occupied the P1′ to P2 subsites as expected, and it also showed the intended covalent binding to the nitrile warhead. Thus, the structure provided support for our initial design concept. However, it also revealed important limitations. Multiple hydrogen bonds, namely to Gly143, Ser144, His41, Glu189, and Glu166, were elongated by 0.3 Å or more. Thus, the suboptimal fit or the lack of substituents engaging the S1/S3/S4 subsites significantly diminished overall potency. These observations provided clear direction for subsequent optimization efforts. On a general note, they show that characterizing protein-ligand structures even for poorly active compounds is both feasible and valuable. This implies to re-consider drug profiling workflows that often place structural biology experiments after filtering for biochemical activity, rather than conducting them in parallel.

Our second design hypothesis was that linking the P1′–P2 residues to a macrocycle while retaining an endocyclic ketoamide warhead could yield potent inhibitors. This led to the synthesis of macrocyclic α-ketoamides **20a–d**, featuring linkers of varying lengths from pentyl to octyl. Among these **20b** with a hexyl linker demonstrated an IC_50_ of 1.37 µM against M^pro^ and an antiviral EC_50_ of 23.3 µM. A key observation from co-crystal structures of **20a–d** was that a phenacetyl group did not properly occupy the S3/S4 subsites. We therefore designed **20e**, bearing a phenylpropionyl capping group, which showed an improved IC_50_ of 0.5 µM and a threefold increase in anti-SARS-CoV-2 activity compared with **20b**. Subsequent modifications as in **20f** and **20g** yielded improved IC_50_ values of 0.37 and 0.36 µM, respectively. Interestingly, **20g** failed to show measurable antiviral activity at 50 µM, highlighting that structural features beyond enzyme inhibition play an essential role. We assume that the presence of additional hydrogen-bonding groups impaired cell permeability, as observed experimentally (Table 2). A similar observation was made in our previous P3/P4 capping group optimization studies^40^, where most—but not all—ureas were inactive in cellular assays. In contrast, **20f** exhibited an antiviral EC_50_ of 1.9 µM against SARS-CoV-2, underscoring the importance of an optimally configured P3/P4 capping unit. The unsaturated cycle **20h** was less active than its saturated analogs **20e**. Moreover, because *E/Z* stereoselectivity could not be achieved in the synthetic route, saturated macrocycles were preferred. In sum, a successful macrocyclization of ketoamide-based M^pro^ inhibitors could be achieved. A clear advantage over the best linear compounds is not obvious at this stage. However, the macrocyclic scaffold itself has not been fully explored. While optimization efforts in this study mostly focused on finding the optimal length of an alkyl linker, discontinuous activity trends in both cellular and enzymatic assays as well as the crystal structures imply high conformational plasticity of the macrocyclic scaffold. The decoration of the cycle with heteroatoms or substituents thus offers further potential for improvements in activity through conformational restrictions, as shown in systematic macrocycle optimization programs against different targets^48,49^.

Given the (limited) similarities of 3CL^pro^ and 3C^pro^, we further evaluated the activities of macrocyclic α-ketoamides against the EV-D68 protease as well as in corresponding antiviral assays. Enteroviruses belong to the *Picornaviridae* and cause a variety of clinical manifestations that include also serious diseases, such as meningitis, viral encephalitis, myopericarditis, acute flaccid myelitis (AFM), hand, or neonatal sepsis^50^. EV-D68 was the primary causative agent in the 2014 outbreak, which was associated with a surge in cases of AFM. Although AFM occurs only in a subset of cases, EV-D68 infections more commonly present as respiratory illness^51^. EV-A71 is the principal causative agent of hand, foot, and mouth disease (HFMD), a self-limiting febrile illness that has caused large-scale outbreaks in the Asia–Pacific region^52^. Coxsackievirus B3 (CVB3), a strain belonging to the EV-B species, is the etiological agent of viral myocarditis and sudden cardiac death, and can be particularly fatal in newborns and young children, where it often manifests with septic-like disease. Given the severity and diversity of diseases caused by these pathogens, the development of broad-spectrum antiviral agents covering enteroviruses would be highly valuable.

The principal feasibility of a re-purposing of coronalviral protease inhibitors towards enteroviruses has been demonstrated before^26,27^. However, while our macrocycles were weaker inhibitors than nirmatrelvir for SARS-CoV-2 M^pro^ and rupintrivir for EV 3C^pro^, the two positive controls did not exert broad-spectrum activity against both proteases. This demonstrates that dual target inhibition is non-trivial. In previous studies, the activities of compounds in the “second” indication was mostly weaker or equal to the one that guided their design. In contrast, we found inhibitors whose enteroviral activity surpassed those against the coronavirus. In particular, **20f** demonstrated the highest potency, exhibiting EC_50_ values of 0.033–0.15 µM against three enteroviruses, thereby highlighting its promise as a broad-spectrum antiviral candidate. The macrocycle **20f** incorporates a phenylalanine residue at the S2 pocket, which we identified as an optimal substituent in earlier studies. The large and open architecture of the 3C^pro^ S2 pocket accommodates this residue effectively, thereby facilitating proper alignment of the inhibitor and enabling covalent interaction with the catalytic cysteine of the Cys–His-Asp triad. In addition, the trifluoroacetyl–*tert*-leucine capping group of **20f** seems to engage in favorable interactions within the 3C^pro^ active site, given its superiority over other P4 residues. A first ADME profiling indicated sufficient metabolic and chemical stability of **20f**. These findings highlight the structural features critical for potent protease inhibition and encourage us to generate further druggable macrocyclic antiviral compounds based on the promising hit **20f**.

## Conclusion

A structure-based design of macrocyclic inhibitors targeting the SARS-CoV-2 main protease was achieved by two different approaches, that were both successful in terms of target binding. However, the inactivity of the nitrile series showcases how subtle design flaws induce drastic functional impairments. In contrast, the endocyclic α-ketoamides bound, were functionally active and exerted anti-coronaviral activity. Notably, **20f** could be “repurposed” to inhibit the related enteroviral 3C proteases and exert potent antiviral activity in the nM range. While most studies aim to achieve broad-spectrum antiviral activity with compounds acting on host targets, the macrocycles prepared here demonstrate that this is also possible with direct-acting antivirals.

## Methods

### General information on chemical synthesis

Commercial reagents were used as received, and all other reagents were prepared using known literature procedures. All solvents used for reactions, workups, and purifications had the HPLC purity grade. Dried solvents were purchased in water-free form (99.5%, extra dry, absolute, AcroSeal^TM^, ACROS Organics^TM^) and used as received. Reactions were either monitored by Liquid Chromatography-coupled Mass Spectrometry (LCMS) analysis or thin-layer chromatography (TLC) on “TLC Silica gel 60 F254” plates (Merck) and visualized by staining with aqueous basic KMnO_4_. LCMS was conducted with an Agilent® 1260 HPLC System with a DAD detector and an Agilent® 6130 quadrupole mass detector with Electrospray Ionization (ESI) (MeCN/H_2_O + 0.1% HCOOH). Flash chromatography was performed on silica gel 60 (technical grade, pore size 60 Å, 40–63 μm, 230–400 mesh, Supelco®). Reverse-Phase High Pressure Liquid Chromatography (RP-HPLC) was performed with a Phenomenex Luna C18 RP-column 00G-4252-P0-AX, 5 μm, 100 Å, 250 × 21.2 mm (flow rate 10 mL/min, max. loading 100 mg crude) coupled to a Thermo Fisher Scientific® Dionex Ultimate 3000 HPLC-System using a ternary solvent mixture (MeCN/H_2_O + 0.1% CF_3_ COOH). Product-containing fractions were identified by LCMS and lyophilized to dryness. Purity of final compounds was measured on SHIMADZU HPLC (Model Number: CBM-20A) with Gemini® 3 μm NX-C18-110 Å, LC-Column 50 × 2 m.m (flow rate 1 mL/min). For Nuclear Magnetic Resonance (NMR) spectroscopic analysis, Bruker Avance III or Bruker Avance III HD spectrometers were employed and ^1^H NMR spectra were recorded at 500 or 700 MHz, respectively 126 and 176 MHz for ^13^C NMR spectra. Chemical shifts are reported in parts per million (ppm) using the residual non deuterated solvent resonance for proton measurements and the solvent resonance for carbon measurements as internal standards (D_3_COD: ^1^H = 3.31 ppm and ^13^C = 49.00 ppm; DMSO-*d*_*6*_: ^1^H = 2.50 ppm and ^13^C = 39.52 ppm, CD_3_CN: ^1^H = 1.94 ppm and ^13^C = 1.32, 118.26 ppm). Data are reported as follows: chemical shift (multiplicity (s = singlet, d = doublet, t = triplet, q = quartet, m = multiplet), coupling constant(s) in Hz, integration). High resolution mass spectrometry (HRMS) was performed using a Dionex Ultimate 3000 HPLC system equipped with a DAD detector and a Bruker maXis HD QTOF mass detector with electrospray ionization (ESI). Samples were injected via an Ultimate 3000RS auto sampler (Thermo Fisher Scientific) and data are reported in the form of mass-to- charge ratio (m/z). All the final compounds used for biological studies are purified based on prep-HPLC and having purity more than 95%. Analytical HPLC-MS traces of several compounds exhibit a second peak with a mass that is +18 Da heavier than the expected mass; this peak is ascribed to a reversible hydrate formation upon the addition of water to the keto group.

### Synthesis of (S)-1-amino-5-(benzyloxy)-1,5-dioxopentan-2-aminium trifluoro acetate

N-Boc-D-Glu(OBn)-OH (3 g, 8.9 mmol) was dissolved in 1,4-dioxane, and then NH_4_HCO_3_ (1.1 g, 14.08 mmol), pyridine (0.9 mL), Boc_2_O (2.7 mL, 12.38 mmol) were added into solution under rt for 16 h. 1,4-Dioxane was removed by rotary evaporation. The residue was dissolved in EtOAc and extracted with water. Organic layer was collected and dried over Na_2_SO_4_, followed by the concentration and purification by flash column chromatography, eluting with 20–30% of ethylacetate/cyclohexane afforded benzyl (S)-5-amino-4-((*tert-*butoxycarbonyl) amino)-5-oxopentanoate a white solid. ^1^H NMR (500 MHz, DMSO-*d*_6_, 298 K): δ 7.42–7.30 (m, 5H), 7.32–7.22 (m, 1H), 7.00 (s, 1H), 6.81 (d, *J* = 8.2 Hz, 1H), 5.17–5.01 (s, 2H), 3.95 (ddd, *J* = 13.9, 7.8 Hz, 1H), 2.45–2.30 (m, 2H), 1.96–1.69 (m, 2H), 1.45–1.28 (m, 9H). ESI-MS (m/z): 337 [M + H]^+^.

To the stirred solution of benzyl (S)-5-amino-4-((*tert*-butoxycarbonyl) amino)-5-oxopentanoate (1.3 g, 3.389 mmol) in CH_2_Cl_2_ (10 mL), Trifluoro acetic acid (4 mL, excess) was added at ice-cold temperature. The reaction mixture was stirred for 6 h. After confirming the completion of the reaction based on LCMS, it was concentrated to dryness and co-evaporated with methanol. This resulting solid was used for further reaction without purification (1.35 g, 100%). ESI-MS Da (m/z): 237 Da [M + H]^+^.

### Synthesis of benzyl (S)-5-amino-4-((S)-2-(3-((tert-butoxycarbonyl) amino)-2-oxopyridin-1(2H)-yl)-3-cyclopropylpropanamido)-5-oxopentanoate (**2**)

To a solution of (S)-2-(3-((*tert*-butoxycarbonyl) amino)-2-oxopyridin-1(2H)-yl)-3-cyclopropylpropanoic acid **1** (750 mg, 2.33 mmol) in DMF (10 mL), HATU (1.8 g, 4.66 mmol) was added at 0 °C. The reaction mixture was stirred at 0 °C under a nitrogen atmosphere, and after few minutes, a pre-stirred solution of (S)-1-amino-5-(benzyloxy)-1,5-dioxopentan-2-aminium trifluoro acetate (730 mg, 3.09 mmol) and triethylamine (1.41 g,13.97 mmol) in DMF (5 mL) at 0 °C was added. The reaction was continued for 6 h at 0 °C. Progress of the reaction was monitored based on TLC and LCMS. The reaction mixture was concentrated, diluted with CH_2_Cl_2_ (50 mL), and washed with water. The resulting organic layer was washed with brine solution and dried over Na_2_SO_4_. The resulting organic was evaporated to dryness and purified on a flash column over a C18 column (25 − 45% CH_3_CN/H_2_O). The product was isolated with a 90% yield (1.13 g). ^1^H NMR (600 MHz, DMSO-*d*_*6*_, 298 K): δ 8.51 (d, *J* = 8.1 Hz, 0.5 H), 8.30 (d, *J* = 8.0 Hz, 0.5 H), 7.85–7.66 (m, 2H), 7.47–7.27 (m, 6H), 7.07 (s, 1H), 6.31–6.21 (m, 1H), 5.53 (t, *J* = 14.9 Hz, 1H), 5.07 (sx2, 2H), 4.26–4.13 (m, 1H), 2.42–2.26 (m, 2H), 2.04–1.92 (m, 2H), 1.81 (d, *J* = 15.0 Hz, 2H), 1.46 (sx2, 9H), 0.48 (t, *J* = 14.8 Hz, 1H), 0.35–0.24 (m, 2H), 0.15–0.06 (m, 1H), −0.02–0.102 (m, 1H). ESI-MS (m/z): 541 Da [M + H]^+^.

### Synthesis of benzyl (S)-5-amino-4-((S)-2-(3-amino-2-oxopyridin-1(2H)-yl)-3-cyclopropylpropanamido)-5-oxopentanoate TFA salt (**3**)

To the stirred solution of (S)-5-amino-4-((S)-2-(3-((*tert*-butoxycarbonyl) amino)-2-oxopyridin-1(2H)-yl)-3-cyclopropylpropanamido)-5-oxopentanoate **2** (650 mg, 1.20 mmol) in CH_2_Cl_2_ (10 mL), Trifluoro acetic acid (2 mL, excess) was added at ice-cold temperature. The reaction mixture was stirred for 6 h. After confirming the completion of the reaction based on LCMS, it was concentrated to dryness and co-evaporated with methanol. This resulting solid of **3** was used for further reaction without purification (663 mg, 100%). ESI-MS (m/z): 441 Da [M + H]^+^.

### Synthesis of benzyl (S)-5-amino-4-((S)-2-(3-(5-(((benzyloxy)carbonyl) amino) pentanamido)-2-oxopyridin-1(2H)-yl)-3-cyclopropylpropanamido)-5-oxopentanoate (**4a**)

Following the procedure described for the preparation of compound **2** with a slight modification of conducting the reaction with **3** (300 mg, 0.541 mmol) and 5-(((benzyloxy) carbonyl) amino) pentanoic acid (252 mg, 1.083 mmol), afforded **4a** (236 mg, 65%) as a white solid. ^1^H NMR (500 MHz, CD_3_CN 298 K) δ: 8.54–8.44 (m, 1H), 8.37–8.27 (m, 1H), 7.56–7.23 (m, 10H), 6.95 (s, 0.5H), 6.70 (s, 0.5H), 6.36–6.22 (m, 1H), 6.01–6.13 (m, 1H), 5.82–5.70 (m, 1H), 5.37 (t, *J* = 7.6 Hz, 0.3H), 5.16 (dd, *J* = 8.4, 6.9 Hz, 0.68H), 5.10–5.01 (m, 4H), 4.35–4.27 (m, 1H), 3.20–3.04 (m, 2H), 2.50–2.25 (m, 4H), 2.22–2.04 (m, 1H), 1.93–1.74 (m, 3H), 1.68–1.52 (m, 2H), 1.51–1.39 (m, 2H), 0.60–0.48 (m, 1H), 0.41–0.27 (m, 2H), 0.11–0.02 (m, 1H), 0.00 to −0.09 (m, 1H). ESI-MS (m/z): 674 Da [M + H]^+^.

### Synthesis of benzyl (S)-5-amino-4-((S)-2-(3-(6-(((benzyloxy)carbonyl) amino) hexanamido)-2-oxopyridin-1(2H)-yl)-3-cyclopropylpropanamido)-5-oxopentanoate (**4b**)

Following the procedure described for the preparation of compound **2** with a slight modification of conducting the reaction with **3** (660 mg, 1.19 mmol) and 6-(((benzyloxy)carbonyl) amino) hexanoic acid (632 mg, 2.385 mmol) afforded **4b** (605 mg, 74%) as a white solid. ^1^H NMR (500 MHz, CD_3_CN, 298 K) δ: 8.50–8.28 (m, 2H), 7.44–7.32 (m, 7H), 7.25–7.00 (m, 2H), 6.81–6.65 (m, 2H), 6.41–6.21 (m, 2H), 5.88–5.56 (m, 2H), 5.22 (ddd, *J* = 15.3, 12.5, 6.6 Hz, 1H), 5.11–4.98 (m, 4H), 4.32–4.16 (m, 1H), 3.19–3.00 (m, 2H), 2.51–2.23 (m, 4H), 2.14–2.00 (m, 2H), 1.92–1.79 (m, 2H), 1.70–1.55 (m, 2H), 1.53–1.42 (m, 2H), 1.39–1.25 (m, 2H), 0.63–0.51 (m, 1H), 0.43–0.28 (m, 2H), 0.16–0.06 (m, 1H), 0.04 to −0.10 (m, 1H). ESI-MS (m/z): 688 Da [M + H]^+^.

### Synthesis of (S)-5-amino-4-((S)-2-(3-(5-aminopentanamido)-2-oxopyridin-1(2H)-yl)-3-cyclopropylpropanamido)-5-oxopentanoic acid (**5a**)

To a solution of **4a** (45 mg, 0.0669 mmol) in methanol (1.5 mL) was added Pd/C (5 mg, 10% on carbon) under nitrogen. The suspension was degassed under vacuum and purged with hydrogen several times. The mixture was stirred hydrogen atmosphere at 20 °C for 5 h. LCMS showed the starting material was consumed completely. The mixture was filtered, the solid was washed with methanol (5 mLX3). The combined filtrate was concentrated in vacuum to give **5a** (24 mg, 80%) as white solid, which was used for the next step without further purification. ^1^H NMR (600 MHz, CD_3_OD, 298 K) δ: 8.57–8.16 (m, 1H), 7.55–7.40 (m, 1H), 7.40–7.27 (m, 1H), 6.38 (dt, *J* = 11.5, 7.2 Hz, 1H), 5.48–5.31 (m, 1H), 4.37–4.23 (m, 1H), 3.05–2.90 (m, 2H), 2.82–2.43 (m, 3H), 2.37–1.90 (m, 8H), 1.83–1.53 (m, 6H), 0.62 (dt, *J* = 12.1, 7.3 Hz, 1H), 0.52–0.35 (m, 2H), 0.20–0.13 (m, 1H), 0.08–0.01 (m, 1H). ESI-MS (m/z): 450 Da [M + H]^+^.

### Synthesis of cyclic amide (**6a**)

A solution of **5a** (50 mg, 0.111 mmol) in DMF (20 mL), HATU (64 mg, 0.167 mmol) and TEA (33.8 mg, 0.335 mmol) was added at ice cold temp. Reaction was stirred at ice cold temp under dry conditions. Reaction was continued for 5 h in the same temp. Progress of the reaction was monitored based on TLC and LCMS. After 5 h, the reaction was stopped and neutralized with 10% TFA in acetonitrile. Resulted crude was purified on prep-HPLC to give the **6a** as separated diastereomers (16 + 20 mg, 75%) as white solid. Dia-1: ^1^H NMR (700 MHz, CD_3_OD, 298 K) δ: 8.19 (d, *J* = 7.1 Hz, 1H), 7.40 (t, *J* = 9.3 Hz, 1H), 6.42 (t, *J* = 7.2 Hz, 1H), 5.14–5.05 (m, 1H), 4.37 (dd, *J* = 9.9, 3.1 Hz, 1H), 2.97–2.86 (m, 1H), 2.56–2.24 (m, 4H), 2.24–2.15 (m, 1H), 2.14–1.97 (m, 3H), 1.90–1.41 (m, 6H), 1.37–1.24 (m, 1H), 0.71–0.61 (m, 1H), 0.50–0.40 (m, 2H), 0.16 (d, *J* = 8.7 Hz, 1H), 0.07 (t, *J* = 12.7 Hz, 1H). ESI-MS (m/z): 432 Da [M + H]^+^.

Dia-2: ^1^H NMR (700 MHz, CD_3_OD, 298 K) δ: 8.36–8.28 (m, 1H), 7.45 (d, *J* = 5.9 Hz, 1H), 6.45–6.41 (m, 1H), 4.84–4.78 (m, 1H), 4.34–4.26 (m, 1H), 3.37–3.33 (m, 1H), 3.24–3.17 (m, 1H), 2.86–2.79 (m, 1H), 2.61–2.53 (m, 1H), 2.43–2.41 (m, 1H), 2.38–2.23 (m, 3H), 2.07–2.13 (m, 1H), 2.07–1.98 (m, 2H), 1.88–1.79 (m, 1H), 1.77–1.69 (m, 1H), 1.64–1.51 (m, 2H), 0.59–0.51 (m, 1H), 0.42 (ddd, *J* = 9.6, 7.4, 5.0 Hz, 1H), 0.39–0.31 (m, 1H), 0.15–0.09 (m, 1H), −0.04 to −0.10 (m, 1H). ESI-MS (m/z): 432 Da [M + H]^+^.

### Synthesis of benzyl (S)-4-((S)-2-(3-(5-(((benzyloxy)carbonyl) amino) pentanamido)-2-oxopyridin-1(2H)-yl)-3-cyclopropylpropanamido)-4-cyanobutanoate (**7a**)

Compound **4a** (180 mg, 0.282 mmol), inner salt (Burgess Reagent; 135 mg, 0.567 mmol) was added to a solution in CH_2_Cl_2_ (5 mL). After the reaction mixture had been stirred at 25 °C for 4 h, the reaction mixture was diluted and quenched by saturated aqueous sodium bicarbonate solution (2 mL). The separated organic phase was concentrated and purified on reverse phase flash system flash column over C18 column (30–50% H_2_O/ACN), which gave **7a** (140 mg, 75%) of as a white solid. ^1^H NMR (700 MHz, DMSO-*d*_*6*_, 298 K) δ : 9.18–8.99 (m, 2H), 8.22 (t, *J* = 5.8 Hz, 1H), 7.45–7.18 (m, 11H), 6.29 (dt, *J* = 12.6, 7.2 Hz, 1H), 5.57–5.43 (m, 1H), 5.14–4.96 (sX2, 4H), 4.88–4.76 (m, 1H), 3.01–2.96 (m, 2H), 2.48–2.35 (m, 4H), 2.14–1.95 (m, 3H), 1.81–1.70 (m, 1H), 1.63–1.35 (m, 4H), 0.55–0.43 (m, 1H), 0.39–0.26 (m, 2H), 0.10 (dd, *J* = 9.2, 4.3 Hz, 1H), 0.02 (dd, *J* = 13.3, 11.3 Hz, 1H). ESI-MS (m/z): 656 Da [M + H]^+^.

### Synthesis of benzyl (S)-4-((S)-2-(3-(6-(((benzyloxy) carbonyl) amino) hexanamido)-2-oxopyridin-1(2H)-yl)-3-cyclopropylpropanamido)-4-cyanobutanoate (**7b**)

Following the procedure described for the preparation of compound **7a** with a slight modification of conducting the reaction with **4b** (605 mg, 0.88 mmol) and inner salt (Burgess Reagent; 419 mg, 1.76 mmol) afforded **7b** (302 mg, 51%) as a white solid. ^1^H NMR (500 MHz, CD_3_CN, 298 K) δ: 8.43*–*8.28 (m, 2H), 7.43–7.27 (m, 10H), 7.23–7.18 (m, 1H), 6.34–6.21 (m, 1H), 5.63 (s, 1H), 5.45–5.26 (m, 1H), 5.14–4.98 (m, 4H), 4.86–4.69 (m, 1H), 3.16–2.95 (m, 2H), 2.54–2.30 (m, 4H), 2.15–2.01 (m, 2H), 1.92–1.87 (m, 2H), 1.61 (dq, *J* = 14.2, 7.3 Hz, 2H), 1.51–1.41 (m, 2H), 1.39–1.23 (m, 2H), 0.61–0.49 (m, 1H), 0.42–0.30 (m, 2H), 0.12–0.04 (m, 1H), 0.03 to −0.06 (m, 1H), ESI-MS (m/z): 670 Da [M + H]^+^.

### Synthesis of (S)-4-((S)-2-(3-(5-aminopentanamido)-2-oxopyridin-1(2H)-yl)-3-cyclopropylpropanamido)-4-cyanobutanoic acid (**8a**)

Following the procedure described for the preparation of **5a** with a slight modification of conducting the reaction with **7a** (130 mg, 0.198 mmol) and Pd/C (15 mg, 10% on charcoal) afforded **8a** (70 mg, 82%) as a white solid which was used for further reaction without purification. ESI-MS (m/z): 432 Da [M + H]^+^.

### Synthesis of (S)-4-((S)-2-(3-(6-aminohexanamido)-2-oxopyridin-1(2H)-yl)-3-cyclopropylpropanamido)-4-cyanobutanoic acid (**8b**)

Following the procedure described for the preparation of **5a** with a slight modification of conducting the reaction with **7b** (300 mg, 0.4484 mmol) and Pd/C (32 mg, 10% on charcoal), afforded **8b** (170 mg, 85%) as a white solid. ^1^H NMR (500 MHz, CD_3_OD, 298 K) δ : 8.34–8.24 (m, 1H), 7.53–7.42 (m, 1H), 6.38 (tt, *J* = 9.3, 7.3 Hz, 1H), 5.47–5.36 (dt, *J* = 17.3, 8.6 Hz, m, 1H), 4.85 (dd, *J* = 8.5, 6.1 Hz, 1H), 4.79 (t, *J* = 7.3 Hz, 1H), 2.96–2.87 (m, 2H), 2.56–2.43 (m, 2H), 2.38–2.26 (m, 2H), 2.17–1.89 (m, 4H), 1.78–1.63 (m, 4H), 1.52–1.39 (m, 2H), 0.70–0.57 (m, 1H), 0.50–0.39 (m, 2H), 0.22–0.13 (m, 1H), 0.11–0.03 (m, 1H). ESI-MS (m/z): 446 Da [M + H]^+^.

### Synthesis of macrocyclic nitrile (**9a**)

Following the procedure described for the preparation of compound **6a** with a slight modification of conducting the reaction with compound **8a** (70 mg, 0.162 mmol) and HATU (95 mg, 0.2517 mmol), afforded compound **9a** as separated diastereomers (12 + 17.8 mg, 43%) as a white solid.

### (2 R,5S)-2-(cyclopropylmethyl)-3,8,14,20-tetraoxo-1,4,9,15-tetraazabicyclo[14.3.1]icosa-16,18-diene-5-carbonitrile (**9a-H**)

HPLC purity: 95.5%,^1^H NMR (500 MHz, CD_3_OD, 298 K): δ 8.17 (dd, *J* = 7.3, 1.3 Hz, 1H), 7.40 (dd, *J* = 7.0, 1.3 Hz, 1H), 6.43–6.30 (m, 1H), 4.99 (dd, *J* = 9.7, 5.8 Hz, 1H), 4.65 (dd, *J* = 10.5, 3.5 Hz, 1H), 3.15–3.05 (m, 1H), 2.98 (s, 1H), 2.60–2.35 (m, 3H), 2.32–2.16 (m, 2H), 2.11–1.97 (m, 2H), 1.92–1.69 (m, 2H), 1.60–1.43 (m, 2H), 0.68–0.39 (m, 2H), 0.45 (tdd, *J* = 13.3, 9.0, 5.1 Hz, 2H), 0.17 (dt, *J* = 8.8, 4.9 Hz, 1H), 0.09–0.01 (m, 1H). ^13^C NMR (176 MHz, CD_3_OD, 298 K) δ: 174.91, 172.09, 159.69, 132.67, 126.33, 119.37, 108.03, 66.20, 57.86, 41.68, 39.70, 34.16, 31.75, 29.07, 28.38, 24.41, 17.65, 8.76, 5.37, 4.54, 0.10. HRMS: calcd. for C_21_H_28_N_5_O_4_ [M + H]^+^, m/z = 414.2141 Da; found, m/z = 414.2136 Da.

### (2S,5S)-2-(cyclopropylmethyl)-3,8,14,20-tetraoxo-1,4,9,15-tetraazabicyclo[14.3.1]icosa-16,18-diene-5-carbonitrile (**9a-K**)

HPLC purity: 96.5%, ^1^H NMR (500 MHz, CD_3_OD, 298 K) δ: 8.18 (dt, *J* = 8.7, 4.4 Hz, 1H), 7.41 (dd, *J* = 7.0, 1.4 Hz, 1H), 6.43–6.39 (m, 1H), 4.98–4.85 (m, 2H), 3.38–3.32 (m, 1H), 2.92 (ddd, *J* = 13.4, 11.0, 7.1 Hz, 1H), 2.54–2.33 (m, 4H), 2.27–1.94 (m, 4H), 1.85–1.69 (m, 2H), 1.59–1.41 (m, 2H), 0.70–0.60 (m, 1H), 0.53–0.38 (m, 2H), 0.28–0.12 (m, 1H), 0.06–0.01 (m, 1H). ^13^C NMR (126 MHz, CD_3_OD, 298 K) δ: 173.21, 172.71, 169.93, 157.74, 128.27, 124.78, 118.15, 106.22, 64.14, 56.23, 37.95, 36.67, 32.64, 29.94, 27.64, 23.14, 16.03, 7.02, 3.67, 3.20, −1.45. ESI-HRMS: calcd. for C_21_H_28_N_5_O_4_ [M + H]^+^, m/z = 414.2141 Da; found, m/z = 414.2138 Da.

### Synthesis of macrocyclic nitrile (**9b**)

Following the procedure described for the preparation of **6a** with a slight modification of conducting the reaction with **8b** (85 mg, 0.191 mmol) and HATU (108 mg, 0.286 mmol) afforded **9b** as separated diastereomers (24 + 28 mg, respectively, 64% in sum) as a white solid.

### (2R,5S)-2-(cyclopropylmethyl)-3,8,15,21-tetraoxo-1,4,9,16-tetraazabicyclo[15.3.1]henicosa-17,19-diene-5-carbonitrile (**9b-H**):

^1^H NMR (500 MHz, CD_3_OD, 298 K): δ 8.17 (dd, *J* = 7.4, 1.7 Hz, 1H), 7.44 (dd, *J* = 7.0, 1.7 Hz, 1H), 6.45–6.35 (m, 1H), 4.99 (dd, *J* = 9.7, 5.8 Hz, 1H), 4.65 (dd, *J* = 10.2, 4.0 Hz, 1H), 3.08 (ddd, *J* = 13.5, 6.9, 3.8 Hz, 1H), 2.96 (d, *J* = 5.9 Hz, 1H), 2.52–2.35 (m, 3H), 2.30–2.19 (m, 2H), 2.14–2.04 (m, 1H), 2.02–1.94 (m, 1H), 1.73 (qd, *J* = 13.9, 7.1 Hz, 2H), 1.57–1.37 (m, 4H), 0.67–0.55 (m, 1H), 0.51–0.36 (m, 2H), 0.17 (dt, *J* = 9.3, 5.0 Hz, 1H), 0.07 to −0.00 (m, 1H). ^13^C NMR (176 MHz, CD_3_OD, 298 K): δ 174.00, 173.07, 170.01, 158.15, 132.14, 128.28, 126.24, 117.78, 106.09, 65.16, 57.78, 39.72, 38.30, 36.53, 32.22, 30.64, 27.96, 25.18, 16.08, 7.15, 3.76, 2.90, −1.46. HRMS: calcd. for C_22_H_30_N_5_O_4_ [M + H]^+^, m/z = 428.2297 Da; found, m/z = 428.2298 Da.

### (2S,5S)-2-(cyclopropylmethyl)-3,8,15,21-tetraoxo-1,4,9,16-tetraazabicyclo[15.3.1]henicosa-17,19-diene-5-carbonitrile (**9b-K**)

HPLC purity: 97.6%, ^1^H NMR (500 MHz, CD_3_OD, 298 K): δ 8.21 (dd, *J* = 7.4, 1.7 Hz, 1H), 7.45 (dd, *J* = 7.1, 1.7 Hz, 1H), 6.39 (t, *J* = 7.2 Hz, 1H), 5.06 (t, *J* = 7.7 Hz, 1H), 4.97 (dd, *J* = 9.9, 4.7 Hz, 1H), 3.49–3.40 (m, 1H), 3.01–2.90 (m, 2H), 2.53–2.30 (m, 3H), 2.22–2.04 (m, 3H), 1.97–1.63 (m, 4H), 1.61–1.27 (m, 5H), 0.75–0.63 (m, 1H), 0.57–0.40 (m, 2H), 0.22–0.06 (m, 2H). ^13^C NMR (176 MHz, MeOD, 298 K) δ: 173.74, 172.82, 170.13, 157.89, 130.95, 128.45, 125.69, 118.11, 105.91, 62.89, 56.55, 55.82, 38.71, 36.17, 33.24, 30.53, 28.82, 27.91, 24.92, 16.08, 15.64, 15.54, 7.09, 3.59, -1.49. HRMS: calcd. for C_22_H_30_N_5_O_4_ [M + H]^+^, m/z = 428.2297 Da; found, m/z = 428.2297 Da.

### Synthesis of methyl (S)-3-(3-allylphenyl)-2-((tert-butoxycarbonyl) amino) propanoate (**10**)

A two-neck round-bottom flask was charged (S)-3-(3-bromophenyl)-2-((*tert*-butoxycarbonyl) amino) propanoate (1.14 g, 3.19 mmol), CsF (1.93 g, 12.69 mmol), in THF (50 mL), de-gassed with argon gas for 15 min. 2-allyl-4,4,5,5-tetramethyl-1,3,2-dioxaborolane **2a** (1.61 g, 8.39 mmol) and Pd(PPh_3_)_4_ (0.3 g, 0.259 mmol) were added and the resulting reaction mixture was heated to reflux for 24 h. At the end of the reaction (TLC monitoring), crude was dissolved in 100 ml of ethyl acetate and washed with water. Aqueous layer was extracted ethyl acetate (2 × 50 ml) and combined organic layer was washed with brine and dried over Na_2_SO_4_. Resulted solution was evaporated to dryness and purified over column chromatography with 1:9 mixture EA/Cyclohexane on silica gel, which gave methyl (S)-3-(3-allylphenyl)-2-((*tert*-butoxycarbonyl) amino) propanoate (**10**). Yield: 2.7 g, 93%. ^1^H NMR (700 MHz, DMSO-*d*_*6*_, 298 K): δ 7.31–7.18 (m, 2H), 7.10–7.01 (m, 3H), 5.97–5.90 (m, 1H), 5.09–5.03 (m, 2H), 4.18–4.09 (m, 1H), 3.65–3.55 (m, 3H), 3.33 (s, 2H), 2.98–2.90 (m, 1H), 2.88–2.80 (m, 1H), 1.37–1.28 (s, 9H). ESI-MS (m/z): 320 Da [M + H]^+^.

### Synthesis of (S)-3-(3-allylphenyl)-2-((tert-butoxycarbonyl) amino) propanoic acid (**11**)

A solution of methyl (S)-3-(3-allylphenyl)-2-((*tert*-butoxycarbonyl) amino) propanoate (**10**) (1.1 g, 3.34 mmol) in methanol (24 mL) and water (6 mL) was added to LiOH.H_2_O (0.29 g, 6.95 mmol). The reaction mixture was stirred for 2 h at 25 °C. The reaction mixture pH was adjusted to 4–5 with saturated aqueous citric acid solution and concentrated minimum solution. Resulted crude was re-dissolved in CH_2_Cl_2_ and washed with water and brine solution. Resulted solution was evaporated to dryness which gave (S)-3-(3-allyl phenyl)-2-((*tert*-butoxy carbonyl) amino) propanoic acid (**11**) as a white solid. Yield: 841 mg, 80%. ^1^H NMR (300 MHz, DMSO-*d*_*6*_, 298 K): δ 7.20 (t, *J* = 12.4 Hz, 1H), 7.13–6.83 (m, 4H), 6.04–5.86 (m, 1H), 5.15–4.99 (m, 2H), 4.01 (dd, *J* = 16.9, 6.0 Hz, 1H), 3.38–3.27 (m, 2H), 3.01 (dd, *J* = 18.1, 8.8 Hz, 1H), 2.87–2.69 (m, 1H), 1.32 (s, 9H). ESI-MS (m/z): 306 Da [M + H]^+^. Crude was sufficient pure to perform further transformation. We have repeated the reaction in larger gram scale reactions.

### Synthesis of methyl (S)-2-((S)-3-(3-allylphenyl)-2-((tert-butoxycarbonyl) amino) propanamido)-3-((S)-2-oxopyrrolidin-3-yl) propanoate (**12**)

To a solution of **11** (1.43 g, 4.68 mmol) in DMF (30 mL) was added Et_3_N (3.83 mL, 27.5 mmol) and HATU (3.5 g, 9.2 mmol). The mixture was stirred at 25 °C for 5 min. Then the (*S*)-methyl 2-amino-3-((*S*)-2-oxopyrrolidin-3-yl) propanoate (1.56 g, 7.0 mmol) was added to the above mixture. Progress of the reaction was monitored based on TLC and LCMS. After completion of reaction, the mixture was added to ice-cold water and extracted in CH_2_Cl_2_ (3 × 50 mL). Resulted organic layer was washed with brine solution and dried over Na_2_SO_4_. Resulted organic was evaporated to dryness and purified on Flash Chromatography on C18 column (25–45% of H_2_O/ACN). Product **12** was isolated with yield: 1.61 g, 74%. ^1^H NMR (700 MHz, CD_3_CN, 298 K): δ 7.91–7.70 (m, 1H), 7.25–7.17 (m, 1H), 7.12–6.81 (m, 3H), 6.15–6.05 (m, 1H), 6.0–5.93 (m, 1H), 5.50–5.38 (m, 1H), 5.12–5.00 (m, 2H), 4.41–4.07 (m, 3H), 3.68 (sx2, 3H), 3.36 (s, 2H), 3.25–3.13 (m, 2H), 3.07–2.95 (m, 1H), 2.88–2.53 (m, 2H), 2.36–2.16 (m, 2H), 2.09–1.98 (m, 1H), 1.87–1.65 (m, 3H), 1.35 (sx2, 9H). ESI-MS (m/z): 474 Da [M + H]^+^.

### Synthesis of tert-butyl ((S)-3-(3-allylphenyl)-1-(((S)-1-hydroxy-3-((S)-2-oxopyrrolidin-3-yl) propan-2-yl) amino)-1-oxopropan-2-yl) carbamate (**13**)

The compound **12** (1.28 g, 2.71 mmol) and NaBH_4_ (0.844 g, 21.6 mmol) were dissolved in methanol (50 mL) and the reaction mixture was stirred at 20 °C for 7 h. The mixture was treated with 10% ammonium chloride solution and on concentration to give as residue, then extracted with DCM (100 mL × 3). The organic phase was dried with Na_2_SO_4_ and concentrated. The residue was purified on a silica gel column to give **13**. Yield: 1.2 g, 100%, which was sufficient pure to perform further transformation. ESI-MS (m/z): 446 Da [M + H]^+^.

### Synthesis of tert-butyl ((S)-3-(3-allylphenyl)-1-oxo-1-(((S)-1-oxo-3-((S)-2-oxopyrrolidin-3-yl) propan-2-yl) amino) propan-2-yl)carbamate (**14**)

To a solution of **13** (1 g, 2.25 mmol) in DCM (25 mL) was added DMP (1.23 g, 2.9 mmol). The reaction mixture was stirred for 3 h at 25 °C. The reaction mixture was concentrated to give a residue. It was purified on a silica gel column to give **14**. Yield: 633 mg, 65%. ^1^H NMR (700 MHz, DMSO-*d*_*6*_, 298 K): δ 9.30 (s, 1H), 8.48–8.28 (m, 1H), 8.09–7.84 (m, 1H), 7.68–7.38 (m, 2H), 7.20–6.83 (m, 3H), 6.06–5.88 (m, 1H), 5.68 (s, 1H), 5.14–5.0 (m, 1H), 4.75–4.48 (m, 1H), 4.24–4.05 (m, 1H), 3.77–3.61 (m, 0.5H), 3.33–3.25 (m, 1H), 2.86–2.65 (m, 1H), 2.29 – 1.76 (m, 3H), 1.64–1.45 (m, 1H), 1.42–1.18 (m, 10H). ESI-MS (m/z): 444 Da [M + H]^+^.

### (6S,9S)-6-(3-allylbenzyl)-2,2-dimethyl-4,7,11-trioxo-9-(((S)-2-oxopyrrolidin-3-yl) methyl)-3-oxa-5,8,12-triazaheptadec-16-en-10-yl acetate (**15a**)

To a solution of **14** (200 mg, 0.451 mmol) in DCM (20 mL) was added AcOH (52 μL, 0.916 mmol). Then the mixture was stirred at 25 °C for 10 min. Then 5-isocyanopent-1-ene (88 mg, 0.902 mmol) was added. The reaction was stirred for 12 h. The mixture was purified on a silica gel column to give the acetoxy amide **15a** as mixture of diastereomers (148 mg, 55%) as a white solid.

Dia-1: ^1^H NMR (500 MHz, DMSO-*d*_*6*_, 298 K): δ 8.35–7.81 (m, 2H), 7.79–7.51 (m, 1H), 7.28–6.80 (m, 5H), 6.49–6.15 (m, 1H), 6.03–5.70 (m, 2H), 5.11–4.85 (m, 4H), 4.70 (dx2, *J* = 6.6 Hz, 1H), 4.20–4.04 (m, 1H), 3.98–3.64 (m, 1H), 3.22–2.98 (m, 4H), 2.91–2.69 (m, 1H), 2.28–1.72 (m, 8H), 1.59–1.39 (m, 4H), 1.41–1.13 (m, 9H), ESI-MS (m/z): 599 Da [M + H]^+^.

Dia-2: ^1^H NMR (500 MHz, DMSO-*d*_*6*_, 298 K): δ 8.14–7.81 (m, 2H), 7.63–7.49 (m, 9.2 Hz, 1H), 7.25–6.86 (m, 5H), 6.51–6.22 (m, 1H), 5.95 (ddt, *J* = 16.8, 10.1, 6.7 Hz, 1H), 5.86–5.73 (m, 1H), 5.14–4.80 (m, 4H), 4.37–4.25 (m, 1H), 4.21–4.04 (m, 1H), 3.25–2.97 (m, 4H), 2.94–2.58 (m, 2H), 2.40–1.79 (m, 8H), 1.66–1.39 (m, 3H), 1.37–0.97 (m, 10H). ESI-MS (m/z): 599 Da [M + H]^+^.

### (6S,9S)-6-(3-allylbenzyl)-2,2-dimethyl-4,7,11-trioxo-9-(((S)-2-oxopyrrolidin-3-yl) methyl)-3-oxa-5,8,12-triazaoctadec-17-en-10-yl acetate (**15b**)

Following the procedure described for the preparation of **15a** with a slight modification of conducting the reaction with acetic acid (81 mg, 1.22 mmol) and 7-isocyanohept-1-ene (156 mg, 1.22 mmol) at rt afforded the acetoxy amide **15b** (290 mg, 57%) as a white solid.^1^H NMR (700 MHz, DMSO-*d*_*6*_, 298 K): δ 8.06*–*7.74 (m, 2H), 7.57 (t, *J* = 32.7 Hz, 1H), 7.23–6.83 (m, 6H), 5.99–5.89 (m, 1H), 5.86–5.68 (m, 1H), 5.12–4.76 (m, 4H), 4.36–4.17 (m, 2H), 3.20–2.96 (m, 5H), 2.93–2.55 (m, 3H), 2.28–2.05 (m, 5H), 1.98–1.80 (m, 5H), 1.68–1.52 (m, 2H), 1.47–1.14 (m, 15H). ESI-MS (m/z): 627 Da [M + H]^+^.

### (3S,6S,E/Z)-3-((tert-butoxycarbonyl)amino)-4,8-dioxo-6-(((S)-2-oxopyrrolidin-3-yl)methyl)-5,9-diaza-1(1,3)-benzenacyclopentadecaphan-13-en-7-yl acetate (**16b**)

Hoveyda–Grubbs IInd generation catalyst (20.3 mg, 0.0246 mmol) was added to a deoxygenated and magnetically stirred solution of **15a** (123 mg, 0.205 mmol) in DCE (100 mL) maintained under a nitrogen atmosphere. The resulting solution was heated at reflux for 18 h, then cooled and concentrated under reduced pressure. The residue so-obtained was subjected to flash chromatography to give the acetoxy amides **16b,** **16a** (71 mg, 12 mg, in total 71%) as mixture of diastereomers.^1^H NMR (700 MHz, DMSO-*d*_*6*_, 298 K): Major Dia-1: δ 7.96–7.79 (m, 1H), 7.59–7.32 (m, 2H), 7.17 (h, *J* = 7.5 Hz, 1H), 7.11–6.95 (m, 4H), 6.93–6.85 (m, 2H), 5.54–5.41 (m, 1H), 4.81 (d, *J* = 9.9 Hz, 1H), 4.11–4.06 (m, 1H), 3.91 (s, 1H), 3.31–3.18 (m, 2H), 3.17–2.94 (m, 4H), 2.91–2.74 (m, 1H), 2.56–2.51 (m, 3H), 2.19–1.91 (m, 7H), 1.72–1.45 (m, 3H), 1.42–1.32 (s, 9H), 1.30–1.20 (m, 2H), ESI-MS (m/z): 571 Da [M + H]^+^.

Major Dia-2: δ 7.66–7.51 (m, 3H), 7.16–6.97 (m, 4H), 6.60 (d, *J* = 7.6 Hz, 1H), 5.57–5.42 (m, 2H), 4.91 (d, *J* = 2.5 Hz, 1H), 4.27–4.21 (m, 1H), 4.11 (dd, *J* = 13.3, 5.8 Hz, 1H), 3.31–3.23 (m, 2H), 3.21–3.11 (m, 2H), 3.07–3.01 (m, 1H), 2.94–2.89 (m, 2H), 2.78–2.69 (m, 1H), 2.56–2.51 (m, 3H), 2.29–2.11 (m, 3H), 2.11–1.90 (m, 2H), 1.69–1.51 (m, 2H), 1.45–1.32 (m, 10H), 1.32–1.18 (m, 2H). ESI-MS (m/z): 571 Da [M + H]^+^.

### (3S,6S,E/Z)-3-((tert-butoxycarbonyl)amino)-4,8-dioxo-6-(((S)-2-oxopyrrolidin-3-yl)methyl)-5,9-diaza-1(1,3)-benzenacyclotetradecaphan-12-en-7-yl acetate (**16a**)

^1^H NMR (700 MHz, DMSO-*d*_*6*_, 298 K): δ 7.97–7.78 (m, 1H), 7.57–7.41 (m, 1H), 7.20–6.91 (m, 4H), 6.83–6.68 (m, 1H), 5.70–5.60 (m, 1H), 5.45–5.35 (m, 1H), 4.50–4.43 (m, 1H), 4.34–4.25 (m, 1H), 3.94–3.85 (m, 1H), 3.28–2.94 (m, 5H), 2.94–2.64 (m, 2H), 2.34–2.09 (m, 5H), 1.66–1.05 (m, 11H). ESI-MS (m/z): 557 Da [M + H]^+^.

### (3S,6S,E)-3-((tert-butoxycarbonyl)amino)-4,8-dioxo-6-(((S)-2-oxopyrrolidin-3-yl)methyl)-5,9-diaza-1(1,3)-benzenacycloheptadecaphan-15-en-7-yl acetate (**16d**)

Following the procedure described for the preparation of **16b** with a slight modification of conducting the reaction with Hoveyda–Grubbs IInd generation catalyst (16.1 mg, 0.01956 mmol) at 80 °C afforded the acetoxy amide **16d,** **16c** (35 mg, 7 mg, in total 43%) as a white solid.^1^H NMR (700 MHz, DMSO-*d*_*6*_, 298 K): δ 7.86–7.75 (m, 1H), 7.63–7.52 (m, 1H), 7.19–7.08 (m, 2H), 7.06–6.93 (m, 2H), 5.63–5.57 (m, 1H), 5.51–5.38 (m, 1H), 4.86–4.66 (m, 1H), 4.18–4.11 (m, 1H), 3.94 (t, *J* = 15.8 Hz, 1H), 3.38–3.27 (m, 2H), 3.18–2.95 (m, 2H), 2.90–2.74 (m, 2H), 2.66–2.54 (m, 1H), 2.29–2.01 (m, 5H), 1.87–1.80 (m, 5H), 1.62–1.16 (m, 12H). ESI-MS (m/z): 599 Da [M + H]^+^.

### (3S,6S,E)-3-((tert-butoxycarbonyl) amino)-4,8-dioxo-6-(((S)-2-oxopyrrolidin-3-yl)methyl)-5,9-diaza-1(1,3)-benzenacyclohexadecaphan-14-en-7-yl acetate (**16c**)

^1^H NMR (500 MHz, DMSO-*d*_*6*_, 298 K): δ 7.95 (s, 1H), 7.70–7.52 (m, 2H), 7.26–6.90 (m, 3H), 6.49 (s, 1H), 6.36 (s, 1H), 6.29–6.12 (m, 1H), 4.70 (s, 1H), 4.38–4.04 (m, 2H), 3.2 (d, *J* = 10 Hz, 2H), 3.18–2.95 (m, 2H), 2.83–2.62 (m, 2H), 2.38–1.79 (m, 7H), 1.60–1.12 (m, 11H), 0.95–0.78 (m, *2*H). ESI-MS (m/z): 585 Da [M + H]^+^.

### (3S,6S)-3-((tert-butoxycarbonyl) amino)-4,8-dioxo-6-(((S)-2-oxopyrrolidin-3-yl) methyl)-5,9-diaza-1(1,3)-benzenacyclotetradecaphane-7-yl acetate (**17a**)

To a mixture of **16a** (50 mg, 0.089 mmol) in MeOH (5 mL) was added Pd/C (5 mg, 10% on carbon). The mixture was stirred under a hydrogen atmosphere at room temperature until the starting material was consumed completely. The mixture was filtered through highflow and the filtrate was concentrated to give the crude product **17a** (30 mg, 60%) as a white solid. ^1^H NMR (600 MHz, DMSO-*d*_*6*_, 298 K): δ 8.10–7.92 (m, 1H), 7.63–7.47 (m, 2H), 7.42–7.27 (m, 2H), 7.20–6.85 (m, 4H), 6.75–6.51 (m, 2H), 4.79–4.61 (m, 2H), 4.23–4.10 (m, 2H), 3.99–3.87 (m, 2H), 3.15–3.29 (m, 2H), 3.20–2.87 (m, 3H), 2.79–2.51 (m, 3H), 2.26–1.69 (m, 5H), 1.68–1.51 (m, 3H), 1.46–1.05 (m, 10H). ESI-MS (m/z): 559 Da [M + H]^+^.

### (3S,6S)-3-((tert-butoxycarbonyl) amino)-4,8-dioxo-6-(((S)-2-oxopyrrolidin-3-yl) methyl)-5,9-diaza-1(1,3)-benzenacyclopentadecaphane-7-yl acetate (**17b**)

Following the procedure described for the preparation of **17a** with a slight modification of conducting the reaction with Pd/C (12 mg, 10% on carbon) in CH_3_OH at rt afforded the acetoxy amide **17b** (82 mg, 82%) as a white solid.^1^H NMR (700 MHz, DMSO-*d*_*6*_, 298 K): δ 7.87 (s, 1H), 7.56 (s, 1H), 7.30–7.18 (m, 2H), 7.16–7.07 (m, 3H), 7.03–6.93 (m, 2H), 4.80 (t, *J* = 16.5 Hz, 1H), 4.15–4.02 (m, 2H), 3.29 (s, 2H), 3.19–2.97 (m, 4H), 2.84 (d, *J* = 19.0 Hz, 2H), 2.55–2.51 (m, 2H), 2.19–1.87 (m, 5H), 1.74–1.45 (m, 4H), 1.45–0.99 (m, 12H). ESI-MS (m/z): 573 Da [M + H]^+^.

### (3S,6S)-3-((tert-butoxycarbonyl) amino)-4,8-dioxo-6-(((S)-2-oxopyrrolidin-3-yl) methyl)-5,9-diaza-1(1,3)-benzenacyclohexadecaphane-7-yl acetate (**17c**)

Following the procedure described for the preparation of **17a**, with a slight modification in which the reaction was conducted with Pd/C (6 mg, 10% on carbon) in CH₃OH at room temperature, acetoxy amide **17c** (12 mg, 85%) was obtained as a white solid. The product was used for further reactions without purification. ESI-MS (m/z): 587 [M + H]⁺.

### (3S,6S)-3-((tert-butoxycarbonyl) amino)-4,8-dioxo-6-(((S)-2-oxopyrrolidin-3-yl) methyl)-5,9-diaza-1(1,3)-benzenacycloheptadecaphane-7-yl acetate (**17d**)

Following the procedure described for the preparation of **17a** with a slight modification of conducting the reaction with Pd/C (8 mg, 10% on carbon) in CH_3_OH at rt, afforded the acetoxy amide **17d** (23 mg, 87%) as a white solid. ^1^H NMR (600 MHz, DMSO-*d*_*6*_, 298 K): δ 7.99–7.75 (m, 2H), 7.73–7.48 (m, 2H), 7.17–6.97 (m, 4H), 6.85 (t, *J* = 13.0 Hz, 1H), 5.00–4.87 (m, 1H), 4.78 (d, *J* = 5.4 Hz, 1H), 4.19–4.27 (m, 1H), 4.17–3.86 (m, 2H), 3.29–3.15 (m, 1H), 3.16–2.69 (m, 5H), 2.62–2.55 (m, 1H), 2.29–2.10 (m, 2H), 2.05 (d, *J* = 8.9 Hz, 4H), 1.97–1.67 (m, 2H), 1.66–1.37 (m, 6H), 1.37 (sx2, m, 9H), 1.27–1.06 (m, 6H). ESI-MS (m/z): 601 Da [M + H]^+^.

#### (3S,6S)-3-amino-4,8-dioxo-6-(((S)-2-oxopyrrolidin-3-yl) methyl)-5,9-diaza-1(1,3)-benzenacyclotetradecaphane-7-yl acetate hydrochloride (**18a**)

To a solution of the compound **17a** (30 mg, 0.053 mmol) in CH_2_Cl_2_ (3 mL), cooled to 0 °C was added TFA (0.2 ml, excess). After the consumption of the starting material based on LCMS, the solvent was removed under vacuum, to afforded the acetoxy amine salt **18a** along with it’s De-acylated amine salt (**18a-OH**) (16 mg, 65.5% in total) as a white solid. ^1^H NMR (600 MHz, DMSO-*d*_*6*_, 298 K): δ 8.41–8.25 (m, 2H), 8.01–7.54 (m, 2H), 7.35–7.28 (m, 1H), 7.20–7.00 (m, 3H), 6.99–6.87 (m, 1H), 4.83 (t, *J* = 15.1 Hz, 1H), 4.37–4.29 (m, 1H), 4.19–4.01 (m, 1H), 3.67–3.42 (m, 1H), 3.19–2.90 (m, 4H), 2.90–2.68 (m, 2H), 2.64–2.51 (m, 2H), 2.38–2.05 (m, 4H), 1.87–1.44 (m, 6H), 1.43–1.02 (m, 5H). ESI-MS (m/z): 417 Da [M + H]^+^.

### (3S,6S)-3-amino-4,8-dioxo-6-(((S)-2-oxopyrrolidin-3-yl) methyl)-5,9-diaza-1(1,3)-benzenacyclopentadecaphane-7-yl acetate hydrochloride (**18b**)

Following the procedure described for the preparation of compound **17a** with a slight modification of conducting the reaction with 4 M HCl in dioxane (0.354 ml, 10 eq.) in DCM at rt, afforded the acetoxy amine **18b** and hydroxy amine **18b-OH** (43 mg, 20 mg, in total 93%) after purification Prep-HPLC as a white solid. 18b-OH- ^1^H NMR (700 MHz, DMSO-*d*_*6*_, 298 K): δ 8.33 (s, 1H), 7.60–7.32 (m, 2H), 7.16–6.86 (m, 3H), 6.54 (s, 1H), 5.81 (d, *J* = 5.7 Hz, 1H), 4.06–3.92 (m, 1H), 3.70 (dd, *J* = 5.7, 2.3 Hz, 1H), 3.47–3.36 (m, 1H), 3.20–2.79 (m, 4H), 2.65–2.52 (m, 1H), 2.39–1.83 (m, 5H), 1.73–1.00 (m, 8H). ESI-MS (m/z): 431 Da [M + H]^+^.

### (3S,6S)-3-amino-7-hydroxy-6-(((S)-2-oxopyrrolidin-3-yl) methyl)-5,9-diaza-1(1,3)-benzenacyclohexadecaphane-4,8-dione hydrochloride (**18c**)

Following the procedure described for the preparation of compound **18a** with a slight modification of conducting the reaction with 4 M HCl in dioxane (0.1 ml, excess) in DCM at rt, afforded the hydroxy amine salt **18c-OH** (8.1 mg, 75%) as a white solid. ^1^H NMR (500 MHz, DMSO-*d*_*6*_, 298 K): δ 8.22–7.90 (m, 2H), 7.70–7.51 (m, 2H), 7.23–6.90 (m, 3H), 6.12 (d, *J* = 5.1 Hz, 1H), 4.08–3.97 (m, 1H), 3.71 (s, 1H), 3.21–2.77 (m, 6H), 2.60–2.54 (m, 2H), 2.35–1.79 (m, 7H), 1.71–1.12 (m, 8H). ESI-MS (m/z): 445 Da [M + H]^+^.

### (3S,6S)-3-amino-4,8-dioxo-6-(((S)-2-oxopyrrolidin-3-yl) methyl)-5,9-diaza-1(1,3)-benzenacycloheptadecaphane-7-yl acetate hydrochloride (**18 d**)

Following the procedure described for the preparation of compound **18a** with a slight modification of conducting the reaction with 4 M HCl in dioxane (0.2 ml, excess) in DCM (0.5 ml) at rt, afforded the **18d** along with hydroxy amine salt **18d-OH** (10 + 4 mg, 78%, in total) as a white solid. 18d-OH_Dia-1: ^1^H NMR (500 MHz, DMSO-*d*_*6*_, 298 K): δ 8.23 (s, 1H), 7.69 (t, *J* = 12.5 Hz, 1H), 7.60–7.47 (m, 1H), 7.29–6.93 (m, 3H), 6.10 (broad s, 1H), 5.52–5.28 (m, 1H), 4.10–4.01 (m, 1H), 3.95–3.48 (m, 5H), 3.25–3.00 (m, 4H), 2.94–2.52 (m, 4H), 2.39–2.25 (m, 1H), 2.19–1.85 (m, 2H), 1.74–1.04 (m, 10H). ESI-MS (m/z): 459 Da [M + H]^+^.

18d-OH_Dia-2: ^1^H NMR (500 MHz, DMSO-*d*_*6*_, 298 K): δ 8.23–7.65 (m, 2H), 7.51 (dt, *J* = 10.7, 6.8 Hz, 2H), 7.20–6.91 (m, 4H), 6.20–6.06 (m, 1H), 4.19–4.02 (m, 1H), 3.96–3.81 (m, 1H), 3.77–3.41 (m, 2H), 3.20–2.78 (m, 6H), 2.70–2.52 (m, 3H), 2.35–1.84 (m, 5H), 1.71–1.0 (m, 12H). ESI-MS (m/z): 459 Da [M + H]^+^.

### Synthesis of (3S,6S,E/Z)-3-amino-4,8-dioxo-6-(((S)-2-oxopyrrolidin-3-yl)methyl)-5,9-diaza-1(1,3)-benzenacyclopentadecaphan-13-en-7-yl acetate Hydrochloride (**18e**)

Following the procedure described for the preparation of compound **18a** with a slight modification of conducting the reaction with 4 M HCl in dioxane (0.2 ml, excess) in DCM (0.5 ml) at rt, afforded the acetoxy amine salt **18e** (7.1 mg, 79.5%) as a white solid. ^1^H NMR (500 MHz, DMSO-*d*_*6*_, 298 K): δ 8.57–7.97 (m, 2H), 7.95–7.51 (m, 2H), 7.45–6.97 (m, 3H), 6.48–6.17 (m, 1H), 6.09–5.29 (m, 2H), 5.13–4.81 (m, 1H), 4.41–3.94 (m, 2H), 3.42–2.71 (m, 6H), 2.32–1.76 (m, 4H), 1.76–1.36 (m, 3H), 1.36–0.71 (m, 5H). ESI-MS (m/z): 471 Da [M + H]^+^.

### (3S,6S)-4,8-dioxo-6-(((S)-2-oxopyrrolidin-3-yl) methyl)-3-(2-phenylacetamido)-5,9-diaza-1(1,3)-benzenacyclotetradecaphane-7-yl acetate (**19a**)

To a solution of phenyl acetic acid (5.3 mg, 0.04 mmol) in DMF, HATU (15 mg, 0.0397 mmol) was added at 0 °C. The reaction mixture was stirred at 0 °C under a nitrogen atmosphere, and after few minutes, a pre-stirred solution of amine TFA salt **18a** (13 mg, 0.0281 mmol) and triethylamine (17 mg, 0.168 mmol) in DMF at 0 °C was added. The reaction was continued for 5 h at 0 °C. Progress of the reaction was monitored based on TLC and LCMS. The reaction mixture was acidified with 10%TFA, filtered and crude was further purified on reverse-phase preparative HPLC which afforded acetoxy amide **19a** (10.2 mg, 63.4%) as a white solid. ^1^H NMR (500 MHz, DMSO-*d*_*6*_, 298 K): δ 8.60–8.50 (m, 0.5H), 8.41–8.28 (m, 0.5H), 8.09–8.01 (m, 1H), 7.62–7.45 (m, 1H), 7.38–6.84 (m, 9H), 6.86–6.32 (t, *J* = 9.5 Hz, 1H), 4.74–4.59 (m, 1H), 4.58–4.46 (m, 1H), 4.29–4.03 (m, 1H), 3.71–3.41 (m, 3H), 3.37–3.18 (m, 2H), 3.15–2.87 (m, 3H), 2.83–2.55 (m, 2H), 2.22–1.93 (m, 4H), 1.86–1.49 (m, 3H), 1.47–0.84 (m, 8H). ESI-MS (m/z): 577 Da [M + H]^+^.

### (3S,6S)-4,8-dioxo-6-(((S)-2-oxopyrrolidin-3-yl) methyl)-3-(2-phenylacetamido)-5,9-diaza-1(1,3)-benzenacyclopentadecaphane-7-yl acetate (**19b**)

Following the procedure described for the preparation of compound **19a** with a slight modification of conducting the reaction with compound **18b** (50 mg, 0.1207 mmol) and 2-phenylacetic acid (20 mg, 0.147 mmol) afforded compound **19b** (21 mg, 35%) as a white solid. ^1^H NMR (700 MHz, DMSO-*d*_*6*_, 298 K): δ 8.30 (dx2, *J* = 7.6 Hz, 1H), 7.98–7.87 (m, 1H), 7.75–7.50 (m, 3H), 7.35–6.85 (m, 8H), 4.91–4.77 (m, 1H), 4.53–4.35 (m, 1H), 4.24–4.02 (m, 1H), 3.58–3.44 (m, 2H), 3.18–2.66 (m, 6H), 2.22–1.96 (m, 4H), 1.95–1.46 (m, 5H), 1.41–0.80 (m, 9H). ESI-MS (m/z): 591 Da [M + H]^+^.

### N-((3S,6S)-7-hydroxy-4,8-dioxo-6-(((S)-2-oxopyrrolidin-3-yl) methyl)-5,9-diaza-1(1,3)-benzenacyclohexadecaphane-3-yl)-2-phenylacetamide (**19c**)

Following the procedure described for the preparation of compound **19a** with a slight modification of conducting the reaction with compound **18c-OH** (6 mg, 0.0107 mmol) and 2-phenylacetic acid (2.2 mg, 0.0161 mmol) afforded hydroxy compound **19c** (3.5 mg, 58%) as a white solid. ^1^H NMR (700 MHz, DMSO-*d*_*6*_, 298 K): δ 8.77 (d, *J* = 4.2 Hz, 1H), 8.57–8.38 (m, 1H), 8.13 (s, 1H), 7.81–7.41 (m, 2H), 7.39–7.14 (m, 3H), 7.13–6.84 (m, 3H), 6.02–5.71 (m, 2H), 4.44–4.35 (m, 1H), 4.08–4.03 (m, 1H), 3.84–3.74 (m, 1H), 3.61–3.43 (m, 2H), 3.18–2.70 (m, 5H), 2.31–1.86 (m, 5H), 1.73–1.33 (m, 5H), 1.33–0.80 (m, 8H). ESI-MS (m/z): 563 Da [M + H]^+^.

### (3S,6S)-4,8-dioxo-6-(((S)-2-oxopyrrolidin-3-yl) methyl)-3-(2-phenylacetamido)-5,9-diaza-1(1,3)-benzenacycloheptadecaphane-7-yl acetate (**19d**)

Following the procedure described for the preparation of compound **19a** with a slight modification of conducting the reaction with compound **18 d** (10 mg, 0.0186 mmol) and 2-phenylacetic acid (3.8 mg, 0.02830 mmol) afforded compound **19 d** (7.1 mg, 61%) as a white solid. ^1^H NMR (500 MHz, DMSO-*d*_*6*_, 298 K): δ 8.46–8.40 (m, 1H), 8.02–7.92 (m, 2H), 7.81–7.53 (m, 3H), 7.43–6.94 (m, 7H), 4.87–4.70 (m, 2H), 4.52–4.25 (m, 1H), 4.24–3.91 (m, 2H), 3.61–3.48 (m, 2H), 3.25–2.91 (m, 4H), 2.99–2.70 (m, 2H), 2.64–2.57 (m, 1H), 2.17–1.99 (m, 3H), 1.81–0.80 (m, 10H). ESI-MS (m/z): 619 Da [M + H]^+^.

### (3S,6S)-4,8-dioxo-6-(((S)-2-oxopyrrolidin-3-yl) methyl)-3-(3-phenylpropanamido)-5,9-diaza-1(1,3)-benzenacyclopentadecaphane-7-yl acetate (**19e**)

Following the procedure described for the preparation of compound **19a** with a slight modification of conducting the reaction with compound **18b** (52 mg, 0.102 mmol) and 3-phenylpropanoic acid (23 mg, 0.153 mmol) afforded compound **19e** (23 mg, 40%) as a white solid. ^1^H NMR (700 MHz, DMSO-*d*_*6*_, 298 K): δ 8.30–8.11 (m, 1H), 7.76–7.52 (m, 2H), 7.31–6.83 (m, 10H), 4.84 (dx2, *J* = 5.1 Hz, 1H), 4.56–4.33 (m, 1H), 4.22–3.99 (m, 1H), 3.25–2.92 (m, 4H), 2.86–2.57 (m, 5H), 2.49–2.27 (m, 4H), 2.26–1.80 (m, 6H), 1.71–0.96 (m, 9H), ESI-MS (m/z): 605 Da [M + H]^+^.

### (3S,6S)-3-((S)-3,3-dimethyl-2-(2,2,2-trifluoroacetamido) butanamido)-4,8-dioxo-6-(((S)-2-oxopyrrolidin-3-yl) methyl)-5,9-diaza-1(1,3)-benzenacyclopentadecaphane-7-yl acetate (**19 f**)

Following the procedure described for the preparation of compound **19a** with a slight modification of conducting the reaction with compound **18b-OH** (30 mg, 0.0696 mmol) and (S)-3,3-dimethyl-2-(2,2,2-trifluoroacetamido) butanoic acid (23.7 mg, 0.1044 mmol) afforded compound **19f** (12 mg, 27%) as a white solid. ^1^H NMR (700 MHz, DMSO-*d*_*6*_, 298 K): δ 9.27–9.19 (m, 1H), 8.62–8.37 (m, 1H), 7.84–7.39 (m, 2H), 7.23–6.91 (m, 4H), 6.19–5.73 (broad s, 1H), 4.53–4.29 (m, 2H), 4.02–3.53 (m, 4H), 3.17–2.95 (m, 3H), 2.95–2.70 (m, 3H), 2.64–2.54 (m, 2H), 2.46–2.28 (m, 2H), 2.21–2.07 (m, 1H), 2.01–1.78 (m, 2H), 1.73–1.45 (m, 3H), 1.44–0.77 (m, 12H). ESI-MS (m/z): 640 Da [M + H]^+^.

### (3S,6S)-4,8-dioxo-6-(((S)-2-oxopyrrolidin-3-yl) methyl)-3-(3-(2,2,2-trifluoroethyl) ureido)-5,9-diaza-1(1,3)-benzenacyclopentadecaphane-7-yl acetate (**19g**)

To a solution of amine salt **18b** (35 mg, 0.0597 mmol) in dry CH_2_Cl_2_, *N*,*N*-diisopropylethyl amine (42 μL, 0.240 mmol) was added at 0 °C. After stirring for few minutes, 2-trifluoroethyl isocyanate (15 mg, 0.12 mmol) was added at the same temperature and stirred at rt under inert conditions. The reaction was continued for 24 h, and the progress of the reaction was monitored based on TLC and LCMS. After completion of the reaction, it was quenched with 0.1 mL of methanol and evaporated to dryness. The resulting crude was purified based on a flash column over silica gel (5–10% MeOH/CH_2_Cl_2_), which gave the desired product (19 mg, 53.3%). ^1^H NMR (500 MHz, DMSO-*d*_*6*_, 298 K): δ 7.92–7.76 (m, 2H), 7.61–7.54 (m, 1H), 7.22–6.78 (m, 5H), 6.56–6.21 (m, 1H), 4.94–4.76 (m, 1H), 4.55–4.30 (m, 1H), 4.26–4.05 (m, 1H), 3.88–3.74 (m, 2H), 3.19–2.95 (m, 4H), 2.83–2.57 (m, 2H), 2.24–2.04 (m, 5H), 2.09–1.92 (m, 1H), 1.80–1.09 (m, 11H). ESI-MS (m/z): 598 Da [M + H]^+^.

### (3S,6S,E/Z)-4,8-dioxo-6-(((S)-2-oxopyrrolidin-3-yl)methyl)-3-(3-phenylpropanamido)-5,9-diaza-1(1,3)-benzenacyclopentadecaphan-13-en-7-yl acetate (**19h**)

Following the procedure described for the preparation of compound **18a** with a slight modification of conducting the reaction with compound **18e** (7 mg, 0.0138 mmol) and 3-phenylpropanoic acid (4.3 mg, 0.0266 mmol) afforded compound **19 h** (4.1 mg, 48%) as a white solid. ^1^H NMR (700 MHz, DMSO-*d*_*6*_, 298 K): δ 8.51–8.06 (m, 1H), 8.02–7.84 (m, 1H), 7.73–7.48 (m, 2H), 7.38–6.80 (m, 8H), 6.43–5.97 (m, 1H), 5.69–5.19 (m, 1H), 5.02–4.56 (m, 1H), 4.49–4.19 (m, 1H), 4.04–3.78 (m, 1H), 3.25–2.97 (m, 6H), 2.94–2.66 (m, 3H), 2.37–1.78 (m, 6H), 1.72–1.12 (m, 5H), ESI-MS (m/z): 603 Da [M + H]^+^.

### 2-phenyl-N-((3S,6S)-4,7,8-trioxo-6-(((S)-2-oxopyrrolidin-3-yl) methyl)-5,9-diaza-1(1,3)-benzenacyclotetradecaphane-3-yl) acetamide (**20a**)

To the stirred solution of compound **19a** (10 mg, 0.017 mmol) in aq. CH_3_OH, LiOH·H_2_O (1.45 mg, 0.034 mmol) was added and stirred at rt for 1.5 h. The progress of the reaction was checked on LCMS. After completion of the reaction, it was further purified on reverse-phase preparative HPLC, which afforded of a hydroxyl product (4.5 mg, 48%) as a white solid. ESI-MS (*m*/*z*): 535 Da [M + H]^+^.

Dess–Martin periodinane (DMP) (5.39 mg, 0.0126 mmol) was added to a solution of the above hydroxy amide compound (4.5 mg, 0.0084 mmol) in DMF (2 mL). The mixture was stirred at rt for 2 h. After completion of the reaction, it was treated with few drops of a Na_2_S_2_O_3_ solution and concentrated. The product was further purified on reverse-phase preparative HPLC, which afforded macrocyclic α-ketoamide **20a** (3.7 mg, 58%) as a white solid. HPLC purity: 97.4%, ^1^H NMR (700 MHz, DMSO-*d*_*6*_, 298 K): δ 8.62–8.42 (m, 2H), 8.35–8.19 (m, 2H), 7.63–7.44 (sx2, 1H), 7.33–7.17 (m, 4H), 7.14–6.71 (m, 2H), 5.22–5.16 (m, 1H), 4.58–4.41 (m, 1H), 3.62–3.42 (m, 2H), 3.19–2.84 (m, 5H), 2.25–1.91 (m, 3H), 1.80–1.11 (m, 9H). ^13^C NMR (176 MHz, DEPT-135, DMSO-*d*_*6*_) δ: 129.04, 128.89, 128.00, 127.83, 126.81, 126.32, 53.62, 49.68, 41.80, 39.87, 39.18, 39.12, 38.37, 37.71, 37.14, 34.60, 32.24, 29.98, 27.35, 26.83, 25.40, −0.04. HRMS: calcd. for C_30_H_37_N_4_O_5_ [M + H]^+^, m/z = 533.2763 Da; found, m/z = 533.2768 Da.

### 2-phenyl-N-((3S,6S)-4,7,8-trioxo-6-(((S)-2-oxopyrrolidin-3-yl) methyl)-5,9-diaza-1(1,3)-benzenacyclopentadecaphane-3-yl) acetamide (**20b**)

Compound **20b** was prepared in two steps in a 41.3% yield by deprotection of acetoxy amide **19b** (21 mg, 0.0359 mmol), followed by oxidation with DMP (21.66 mg, 0.051 mmol) utilizing the same procedure as described for compound **20b**. HPLC purity: 99.1%, ^1^H NMR (500 MHz, DMSO-*d*_*6*_, 298 K): δ 8.89–8.69 (m, 1H), 8.65–8,52 (m, 1H), 8.26–7.91 (m, 1H), 7.72–7.59 (m, 1H), 7.36–7.17 (m, 4H), 7.17–6.86 (m, 3H), 6.76–6.63 (m, 2H), 5.44–5.25 (m, 1H), 4.90–4.53 (m, 1H), 4.30–4.22 (m, 1H), 3.58–3.37 (m, 3H), 3.16–3.0 (m, 2H), 2.97–2.73 (m, 3H), 2.46–2.02 (m, 4H), 2.01–1.78 (m, 1H), 1.72–1.21 (m, 9H). ^13^C NMR (176 MHz, DMSO-*d*_*6*_, 298 K): δ 197.49, 178.29, 170.68, 170.23, 160.39, 158.62, 142.14, 136.80, 136.72, 129.97, 129.48, 128.60, 128.25, 127.45, 126.75, 53.82, 50.62, 42.43, 39.27, 38.04, 35.11, 32.60, 29.93, 27.81, 27.25, 25.45. HRMS: calcd. for C_31_H_39_N_4_O_5_ [M + H]^+^, m/z = 547.2920 Da; found, m/z = 547.2915 Da.

### 2-phenyl-N-((3S,6S)-4,7,8-trioxo-6-(((S)-2-oxopyrrolidin-3-yl) methyl)-5,9-diaza-1(1,3)-benzenacyclohexadecaphane-3-yl) acetamide (**20c**)

Compound **20c** was prepared in 63% yield from hydroxy amide **19c** (3.5 mg, 0.0062 mmol) by oxidation with DMP (3.96 mg, 0.00931 mmol). The reaction mixture was stirred at room temperature for 2 h. After completion of the reaction, a few drops of Na₂S₂O₃ solution were added, and the mixture was concentrated. The crude product was purified by reverse-phase preparative HPLC to afford macrocyclic α-ketoamide **20c**. HPLC purity: 95%. ^1^H NMR (700 MHz, DMSO-*d*_*6*_, 298 K): δ 8.80–8.61 (m, 2H), 7.84 (d, *J* = 7.6 Hz, 1H), 7.65–7.47 (m, 2H), 7.37–6.94 (m, 6H), 6.75–6.64 (m, 1H), 5.37–5.29 (m, 1H), 4.61 (dd, *J* = 12.0, 6.1 Hz, 1H), 3.61–3.41 (m, 3H), 3.20–3.04 (m, 2H), 3.01–2.56 (m, 4H), 2.49–2.32 (m, 2H), 2.31–2.14 (m, 2H), 1.95–1.45 (m, 7H), 1.40–1.00 (m, 7H). ^13^C NMR (176 MHz, DMSO-*d*_*6*_, DEPT-Q, 298 K): δ 196.97, 179.15, 177.82, 170.49, 169.81, 160.11, 141.84, 136.43, 136.36, 129.14, 128.82, 128.10, 128.07, 127.91, 127.68, 127.15, 126.59, 126.42, 126.31, 126.18, 54.81, 53.19, 53.00, 50.98, 41.91, 37.96, 37.66, 37.52, 37.43, 34.63, 31.98, 29.49, 27.24, 26.83, 26.58, 26.45, 26.23, 26.03, 23.67, 23.38. HRMS: calcd. for C_32_H_41_N_4_O_5_ [M + H]^+^, m/z = 561.3076 Da; found, m/z = 561.3074 Da.

### 2-phenyl-N-((3S,6S)-4,7,8-trioxo-6-(((S)-2-oxopyrrolidin-3-yl) methyl)-5,9-diaza-1(1,3)-benzenacycloheptadecaphane-3-yl) acetamide (**20 d**)

Compound **20d** was prepared in two steps in a 59% yield by de-protection of acetoxy amide **19d** (7 mg, 0.0113 mmol), followed by oxidation with DMP (5.8 mg, 0.0138 mmol) utilizing the same procedure as described for compound **20a**. HPLC purity: 97.8%, ^1^H NMR (700 MHz, DMSO-*d*_*6*_, 298 K): δ 8.76–8.63 (m, 1H), 8.59–8.46 (m, 1H), 8.20–7.92 (m, 1H), 7.74–7.45 (m, 2H), 7.33–7.15 (m, 3H), 7.14–6.78 (m, 3H), 6.92–6.79 (m, 1H), 5.13–5.21 (m, 1H), 4.63–4.46 (m, 1H), 3.60–3.40 (m, 3H), 3.20–2.97 (m, 2H), 2.93–2.76 (m, 3H), 2.67–2.42 (m, 2H), 2.33–1.94 (m, 3H), 1.96–1.78 (m, 1H), 1.69–1.40 (m, 5H), 1.40–1.04 (m, 8H). ^13^C NMR (176 MHz, DMSO-*d*_*6*_, 298 K): δ 197.47, 179.48, 178.64, 171.48, 170.28, 161.57, 141.94, 137.43, 137.05, 130.30, 129.55, 129.07, 128.43, 128.32, 127.56, 126.73, 54.45, 51.92, 42.60, 42.42, 38.29, 37.88, 34.85, 31.99, 30.33, 28.59, 27.59, 27.26, 25.80. HRMS: calcd. for C_33_H_43_N_4_O_5_ [M + H]^+^, m/z = 575.3233 Da; found, m/z = 575.3231 Da.

### 3-phenyl-N-((3S,6S)-4,7,8-trioxo-6-(((S)-2-oxopyrrolidin-3-yl) methyl)-5,9-diaza-1(1,3)-benzenacyclopentadecaphane-3-yl) propanamide (**20e**)

Compound **20e** was prepared in two steps in a 65% yield by deprotection of acetoxy amide **19e** (23 mg, 0.04 mmol), followed by oxidation with DMP (21 mg, 0.049 mmol) utilizing the same procedure as described for compound **20a**. HPLC purity: 97.2%, ^1^H NMR (700 MHz, DMSO-*d*_*6*_, 298 K): δ 8.79–8.70 (m, 1H), 8.57 (t, *J* = 9.9 Hz, 1H), 7.82–7.79 (m, 1H), 7.66–7.59 (m, 1H), 7.31–7.14 (m, 5H), 7.07 (t, *J* = 7.5 Hz, 1H), 6.95 (t, *J* = 10.8 Hz, 1H), 6.73 (d, *J* = 7.6 Hz, 1H), 6.68 (s, 1H), 5.29 (s, 1H), 4.61–4.58 (m, 1H), 3.40 (s, 1H), 3.17–3.07 (m, 2H), 2.90 (d, *J* = 13.5 Hz, 1H), 2.85–2.73 (m, 4H), 2.48–2.36 (m, 3H), 2.31–2.15 (m, 2H), 1.85 (d, *J* = 17.1 Hz, 1H), 1.68 (d, *J* = 12.3 Hz, 1H), 1.56–1.19 (m, 2H). ^13^C NMR (176 MHz, DMSO-*d*_*6*_, 298 K): δ 197.94, 177.33, 171.51, 170.78, 160.36, 158.97, 141.98, 137.15, 129.91, 128.75, 127.75, 126.67, 126.29, 53.84, 50.72, 40.01, 39.99, 39.84, 39.31, 38.75, 38.64, 38.24, 37.98, 35.08, 32.67, 31.51, 29.90, 27.33, 27.29, 25.46. HRMS: calcd. for C_32_H_41_N_4_O_5_ [M + H]^+^, m/z = 561.3076 Da; found, m/z = 561.3071 Da.

### (S)-3,3-dimethyl-2-(2,2,2-trifluoroacetamido)-N-((3S,6S)-4,7,8-trioxo-6-(((S)-2-oxopyrrolidin-3-yl) methyl)-5,9-diaza-1(1,3)-benzenacyclopentadecaphane-3-yl) butanamide (**20f**)

Compound **20c** was prepared in 67% yield from hydroxy amide **19f** (15 mg, 0.022 mmol) by oxidation with DMP (9.9 mg, 0.0234 mmol). The reaction mixture was stirred at room temperature for 2 h. After completion of the reaction, a few drops of Na₂S₂O₃ solution were added, and the mixture was concentrated. The crude product was purified by reverse-phase preparative HPLC to afford macrocyclic α-ketoamide **20f**. HPLC purity: 95.4%, ^1^H NMR (500 MHz, DMSO-*d*_*6*_, 298 K): δ 9.38–9.16 (m, 1H), 8.77–8.62 (m, 1H), 8.58–8.29 (m, 2H), 7.69–7.54 (m, 1H), 7.17–6.99 (m, 3H), 6.81–6.61 (m, 1H), 5.28 (d, *J* = 17.8 Hz, 1H), 4.89–4.52 (m, 1H), 4.54–4.31 (m, 2H), 3.18–2.74 (m, 5H), 2.48–2.08 (m, 4H), 2.033–1.73 (m, 2H), 1.70–0.77 (m, 15H). ^13^C NMR (126 MHz, DMSO-*d*_*6*_, 298 K): δ 197.27, 178.53, 170.80, 168.71, 160.64, 141.95, 137.18, 129.80, 128.27, 127.10, 126.84, 61.45, 60.64, 55.31, 50.23, 40.55, 40.22, 40.13, 39.96, 39.79, 39.46, 37.61, 35.17, 35.04, 32.94, 29.98, 28.25, 27.91, 27.06, 26.89, 25.80. HRMS: calcd. for C_31_H_43_F_3_N_5_O_6_ [M + H]^+^, m/z = 638.3165 Da; found, m/z = 638.3162 Da.

### 1-(2,2,2-trifluoroethyl)-3-((3S,6S)-4,7,8-trioxo-6-(((S)-2-oxopyrrolidin-3-yl) methyl)-5,9-diaza-1(1,3)-benzenacyclopentadecaphane-3-yl) urea (**20g**)

Compound **20g** was prepared in two steps in a 69% yield by deprotection of acetoxy amide **19g** (18 mg, 0.030 mmol), followed by oxidation with DMP (16 mg, 0.0378 mmol) utilizing the same procedure as described for compound **20a**. HPLC purity: 99.4%, ^1^H NMR (500 MHz, DMSO-*d*_*6*_, 298 K): δ 8.99–8.91 (m, 1H), 8.82–8.68 (m, 1H), 7.75–7.62 (sx2, 1H), 7.14 (t, *J* = 15.6 Hz, 1H), 7.02–6.92 (m, 2H), 6.91–6.78 (m, 1H), 6.58 (sx2, 1H), 6.19–6.13 (m, 1H), 5.48–5.34 (m, 1H), 4.82–4.35 (m, 1H), 3.97–3.78 (m, 2H), 3.17–3.10 (m, 2H), 2.99–2.74 (m, 3H), 2.49–2.26 (m, 3H), 2.24–2.10 (m, 1H), 2.02–1.82 (m, 2H), 1.74–1.63 (m, 1H), 1.61–1.18 (m, 8H). ^13^C NMR (126 MHz, DMSO-*d*_*6*_, 298 K): δ 197.81, 178.15, 171.05, 159.85, 156.78, 142.12, 136.65, 136.63, 130.32, 128.09, 128.06, 127.69, 126.64, 53.5, 50.5, 39.96, 38.06, 35.35, 32.12, 30.05, 27.89, 27.71, 26.72, 25.24. HRMS: calcd. for C_26_H_35_F_3_N_5_O_5_ [M + H]^+^, m/z = 554.2590 Da; found, m/z = 554.2585 Da.

### 3-phenyl-N-((3S,6S)-4,7,8-trioxo-6-(((S)-2-oxopyrrolidin-3-yl) methyl)-5,9-diaza-1(1,3)-benzenacyclopentadecaphan-13-en-3-yl) propanamide (**20h**)

Compound **20h** was prepared in two steps in a 53% yield by deprotection of acetoxy amide **19h** (4.1 mg, 0.0068 mmol), followed by oxidation with DMP (3.4 mg, 0.008 mmol) utilizing the same procedure as described for compound **20a**. HPLC purity: 99.8%, *E/Z* ratio = 1:4. ^1^H NMR (700 MHz, DMSO-*d*_*6*_, 298 K): δ 8.66–8.45 (m, 2H), 8.26–8.11 (m, 1H), 8.11–7.99 (m, 1H), 7.96–7.84 (m, 1H), 7.70–7.59 (m, 1H), 7.31–7.06 (m, 5H), 7.06–6.90 (m, 2H), 6.90–6.64 (m, 2H), 5.56–5.33 (m, 2H), 5.02 (dd, *J* = 15.3 Hz, 1H), 4.68–4.14 (m, 2H), 3.33–3.18 (m, 3H), 3.17–2.95 (m, 3H), 2.91–2.69 (m, 4H), 2.49–2.36 (m, 2H), 2.29–1.83 (m, 6H), 1.69–1.44 (m, 4H). ^13^C NMR (176 MHz, DMSO-*d*_*6*_, DEPT-135, 298 K): δ139.85, 132.47, 129.48, 128.13, 126.55, 125.68, 53.99, 50.86, 47.94, 39.87, 39.26, 39.14, 38.63, 37.76, 37.40, 36.37, 32.51, 31.79, 30.86, 29.43, 27.30, 26.59, 23.39. HRMS: calcd. for C_32_H_39_N_4_O_5_ [M + H]^+^, m/z = 559.2920 Da; found, m/z = 559.2917 Da.

### Antiviral activity assays against SARS-CoV-2 in A549^ACE2+TMPRSS2^ cells

The antiviral activity against SARS-CoV-2 Zagreb isolate (SARS-CoV2/ZG/297-20, University Hospital for Infectious Diseases, Zagreb, Croatia, GISAID database ID: EPI_ISL_451934)^53^ on A549^ACE2+TMPRSS2^ (obtained from the Goethe-University Frankfurt)^46^ was assessed as described previously^40^. In brief, compounds were added at a final volume of 100 µl in 10 different concentrations starting at 50 µM diluted by twofold. Each compound was tested in three to four technical replicates and at least two biological replicates. The cells were infected at BSL-3 level with SARS-CoV-2 wild-type at an MOI of 0.005. On day three post infection, cellular viability was analyzed by measuring the luminescence after addition of cell titer glo reagent (CellTiter-Glo® Substrate (lyophilized, #G755A, Promega) and CellTiter-Glo® Buffer (#G756A, Promega)). EC_50_ values were calculated in GraphPad Prism using a nonlinear regression—exponential decay ([Inhibitor] vs. response with variable slope for **20b,** **20e** and **20f** and normalized response for **20g** and **20h**).

### Cytotoxicity measurements in A549^ACE2+TMPRSS2^ cells

A549^ACE2+TMPRSS2^ cells were seeded at a density of 2.0 × 10^4^ cells/well in incubation media (DMEM + 2% FBS) in a 96-well flat-bottom cell culture plate (Corning, REF 353075) and incubated at 37 °C and 5% CO_2_. On the following day, the compounds were serially diluted in DMEM + 2% FBS and added at a final volume of 100 µl in 10 different concentrations starting at 50 µM diluted by twofold. Each compound was tested in three to four technical replicates. Additionally, one column per plate was treated with 0.5% DMSO in incubation media without any test compound used as negative control. After 71 h of incubation, 0.1% Triton reagent (×-100, Sigma-Aldrich, STBH3768) was added to three wells per plate as positive control. Cell viability was determined after 72 h of incubation by measuring the absorbance at 450 nm using the Infinite® 200 PRO Tecan Microplate Reader. For that, 10 µl of Cell Counting Kit-8 reagent (Dojindo Laboratories, CK04-11) was added to each well and incubated at 37 °C and 5% CO_2_ for 2 h_._ The measured sample absorbance (A_sample_) was normalized as followed: ((A_sample_ – A_triton_)/(A_untreated_ – A_triton_)) × 100%, where A_triton_ is the average absorbance of all trition treated wells (positive control) and A_untreated_ is the average absorbance of all untreated wells per plate (negative control).

### Cell culture

HeLa-R19 (ATCC, CCL-2) and HEK293T (ATCC, CRL-3216) were seeded and maintained in Dulbecco’s Modified Eagle’s Medium (DMEM) supplemented with 10% (V/V) fetal bovine serum (FBS) and 100 μg/ml penicillin/streptomycin (P/S).

### Production of recombinant luciferase reporter viruses

CVB3 (Nancy strain), EV-D68 (Fermon strain) and EV-A71 (BrCr strain) cDNA sequences were cloned in an expression vector under the transcriptional control of a T7 polymerase promoter. In the case of CVB3, the Renilla luciferase gene was inserted upstream of the VP4 gene and separated by a 3C/3D cleavage site. For EV-D68 and EV-A71, the Renilla luciferase gene was inserted between the VP1 and 2A gene, flanked by 2A cleavage sites. Viral RNA was transcribed in vitro from these plasmids (RiboMAX, Promega) and transfected into HEK293T cells with Lipofectamine2000 (Invitrogen). When total cytopathic effect (CPE) was reached (2–4 days), medium was harvested and subjected to three freeze-thaw cycles, followed by centrifugation at 4000 × *g* for 15 min to pellet cell debris. Supernatant was collected and stored as virus stocks at −80 °C. Viral titers (TCID_50_) were determined on HeLa-R19 cells and calculated with the Spearman-Kärber method using the average of three independent titrations.

### Infection and antiviral activity assays using reporter viruses

HeLa-R19 cells were seeded in flat-bottom 96-well plates (10^4^ cells per well) at least 16 h prior to infection experiments. Cells were infected with recombinant luciferase reporter viruses (MOI 0.1) for 30 min at 37 °C (CVB3 and EV-A71) or 33 °C (EV-D68). After 30 min of infection, the viral inoculum was aspirated and the cells were incubated with medium containing varying concentrations of **20f** for 6.5 h at 37 °C (CVB3 and EV-A71) or 33 °C (EV-D68). All medium was removed 7 h post-infection and the cells were lysed with Renilla luciferase lysis buffer (Promega). Renilla luciferase activity was measured using the Renilla luciferase assay system (Promega) with a GloMax Explorer luminometer (Promega). Cell viability assays were performed parallel to infection experiments, but did not include virus infection. Cell viability was measured using the MTS assay with Aqueous One Solution (Promega) according to manufacturer’s protocol.

### Antiviral activity assays measuring EV-D68-induced cytopathic effects

To test for antiviral activity against enterovirus D68, RD cells (ATCC, CCL-136) were seeded at a density of 2 × 10^4^ cells per well in DMEM supplemented with 10% FBS in a 96-well flat-bottom cell culture plate, and incubated at 37 °C in a 5% CO_2_ atmosphere. The following day, the inhibitors were serially diluted in DMEM containing 10% FBS to a final volume of 100 µl. The RD cells were treated using ten different concentrations, starting at 100 µM and diluted by a factor of two. Each inhibitor was tested in three technical replicates. After 24 h of treatment, the compounds were removed, and the cells were infected with EV-D68 virus (ATCC, VR-1824) at a multiplicity of infection of 0.1 in DMEM containing 2% FBS and incubated at 33 °C in a 5% CO_2_ atmosphere for one hour. One hour after infection, the virus was removed and the same concentrations of compounds were added to the cells, this time prepared in DMEM supplemented with 2% FBS. After 72 h, the incubation was stopped and cell viability was assessed using crystal violet.

To measure cell viability, the medium was removed and the cells were washed once with 100 µl of PBS. The cells were then fixed by adding 50 µl of methanol to each well for one minute. This was then replaced with 100 µl of a 0.1% crystal violet solution prepared in distilled water and the plate was shaken for 30 min at room temperature on a rocking platform, protected from light. The cells were then washed three times with 100 µl of PBS. After air-drying the plate for at least two hours, 100 µl of methanol was added to dissolve the crystal violet. The plate was then shaken for 30 min on a rocking platform, protected from light. Absorbance was measured at 570 nm using the Tecan Sunrise plate reader. The measured absorbance was normalized using the following formula: -(1-(A_infected_ – A_sample_)/(A_infected_ – A_uninfected_) × 100% and EC_50_ values were calculated in GraphPad Prism 10 using nonlinear regression (dose-response–Asymmetric (five parameter), X is log(concentration)).

### Cytotoxicity assessments in RD cells

RD cells (ATCC, CCL-136) were seeded at a density of 2 × 10^4^ cells per well in DMEM supplemented with 10% FBS in a 96-well flat-bottom cell culture plate, and incubated at 37 °C in a 5% CO_2_ atmosphere. The following day, the inhibitors were serially diluted in DMEM containing 10% FBS to a final volume of 100 µl. Ten different concentrations were used, starting at 100 µM and diluted by a factor of two. Each inhibitor was tested in three technical replicates. Cell viability was measured after five days of incubation by determining the crystal violet absorbance at 570 nm using the Tecan Sunrise plate reader. Cell viability was calculated as followed: (A_compound treated cells_/A_untreated cells_) × 100%.

### Cloning and recombinant protein production

A gene encoding the SARS-CoV-2 main protease (M^pro^ wild type, GenBank reference: MN908947.3 (ORF1ab polyprotein residues 3264–3569)) with *E. coli* codon usage was produced by MWG Eurofins. The amplified DNA fragment was digested with the restriction enzymes *Bam*HI and *Xho*I and then ligated into the plasmid pGEX-6P-1. The WT M^pro^ coding gene with *Bam*HI and *Xho*I sites was amplified from the M^pro^ construct described previously^54^ and was used as a template. The gene sequence of the M^pro^ was verified by sequencing (MWG Eurofins, Ebersberg, Germany). For recombinant protein production, the SARS-CoV-2 M^pro^ PGEx6p-1 plasmid was transformed into BL21 (DE3) competent cells and the protein was produced as described before^3^.

### Determination of inhibition concentrations (IC_50_’s)

A fluorescent substrate considering the cleavage site of SARS-CoV2 M^pro^ (Dabcyl-KTSAVLQ ↓ SGFRKM-E (Edans)-NH_2_), the cleavage site of Enterovirus D68 (Dabcyl-KTATVQ ↓ GPSLD-E (Edans)-NH_2_ × TFA, and buffer composed of 20 mM HEPES, 120 mM NaCl, 0.4 mM EDTA, 4 mM DTT, 20% Glycerol, pH 7.0 was used for the inhibition assay. In the fluorescence resonance energy transfer (FRET)-based cleavage assay^55^, the fluorescence signal of the Edans generated due to the cleavage of the substrate by the M^pro^ was observed at an emission wavelength of 460 nm with excitation at 360 nm fluorescence spectrophotometer (BioTek). Tecan—The Spark Control—Multimode Microplate reader was used for the inhibition test. The compound was diluted with 100% DMSO to prepare a stock solution. IC_50_ was determined with the incubation of 50 nM of SARS-CoV2 M^pro^, 1 µM of Enterovirus D68, and compound at various concentrations from 0 to 100 µM in reaction buffer at 37 °C for 10 min. In the end, the FRET substrate at a final concentration of 50, 10, and 10 µM was added to each well for M^pro^, EV-D68 3C^pro^, and EV-71 3C^pro^, respectively, at a final total volume of 100 µl to initiate the reaction. The IC_50_ value was calculated with the help of GraphPad Prism software. Inhibitory activity of the compound was measured in triplicates.

### Determination of inhibition rates against host cell proteases cathepsin B, K, L

Cathepsin B (CTSB) from human liver was provided from Enzo Life Sciences (BML-SE198) and diluted to a 500 nM stock solution in 50 mM NaOAc, pH 5.0, 1 mM EDTA and 2.5 mM DTT. The benzyloxycarbonyl-protected dipeptide Z-Phe-Arg-MCA (4-methylcoumaryl-7-amide) was used as a fluorogenic substrate for cathepsin B and L (Peptanova 3095-v).

Active human cathepsin K (CTSK) from Sigma Aldrich (SRP6561) was diluted to a 50 nM stock solution in 100 mM NaOAc, pH 5.5, 2.5 mM Na_2_EDTA and 2.5 mM DTT. The fluorogenic substrate Z-Leu-Arg-MCA was also provided from Peptanova (3210-v) and is widely used as a substrate for cathepsin K.

All compounds were dissolved in 100% DMSO. To determine the inhibition rates at fixed concentrations of 1 and 10 µM, predilutions of 0.1 and 1 mM were made in 100% DMSO. Since 1 µl of compound is used in a total volume of 100 µl, the final DMSO concentration is 1%. 10 µl of stock solutions of either cathepsin B, K, or L (final concentrations: 50 nM CTSB, 5 nM CTSK, 25 nM CTSL) were preincubated with 1 or 10 µM compound in the same buffer condition recommended for each protease stock. After 10 min incubation at 37 °C, the proteolytic reaction was started by adding 30 µl of the respective substrate (final concentration 10 µM; exception 5 µM for CTSK reaction). The increase of fluorescence was detected using a Multimode Microplate Reader, BioTek Synergy H1 from Agilent (*λ*_ex_ = 360 nm; *λ*_em_ = 460 nm). All compounds were tested in triplicates. DMSO instead of compound served as a negative control and was set as the highest (100%) activity rate. Taking the initial slope, the activity rate was calculated related to the DMSO control. The inhibition rate (difference of activity rate to 100%) is shown in Table S8.

### Crystallization and diffraction data collection of M^pro^ in complex with macrocyclic α-ketoamides

The purified SARS-CoV-2 M^pro^ and Enterovirus D68-3C^pro^ proteins were concentrated to 14 and 6 mg/mL, respectively. Proteins were then incubated overnight with a fivefold molar excess of inhibitor, and the mixture was incubated overnight at 4 °C. After being centrifuged at 13,000 rpm to remove precipitates. The supernatants of the SARS-CoV-2 M^pro^ compound mixture were then subjected to co-crystallization screening with commercially available kits (PACT premierTM HT-96, Morpheus and LFS Screen from Molecular Dimensions, Rotherham, UK), using an Art Robbins Instruments robot and the vapor diffusion sitting drop method at 20 °C. The Enterovirus D68-3C^pro^ were subjected to manual vapor diffusion sitting drop co-crystallization gradient screening of 0.1 M Tris-HCl pH 7.2–8.5, 0.2 M ammonium acetate, 15–35% w/v PEG 3350 at 4 °C. Diffraction data from the M^pro^ in complex with inhibitors were collected from crystals grown under the conditions of LSF No.-H5 (0.1 M SPG, pH 6.5, 25% w/v PEG 1500) for **9b-K**, LSF No.-H7 (0.1 M Bis-Tris, pH 6.5, 25% w/v PEG 3350) for **20b**, Morpheus No.-E5 ((0.1 M Sodium HEPES; MOPS (acid) pH 7.5 30% w/v (40% v/v PEG 500 MME; 20% w/v PEG 20000)), 0.1 M Ethylene glycol) for **20f** and **20g**, PACT No.-A4 (0.1 M SPG, pH 9.0, 25% w/v PEG 1500) for **20e**, PACT No.-A3 (0.1 M SPG, pH 8.0, 25% w/v PEG 1500) for **20h**, LSF No.-D10 (0.1 M PTCP, pH 8.0, 30% w/v PEG 1000) for **20a**. Diffraction data from the Enterovirus D68-3C^pro^ in complex with inhibitors were collected from crystals grown under the conditions of 0.1 M Tris-HCl pH 7.6–8, 0.2 M Ammonium acetate, 20–25% w/v PEG 3350. Crystals were then cryo-protected using 20% glycerol. Fished crystals were flash-cooled using liquid nitrogen. Diffraction data were collected at 100 K at DESY PETRA III beamline P11 (Hamburg, Germany) using a Pilatus 6 M detector (Dectris) and with synchrotron radiation at a wavelength of 1.0332 Å.

### Diffraction data processing, structure solution, and refinement

The programs XDSapp^56^, Pointless^55,57^^,^, and Scala^58^ were used for dataset processing, with Molrep^57,58^ to solve the phase problem via the molecular replacement method using the SARS-CoV-2 M^pro^ free enzyme (PDB code: 6Y2E) and the Enterovirus D68-3C^pro^ free enzyme (PDB code: 3ZV8), All compounds were built into Fo–Fc difference densities using the Coot software 0.9.8.91^59^ and structures were refined using the Refmac5 program^60^. All graphical illustrations and RMSD values were calculated using PyMOL 4.6^61^. Diffraction data and model refinement statistics are displayed in Tables S2 and S3.

### ADME in vitro studies

The plasma stability assay, the metabolic stability assay as well as the plasma protein binding assay were conducted as described previously^62^.

### HPLC-MS/MS analysis

Samples were analyzed using an Agilent 1290 Infinity II HPLC system coupled to an AB Sciex QTrap 6500plus mass spectrometer using the same LC conditions as described previously^63^. Mass transitions for controls and compounds are depicted in Table S4.

Additionally, an Agilent 1290 Infinity II HPLC system coupled to an AB SCIEX Triple Quad^TM^ 7500 mass spectrometer was used with previously described LC conditions^63^. Mass transitions for controls and compounds are depicted in Table S5.

### Cellular accumulation assay on A549^ACE2+TMPRSS2^ cells

Cellular accumulation was assessed as described previously^63^. In brief, the compounds **20b** and **20e–h** were added at a final concentration of 25 µM to A549-AT cells in a 96-well format. After a 1 h incubation period at 37 °C and 5% CO_2_, intracellular concentrations were received by HPLC-MS/MS analysis. Mass transitions can be found in Tables S4 and S5. A calibration curve ranging from 0.1 to 10000 nM as well as quality control samples (QCs; concentrations 1, 10, 100, and 1000 nM) were prepared for all compounds and measured simultaneously. Finally, K_*p*_ values were calculated after accounting for nonspecific binding and determining the cellular volume per well.

## Supplementary information

Transparent Peer Review file

Supporting Information

## Data Availability

The authors declare that the data supporting the findings of this study are available within the paper and its Supplementary Information files. Should any raw data files be needed in another format, they are available from the corresponding author upon reasonable request. Crystallographic coordinates and structure factors are available from the PDB under accession codes 9T5F (M^pro^ complex with **20f** in space group *C*2), 9SSO (M^pro^ complex with **20g** in space group *C*2), 9T72 (M^pro^ complex with **20h** in space group *C*2), 9T5E (M^pro^ complex with **20a** in space group *C*2), 9SSN (M^pro^ complex with **9b-K** in space group *C*2), 9T52 (M^pro^ complex with **20b** in space group *C*2), 9T55 (M^pro^ complex with **20e** in space group *C*2), 29CT (3C^pro^ in complex with **20e**) and 29DA (3C^pro^ in complex with **20g**)
