# Supplementary material for: Structure-based macrocyclization of α-ketoamides leads to potent inhibitors of coronaviral and enteroviral proteases
Source: Commun Chem. 2026 Aug 6;9:269. doi: 10.1038/s42004-026-02151-y (PMC13448699; doi:10.1038/s42004-026-02151-y)
Supplement: Supplementary file 1 [file 42004_2026_2151_MOESM1_ESM.pdf]

nature portfolio

Peer Review File

Structure-based macrocyclization of α-ketoamides leads to potent inhibitors of coronaviral and enteroviral proteases

Corresponding Author: Professor Mark Broenstrup

This file contains all reviewer reports in order by version, followed by all author rebuttals in order by version.

Version 0:

Reviewer comments:

Reviewer #1

(Remarks to the Author)
Akula et al. present the synthesis and thorough characterization of a set of macrocyclic inhibitors for the main protease (MPro) of SARS CoV-2 and similar viral proteases. Starting from the binding mode of linear MPro inhibitors they derive two cyclization strategies incorporating two different warheads (nitiles and a-ketoamides). For the latter series, highly active inhibitors were found that were characterized for enzyme inhibition, ADME parameters, binding mode and antiviral activity in cells. Notably, some analog retained activity against enteroviral proteases and against the corresponding enteroviruses. This demonstration of a broader activity scope is highly important to prepare for possible new viral pandemics. Moreover, the in-depth structural analyses allow a very high degree of understanding how these compounds work at the biochemical level. Collectively, this manuscript is highly suited for ChemComms but would benefit from addressing the following minor issues:

1. Fig. 2B: It would be more appropriate to measure the distance from carbon attached to the nitrogen of the gamma-lactam to the benzylic position of the P4 substitutent since these more closely reflect the exit vectors in the later macrocycles

2. Fig. 2C: It would be more appropriate to measure the distance from beta-carbon of the P2 substituent to the nitrogen of the diketo amide since these positions more closely reflect the exit vectors in the later macrocycles

3. The classification of hydrogen bonds needs careful revision. Which parameters/cutoffs did the authors use to classify a contact as a hydrogen bond? Numerous claimed hydrogen bonds are at odds with my definition, incl. (i) from Ser144 (3.5 Å; questionable geometry); (ii) Gly143 (3.5 Å); (iii) weak interaction with His41 Nε2 (3.2 Å); (iv) His163 Nε2 (2.8 Å; difficult to judge, was not shown in figure); (v) Glu166 Cε2 (unclear geometry); (vi) backbone carbonyl og Glu155(3.6 Å)
Similar considerations apply to the analyses of the other crystal structures

4. Line 284: Provide the E/Z ratio for compound 20h (e.g. from NMR). Please avoid cis/trans nomenclature for double bonds.

5. Scheme 3, compounds 16a-d: This structure (vinyl directly attached to phenyl) applies to 16a&c, but not to 16b&d. For the latter, n should be n=1 and 3 and there should be a methylene between the vinyl and the phenyl since these are the analogs without double bond isomerization.
Alternatively, the authors could define n = 1, 3 and introduce a second variable (e.g., m = 0, 1) for the allyl substituent on the phenyl ring, and apply this consistently across the scheme.

6. In Scheme 4, the formation of the desired macrocycle via RCM is correctly depicted; however, it should be labeled as 16b rather than 16a.

7. Figure 4-6: It would be great to keep the same perspective and alignment for all complexes

8. Fig. 6: Maybe a detailed picture for one of 20a,b or h would be appropriate (as for 20g in Fig. 5), since these seem to have a different binding mode

9. Tab 1: At which concentration were the 17%/10% inhibition determined for 20a/c for 3Cpro. For 20h, indicate that this is an E/Z mixture

10. Line 393: 'This potentially explains the weak cellular activity'. These are important measurements but they don't explain the cellular activity trends.

11. Tab 2; How precise are these numbers?

12. Line 403: What are 'cysteine pockets'?

13. The potent activity of 20f against EV-D68 is amazing. Can the authors explain this somehow? It is not evident from the biochemical activities on EV-D68 3Cpro

14. References 32 and 24 are rather old. The author might consider Brudy at al., J Med Chem 2024 (10.1021/acs.jmedchem.4c01163) and Viarengo-Baker and Whitty, J Med Chem 2025 (doi: 10.1021/acs.jmedchem.4c02886), who specifically analyse ring-closing strategies from linear precursors and the effects underlying macrocylces

15. The battery of structures of closely related macrocycles is highly interesting from a macrocycle design perspective. The prepare macrocycles can be expected to be highly flexible and indeed seem to adopt multiple conformations depending on minor chemical changes. The authors might want to discuss this in more detail, possibly also in comparison with Spiske et al (ACIE 2025, doi: 10.1002/anie.202418511) and Buffa et a (ACIE 2025, doi: 10.1002/anie.202418512), where a similar behaviour and the impact on multiple drug-relevant parameters was observed.

16. The authors may consider including the structure of compound 11r, which is mentioned in line 77.

17. In line 100, compound 6d-K is not shown in Figure 1.

18. Did the authors attempt to optimize the RCM conditions? For example, using Grubbs II catalyst to minimize the formation of side products.

19. For compound 20h, did the authors attempt to separate cis and trans isomers by HPLC?

20. For some compounds, such as 20b, two peaks can be observed in the HPLC traces. Could the authors clarify the origin of these peaks?

Reviewer #2

(Remarks to the Author)
The work by Haifa El Kilani, Mark Brönstrup et al is about the synthesis and testing of macrocyclic ketoamides as inhibitors of SARS-CoV-2 and antivirals. An extensive work has been performed and under my view is publishable in Comm Chem. The work includes synthesis of a number of inhibitors, crystallographic studies, and a number of biological studies including antiviral testing. It is remarkable how the authors prepared new compounds by incorporating functionalizations guided by crystallographic studies and enzymatic testing. As a result of the work compound 20f was identified as a hit with nanomolar activity against enteroviruses.

Please consider the following corrections before publications:
-In the abstract says "The 17-membered macrocycle and α-ketoamide 20f inhibited Mpro with an IC_{50} _value of 370 nM" but should say "The 17-membered macrocycle α-ketoamide 20f inhibited Mpro with an IC_{50} _value of 370 nM"
-In page 3 when says "Recently, the groups of Güntschow, Müller and Sofia found macrocyclic compounds inhibiting Mpro of SARS-CoV-2 with a covalently binding azapeptide nitrile and a noncovalently binding peptidomimetic, respectively (Figure 1).38,39", the code of the corresponding compounds should be indicated in the sentence. Please also correct author to Gütschow.
-In page 5 says "led to the P1-P3-linked macrocyclic nitriles shown in Figure 3A as target structures for 123 synthesis." but only one cyclic inhibitors is depicted in Figure 3A so better rephrase it.
-It can be a little confusing to talk about inhibitors 11a and 12c of previous papers and having compounds 11 and 12 from this work, not quite sure best way to fix it.
-The Figure 3 could be improved: draw bigger chemical structures, shorter retrosynthesis arrows, get chemical compounds and their numbers closer,… Indicate disconnections or reactions over the retrosynthesis arrows. Depict stereochemistry of the stereocenter of the lactam ring in cpds 15a, 18b, 20f correctly (as in Scheme 2). The Boc structure should be indicated in the same way for cpds 14 and 15a (also consistent alomg the manuscript).
-In Scheme 1, the stereochemistry of cpd 1 is not indicated (if a racemate is used it should be mentioned). In conditions d says "5-(((benzyloxy) carbonyl) amino)carboxylic acid" but it should be corrected.
-The chemical structure for cpd 16a in Scheme 4 is not the same one as indicated in Scheme 3.

Reviewer #3

(Remarks to the Author)
This is a well-executed and interesting manuscript. The study presents a clear structure-based design strategy, solid

synthetic work, extensive co-crystal structures, and a reasonably comprehensive biological characterization. In particular, I find the enterovirus-related results highly encouraging. Compound 20f shows remarkably potent cellular antiviral activity against EV-D68, EV-A71, and CVB3, which substantially increases the impact of the work beyond SARS-CoV-2 Mpro inhibition. Overall, the manuscript is of good quality and potentially suitable for publication after revision.
Major concerns
1.The manuscript should avoid direct cross-assay comparison of IC50 values between SARS-CoV-2 Mpro and enteroviral 3Cpro. The authors conclude that the order of biochemical activity is Mpro (SARS-CoV-2) > 3Cpro (D68). However, according to the assay description, the SARS-CoV-2 Mpro inhibition assay was performed with 50 nM enzyme, whereas the enteroviral 3Cpro assay was performed with 1 μM enzyme. Given these substantially different assay conditions, direct comparison of IC50 values across the two proteases is not rigorous and may be misleading. In particular, the EV-D68 3Cpro IC50 values of 1.4–2.9 μM were obtained in the presence of a much higher enzyme concentration, which in fact still suggests strong target-level activity for this series. Together with the very potent cellular antiviral activity of 20f against enteroviruses, the current data support the conclusion that this scaffold is highly promising for enteroviral targets as well. I therefore suggest that the authors remove or substantially tone down any direct statement implying that these compounds are intrinsically better inhibitors of SARS-CoV-2 Mpro than of enteroviral 3Cpro based solely on IC50 comparisons.
2.For SARS-CoV-2 Mpro, inclusion of a reference inhibitor such as nirmatrelvir would provide an important benchmark for readers to assess the potency of the macrocyclic series. For enteroviral 3Cpro, a standard reference inhibitor such as rupintrivir (AG7088) should also be included in the enzymatic assay. I note that rupintrivir was used as a positive control in the EV-D68 cell-based assay, which is helpful, but equivalent benchmarking at the biochemical level is still lacking. Such controls would make the activity claims more transparent and allow easier comparison with prior literature.
3.The enterovirus data are promising, but the claim of broad enteroviral 3Cpro inhibition would be better supported by including biochemical assays against EV-A71 and CVB3 3Cpro, since the current mechanistic data are centered on EV-D68 3Cpro, whereas EV-A71 and CVB3 are represented mainly by cell-based antiviral results.
4.Since these compounds are reversible covalent α-ketoamide inhibitors targeting cysteine proteases, some assessment of selectivity against representative human proteases would substantially strengthen the manuscript.
5.Several interaction plots are overly cluttered, especially Figures 2A, 4A, 4C, and 5A. I suggest that the authors optimize the viewing angles and simplify these panels by removing non-essential residues and other distracting structural elements, so that the key interactions relevant to the discussion are more clearly emphasized.

Version 1:

Reviewer comments:

Reviewer #3

(Remarks to the Author)
The authors have addressed all my concerns, and I have no further concerns.

Open Access This Peer Review File is licensed under a Creative Commons Attribution 4.0 International License, which permits use, sharing, adaptation, distribution and reproduction in any medium or format, as long as you give appropriate credit to the original author(s) and the source, provide a link to the Creative Commons license, and indicate if changes were made.

In cases where reviewers are anonymous, credit should be given to 'Anonymous Referee' and the source.
The images or other third party material in this Peer Review File are included in the article's Creative Commons license, unless indicated otherwise in a credit line to the material. If material is not included in the article's Creative Commons license and your intended use is not permitted by statutory regulation or exceeds the permitted use, you will need to obtain permission directly from the copyright holder.
To view a copy of this license, visit https://creativecommons.org/licenses/by/4.0/

Point-by-point response

The original comment is printed in black. The author's response is printed in blue, and changes to the text are printed with yellow background.

Reviewers' comments:

Reviewer #1 (Remarks to the Author):

Akula et al. present the synthesis and thorough characterization of a set of macrocyclic inhibitors for the main protease (MPro) of SARS CoV-2 and similar viral proteases. Starting from the binding mode of linear MPro inhibitors they derive two cyclization strategies incorporating two different warheads (nitiles and a-ketoamides). For the latter series, highly active inhibitors were found that were characterized for enzyme inhibition, ADME parameters, binding mode and antiviral activity in cells. Notably, some analog retained activity against enteroviral proteases and against the corresponding enteroviruses. This demonstration of a broader activity scope is highly important to prepare for possible new viral pandemics. Moreover, the in-depth structural analyses allow a very high degree of understanding how these compounds work at the biochemical level. Collectively, this manuscript is highly suited for ChemComms but would benefit from addressing the following minor issues:

1. Fig. 2B: It would be more appropriate to measure the distance from carbon attached to the nitrogen of the gamma-lactam to the benzylic position of the P4 substituent since these more closely reflect the exit vectors in the later macrocycles

Response: We followed the suggestion of the reviewer and modified Fig 2B, the text and the figure caption accordingly.

A close inspection of crystallographic data on **12c** bound to M^{pro} (PDB: 8AIZ), revealed an 8.6 Å spatial separation between the γ-carbon of the lactam side chain in P1 and the benzylic position of the P4 substituent (Figure 2A-B).

2. Fig. 2C: It would be more appropriate to measure the distance from beta-carbon of the P2 substituent to the nitrogen of the diketoamide since these positions more closely reflect the exit vectors in the later macrocycles

Response: We followed the suggestion of the reviewer and modified Fig 2C, the text and the figure caption accordingly.

When assuming a different perspective on the co-crystal structure of **12c** to M^{pro} (PDB: 8AIZ), it became evident that also the β-carbon of the cyclopropyl residue from the P2 site and the amide nitrogen atom of the P1' site were separated by a distance of only 6.2 Å (Figure 2C).

3. The classification of hydrogen bonds needs careful revision. Which parameters/cutoffs did the authors use to classify a contact as a hydrogen bond? Numerous claimed hydrogen bonds are at odds with my definition, incl. (i) from Ser144 (3.5 Å; questionable geometry); (ii) Gly143 (3.5 Å); (iii) weak

interaction with His41 Nε2 (3.2 Å); (iv) His163 Nε2 (2.8 Å; difficult to judge, was not shown in figure); (v) Glu166 Cε2 (unclear geometry); (vi) backbone carbonyl og Glu155(3.6 Å)
Similar considerations apply to the analyses of the other crystal structures

Response: The parameters used to classify a contact as a hydrogen bond are based on the distance and angle criteria described by Kabsch & Sander, Biopolymers 22, 2577 (1983). For the sake of clarity, the text has been adjusted accordingly, and Figure 4 in the manuscript has been modified: All hydrogen bonds between the inhibitor and the corresponding Mpro residues are colored red. Yellow dashed lines highlight all other interactions.

4. Line 284: Provide the E/Z ratio for compound 20h (e.g. from NMR). Please avoid cis/trans nomenclature for double bonds.

Response: Based on the NMR analysis, compound 20h was obtained as a 4:1 mixture of diastereomers. This information was added to the analytical data.
We have replaced the cis/trans nomenclature by cis/trans nomenclature throughout the text.

5. Scheme 3, compounds 16a-d: This structure (vinyl directly attached to phenyl) applies to 16a&c, but not to 16b&d. For the latter, n should be n=1 and 3 and there should be a methylene between the vinyl and the phenyl since these are the analogs without double bond isomerization.
Alternatively, the authors could define n = 1, 3 and introduce a second variable (e.g., m = 0, 1) for the allyl substituent on the phenyl ring, and apply this consistently across the scheme.

Response: We thank the reviewer for spotting this mistake. We follow the first suggestion how to solve the issue and have adapted Scheme 3 accordingly.

6. In Scheme 4, the formation of the desired macrocycle via RCM is correctly depicted; however, it should be labeled as 16b rather than 16a.

Response: The reviewer is right (again!). We have adapted Scheme 4 accordingly.

7. Figure 4-6: It would be great to keep the same perspective and alignment for all complexes

Response: All figures have been corrected accordingly; however, a minor adjustment to the alignment is required to highlight the respective focal point of each figure.

8. Fig. 6: Maybe a detailed picture for one of 20a,b or h would be appropriate (as for 20g in Fig. 5), since these seem to have a different binding mode

Response: We followed the suggestion of the reviewer and inserted a detailed picture for 20a (instead of the mesh surface of 20g) in Fig 5.

9. Tab 1: At which concentration were the 17%/10% inhibition determined for 20a/c for 3Cpro. For 20h, indicate that this is an E/Z mixture

Response: The compounds' potency was measured at a concentration of 100 µM. We have changed table 1 accordingly.

10. Line 393: 'This potentially explains the weak cellular activity'. These are important measurements but they don't explain the cellular activity trends.

Response: This is correct. In the revised text, we highlight three limitations of the assay that might result in the non-satisfactory reflection of the cellular activity trends:

However, cellular accumulation did not account for protein binding in the cytosol, or distribution into sub-cellular organelles. It is also conceivable that uptake is altered under infection conditions. These non-quantified contributors to target engagement might account for the differences in rank order.

11. Tab 2; How precise are these numbers?

Response: The numbers for $K_{p,final}$ originate from the mean of at least three replicate measurements for K_p for cellular accumulation, substracted by K_p for non-specific binding. The two-digits after the comma are based on the calibration curve (determining the K_p for cellular accumulation (total) and the K_p for non-specific binding). In order to clarifies the determination of the $K_{p,final}$ values. In order to give the standard deviations for each individual parameter, we have added a Table S6 to the supplement.

Table S6. Cellular accumulation data.

	$K_{p[37\,°C]}$ [a)]	$K_{p[4\,°C]}$ [a)]	$K_{p,final}$ [b)]
**RHCDS-5a**	105.72 ± 26.6	22.02 ± 12.9	83.70
**20b**	3.98 ± 2.1	1.56 ± 0.3	2.42
**20e**	6.53 ± 1.3	2.96 ± 1.1	3.57
**20f**	3.10 ± 1.6	1.43 ± 0.4	1.67
**20g**	2.61 ± 0.9	1.76 ± 1.8	0.85
**20h**	5.64 ± 2.7	2.14 ± 0.2	3.50

a) mean of three replicate measurements ± s.d. b) $K_{p,final} = K_{p[37\,°C]} - K_{p[4\,°C]}$

12. Line 403: What are 'cysteine pockets'?

Response: We thank the reviewer for spotting this mistake. It is 'cysteine proteases', not 'cysteine pockets'.

13. The potent activity of 20f against EV-D68 is amazing. Can the authors explain this somehow? It is not evident from the biochemical activities on EV-D68 3Cpro.

Response: We also noticed the surprisingly high cellular activity of 20f. The EC50 value for inhibiting EV-D68 replication was determined as ca. 35 nM independently in two labs at Utrecht University and HZI. We also have no indication that the in vitro data are not correct. It could be the compound is metabolized / modified in cells yielding a more active version. However, because this is highly speculative, we prefer not to elaborate on this possibility in the manuscript.

14. References 32 and 24 are rather old. The author might consider Brudy at al., J Med Chem 2024 (10.1021/acs.jmedchem.4c01163) and Viarengo-Baker and Whitty, J Med Chem 2025 (doi: 10.1021/acs.jmedchem.4c02886), who specifically analyse ring-closing strategies from linear precursors and the effects underlying macrocylces

Response: We thank the reviewer for the hints. We deleted the former reference 34 and added the two suggested ones.

15. The battery of structures of closely related macrocycles is highly interesting from a macrocycle design perspective. The prepare macrocycles can be expected to be highly flexible and indeed seem to adopt multiple conformations depending on minor chemical changes. The authors might want to discuss this in more detail, possibly also in comparison with Spiske et al (ACIE 2025, doi: 10.1002/anie.202418511) and Buffa et a (ACIE 2025, doi: 10.1002/anie.202418512), where a similar behaviour and the impact on multiple drug-relevant parameters was observed.

Response: This suggestion would put the current antiviral compounds in a broader context of using macrocycle-based lead finding. We are grateful for the advice and strengthened the quality of the discussion with the following paragraph:

However, the macrocyclic scaffold itself has not been fully explored. While optimization efforts in this study mostly focused on finding the optimal length of an alkyl linker, discontinuous activity trends in both cellular and enzymatic assays as well as the crystal structures imply high conformational plasticity of the macrocyclic scaffold. The decoration of the cycle with heteroatoms or substituents thus offers further potential for improvements in activity through conformational restrictions, as shown in systematic macrocycle optimization programs against different targets.[49,50]

16. The authors may consider including the structure of compound 11r, which is mentioned in line 77.

Response: We followed the suggestion of the reviewer and inserted 11r in figure 1.

17. In line 100, compound 6d-K is not shown in Figure 1.

Response: The reviewer is correct. Given the limited relevance of 6d-K, we omitted this compound from the text.

18. Did the authors attempt to optimize the RCM conditions? For example, using Grubbs II catalyst to minimize the formation of side products.

Response: We tried reaction conditions previously developed in our laboratory for similar antiviral compounds (unpublished work). Most of these conditions provided good conversion and afforded only few side products. However, we did not try all variants of the RCM to find optimal conditions.

19. For compound 20h, did the authors attempt to separate cis and trans isomers by HPLC?

Response: We attempted to separate the E/Z isomers using preparative HPLC, but we were unsuccessful. This may be due to the presence of a reactive electrophilic center in the compounds, which generally makes the separation process difficult (see also next answer).

20. For some compounds, such as 20b, two peaks can be observed in the HPLC traces. Could the authors clarify the origin of these peaks?

Response: We often observe the formation of two peaks for ketoamides during purification and analysis by HPLC under acidic conditions. Because the second peak exhibits a mass of +18 Da compared to the expected mass, we ascribe it to a reversible hydrate formation upon the addition of water to the keto group. This was also the case for 20b. To make the reader aware of this property, we added the following sentence to 'General information on chemical synthesis': .

Analytical HPLC-MS traces of several compounds exhibit a second peak with a mass that is +18 Da heavier than the expected mass; this peak is ascribed to a reversible hydrate formation upon the addition of water to the keto group.

Reviewer #2 (Remarks to the Author):

The work by Haifa El Kilani, Mark Brönstrup et al is about the synthesis and testing of macrocyclic ketoamides as inhibitors of SARS-CoV-2 and antivirals. An extensive work has been performed and under my view is publishable in Comm Chem. The work includes synthesis of a number of inhibitors, crystallographic studies, and a number of biological studies including antiviral testing. It is remarkable how the authors prepared new compounds by incorporating functionalizations guided by crystallographic studies and enzymatic testing. As a result of the work compound 20f was identified as a hit with nanomolar activity against enteroviruses.

Please consider the following corrections before publications:
-In the abstract says "The 17-membered macrocycle and α-ketoamide 20f inhibited Mpro with an IC_{50} _value of 370 nM" but should say "The 17-membered macrocycle α-ketoamide 20f inhibited Mpro with an IC_{50} _value of 370 nM"

Response: We thank the reviewer for the suggestion. We inserted the following, slightly modified phrase:
The 17-membered macrocyclic α-ketoamide **20f** inhibited M^{pro} (IC_{50} = 370 nM)

-In page 3 when says "Recently, the groups of Güntschow, Müller and Sofia found macrocyclic compounds inhibiting Mpro of SARS-CoV-2 with a covalently binding azapeptide nitrile and a noncovalently binding peptidomimetic, respectively (Figure 1).38,39", the code of the corresponding compounds should be indicated in the sentence. Please also correct author to Gütschow.

Response: We thank the reviewer for spotting the mistake and adapted the sentence as follows:

Recently, the groups of Gütschow, Müller and Sofia found macrocyclic compounds inhibiting M^{pro} of SARS-CoV-2, i.e. the covalently binding azapeptide nitrile **84** and the noncovalently binding peptidomimetic **26**, respectively (Figure 1).

-In page 5 says "led to the P1-P3-linked macrocyclic nitriles shown in Figure 3A as target structures for 123 synthesis." but only one cyclic inhibitors is depicted in Figure 3A so better rephrase it.

Response: Correct. We changed the sentence to:

This led to the P1-P3-linked macrocyclic nitriles as target structures for synthesis (Figure 3A and Scheme 1).

-It can be a little confusing to talk about inhibitors 11a and 12c of previous papers and having compounds 11 and 12 from this work, not quite sure best way to fix it.

Response: This is a valid point. We decided to remove ambiguities by adding the following remark to Scheme 2:

Note that **11**, **12** and **13** are different from the previously reported compounds **11a**, **11r**, **12c** or **13b-K** shown in Figure 1.

-The Figure 3 could be improved: draw bigger chemical structures, shorter retrosynthesis arrows, get chemical compounds and their numbers closer,… Indicate disconnections or reactions over the retrosynthesis arrows. Depict stereochemistry of the stereocenter of the lactam ring in cpds 15a, 18b, 20f correctly (as in Scheme 2). The Boc structure should be indicated in the same way for cpds 14 and 15a (also consistent alomg the manuscript).

Response: We thank the reviewer for the suggestions and have improved Figure 3 and other Schemes and Figures accordingly.
-In Scheme 1, the stereochemistry of cpd 1 is not indicated (if a racemate is used it should be mentioned). In conditions d says "5-(((benzyloxy) carbonyl) amino)carboxylic acid" but it should be corrected.

Response: We corrected this.

-The chemical structure for cpd 16a in Scheme 4 is not the same one as indicated in Scheme 3.

Response: The reviewer is right. Cpd 16b is depicted in Scheme 4. We corrected this.

Reviewer #3 (Remarks to the Author):

This is a well-executed and interesting manuscript. The study presents a clear structure-based design strategy, solid synthetic work, extensive co-crystal structures, and a reasonably comprehensive biological characterization. In particular, I find the enterovirus-related results highly encouraging. Compound 20f shows remarkably potent cellular antiviral activity against EV-D68, EV-A71, and CVB3, which substantially increases the impact of the work beyond SARS-CoV-2 Mpro inhibition. Overall, the

manuscript is of good quality and potentially suitable for publication after revision.

Major concerns

1.The manuscript should avoid direct cross-assay comparison of IC50 values between SARS-CoV-2 Mpro and enteroviral 3Cpro. The authors conclude that the order of biochemical activity is Mpro (SARS-CoV-2) > 3Cpro (D68). However, according to the assay description, the SARS-CoV-2 Mpro inhibition assay was performed with 50 nM enzyme, whereas the enteroviral 3Cpro assay was performed with 1 µM enzyme. Given these substantially different assay conditions, direct comparison of IC50 values across the two proteases is not rigorous and may be misleading. In particular, the EV-D68 3Cpro IC50 values of 1.4–2.9 µM were obtained in the presence of a much higher enzyme concentration, which in fact still suggests strong target-level activity for this series. Together with the very potent cellular antiviral activity of 20f against enteroviruses, the current data support the conclusion that this scaffold is highly promising for enteroviral targets as well. I therefore suggest that the authors remove or substantially tone down any direct statement implying that these compounds are intrinsically better inhibitors of SARS-CoV-2 Mpro than of enteroviral 3Cpro based solely on IC50 comparisons.

Response: We agree with the reviewer. We have therefore removed the phrase '…with the order of activities M^{pro} (SARS-CoV-2) > $3C^{pro}$ (D68).' and avoided comparisons of the IC50 values for the different proteases.

2.For SARS-CoV-2 Mpro, inclusion of a reference inhibitor such as nirmatrelvir would provide an important benchmark for readers to assess the potency of the macrocyclic series. For enteroviral 3Cpro, a standard reference inhibitor such as rupintrivir (AG7088) should also be included in the enzymatic assay. I note that rupintrivir was used as a positive control in the EV-D68 cell-based assay, which is helpful, but equivalent benchmarking at the biochemical level is still lacking. Such controls would make the activity claims more transparent and allow easier comparison with prior literature.

Response: We follow the reviewer's suggestion and inserted enzymatic inhibition data for nirmatrelvir and rupintrivir in Table 1. While nirmatrelvir was a stronger inhibitor for SARS-CoV-2, and rupintrivir was a stronger inhibitor for EV (enzymatic and cellular) than our macrocycles, both compounds did not exert broad spectrum activity. This aspect was added to the text as follows:

However, while our macrocycles were weaker inhibitors than nirmatrelvir for SARS-CoV-2 M^{pro} and rupintrivir for EV $3C^{pro}$, the two positive controls did not exert broad-spectrum activity against both proteases. This demonstrates that dual target inhibition is non-trivial.

3.The enterovirus data are promising, but the claim of broad enteroviral 3Cpro inhibition would be better supported by including biochemical assays against EV-A71 and CVB3 3Cpro, since the current mechanistic data are centered on EV-D68 3Cpro, whereas EV-A71 and CVB3 are represented mainly by cell-based antiviral results.

Response: We agree with the reviewer that such data would further strengthen the in vitro data set. However, the stocks of several compounds were depleted. We have added data on EV-A71 3Cpro for the macrocycles **20b** and **20e** (Table S8). These data confirm inhibition also against this protease. Together with the confirmed the binding mode and the cellular antiviral assays against three enteroviruses for the best compound, we believe that the experimental evidence for enteroviral inhibition by the macrocycles is sufficient. The text and the SI were amended as follows:

For **20b** and **20e**, we could also show inhibition of the $3C^{pro}$ of the enterovirus EV-A71 with IC_{50} values of 6.9 µM and 4.4 µM, respectively (Table S7).

Table S7. Enzymatic inhibition of $3C^{pro}$ of EV-A71 by macrocycles **20b** and **20e.**

Macrocycle	EV-A71 $3C^{pro}$ IC_{50}[1] (µM)
**20b**	6.9 ± 1.7
**20e**	4.4 ± 0.5

[1] ± Standard deviation (SD), n = 3.

4.Since these compounds are reversible covalent α-ketoamide inhibitors targeting cysteine proteases, some assessment of selectivity against representative human proteases would substantially strengthen the manuscript.

Response: We follow the suggestion of the reviewer. While several compounds were depleted, we tested three representative macrocycles against the four human cysteine proteases cathepsin L, B, and K in two concentrations. The data were added as Table S7, and the methods section was expanded accordingly. Because the additional experiments were performed by Judith Röske, she was added as a co-author.

The text. The supplement and the methods section were amended as follows:

Beyond that, we also observed inhibition of human cathepsins, structurally related cysteine proteases, for the tested inhibitors **20b**, **20e** and **20h** (Table S8). The inhibition was particularly strong for cathepsin L. Because cathepsin L plays a role in the entry of SARS-CoV-2 Omicron variants such as the BA.5 strain, its inhibition has been regarded as favorable to enhance the antiviral effect;[9,49] other cathepsins are rather seen as off-targets.

Table S7. Inhibition of human cathepsins by selected macrocycles at concentrations of 1 and 10 µM**.**

	**Cathepsin L**		**Cathepsin B**		**Cathepsin K**	
Macrocycle	10 µM	1 µM	10 µM	1 µM	10 µM	1 µM
**20b**	86	75	7	2	47	8
**20e**	84	70	-4	-9	43	7
**20h**	96	87	94	48	89	57

Determination of inhibition rates against host cell proteases cathepsin B, K, L.

Cathepsin B (CTSB) from human liver was provided from Enzo Life Sciences (BML-SE198) and diluted to a 500 nM stock solution in 50 mM NaOAc, pH 5.0, 1 mM EDTA and 2.5 mM DTT. The benzyloxycarbonyl-protected dipeptide Z-Phe-Arg-MCA (4-methylcoumaryl-7-amide) was used as a fluorogenic substrate for Cathepsin B and L (Peptanova 3095-v).

Active human cathepsin K (CTSK) from Sigma Aldrich (SRP6561) was diluted to a 50 nM stock solution in 100 mM NaOAc, pH 5.5, 2.5 mM Na_2EDTA and 2.5 mM DTT. The fluorogenic substrate Z-Leu-Arg-MCA was also provided from Peptanova (3210-v) and is widely used as a substrate for Cathepsin K.

All compounds were dissolved in 100 % DMSO. To determine the inhibition rates at fixed concentrations of 1 and 10 µM, predilutions of 0.1 mM and 1 mM were made in 100 % DMSO. Since 1 µl of compound is used in a total volume of 100 µl, the final DMSO concentration is 1 %. 10 µl of stock solutions of either cathepsin B, K, or L (final concentrations: 50 nM CTSB, 5 nM CTSK, 25 nM CTSL) were preincubated with 1 or 10 µM compound in the same buffer condition recommended for each protease stock. After 10 min incubation at 37 °C, the proteolytic reaction was started by adding 30 µl of the respective substrate (final concentration 10 µM; exception 5 µM for CTSK reaction). The increase of fluorescence was detected using a Multimode Microplate Reader, BioTek Synergy H1 from Agilent (λ_{ex} = 360 nm; λ_{em} = 460 nm). All compounds were tested in triplicates. DMSO instead of compound served as a negative control and was set as the highest (100 %) activity rate. Taking the initial slope, the activity rate was calculated related to the DMSO control. The inhibition rate (difference of activity rate to 100 %) is shown in Table S8.

5. Several interaction plots are overly cluttered, especially Figures 2A, 4A, 4C, and 5A. I suggest that the authors optimize the viewing angles and simplify these panels by removing non-essential residues and other distracting structural elements, so that the key interactions relevant to the discussion are more clearly emphasized.

Response: We follow the reviewer's suggestion and adjusted the figures accordingly.
