# Supplementary material for: Structure-based macrocyclization of α-ketoamides leads to potent inhibitors of coronaviral and enteroviral proteases
Source: Commun Chem. 2026 Aug 6;9:269. doi: 10.1038/s42004-026-02151-y (PMC13448699; doi:10.1038/s42004-026-02151-y)
Supplement: Supplementary file 2 [file 42004_2026_2151_MOESM2_ESM.pdf]

Supporting information for

Structure-based macrocyclization of α-ketoamides leads to potent inhibitors of coronaviral and enteroviral proteases

Ravi Kumar Akula[1,2¶], Haifa El Kilani[2,3¶*], Alina Metzen[1,4,5], Sanjata Joshi[2], Hilal Durmaz[1,5], Robin Veenstra[6], Judith Röske[2], Daniel L. Hurdiss[6], Frank J.M. van Kuppeveld[6], Katharina Rox[1,4,5], Rolf Hilgenfeld[2,3] and Mark Brönstrup[1,4,7*]

1 Department of Chemical Biology, Helmholtz Centre for Infection Research, Inhoffenstr. 7, 38124 Braunschweig, Germany

2 Institute of Molecular Medicine, University of Lübeck, Ratzeburger Allee 160, 23562 Lübeck, Germany

3 German Center for Infection Research (DZIF), Hamburg-Lübeck-Borstel-Riems Site, 23562 Lübeck, Germany

4 German Center for Infection Research (DZIF), Hannover-Braunschweig Site, 38124 Braunschweig, Germany

5 Research Group Pharmacokinetics/Pharmacodynamics Unit, Helmholtz Centre for Infection Research, Inhoffenstr. 7, 38124 Braunschweig, Germany

6 Virology section, Division of Infectious Diseases & Immunology, Department of Biomolecular Health Sciences, Faculty of Veterinary Medicine, Utrecht University, Utrecht, The Netherlands

7 Institute of Organic Chemistry and Biomolecular Drug Research Centre (BMWZ), Leibniz University Hannover, Schneiderberg 1B, 30167, Hannover, Germany

[¶] R. K. A. and H. E. K. contributed equally to this work

*Authors for correspondence. Emails: haifa.elkilani@helmholtz-hzi.de and mark.broenstrup@helmholtz-hzi.de

**Contents**	**Pages**
**Figure S1.** Structures of synthesized final macrocyclic compounds.	**S3**
**Figure S2.** Comparison of the binding of **20e** and **20g** to the *S2 s*ubsites of SARS-CoV-2 M^{pro} and EV-D68 $3C^{pro}$*.*	**S4**
**Figure S3.** Antiviral effect of **20f** on EV-D68, EV-A71 and Coxsackievirus-B3.	**S5**
**Intermediate synthesis**	**S6**
**Table S1.** Enzyme inhibitory activity of macrocyclic nitriles against SARS-CoV-2 M^{pro}	**S7**
**Tables S2-S3.** Diffraction data and model refinement statistics	**S7- S11**
**Table S4.** Mass transitions for cellular accumulation and ADME assessments	**S12**
**Table S5.** Mass transitions for cellular accumulation	**S13**
**Table S6.** Cellular accumulation data	**S14**
**Table S7.** Enzymatic inhibition of $3C^{pro}$ of EV-A71 by macrocycles **20b** and **20e**	**S14**
**Table S8.** Inhibition of cathepsins by selected macrocycles at concentrations of 10 µM and 1 µM	**S14**
**Figure S4.** ^{1}H-NMR and ^{13}C-NMR spectra	**S15-S27**
**Figure S5.** HPLC traces	**S28-S33**
Supplementary Reference	**S33**

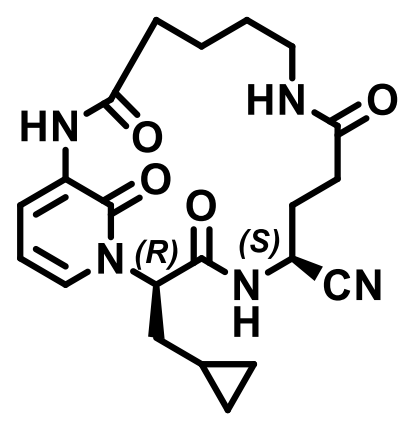

9a-H

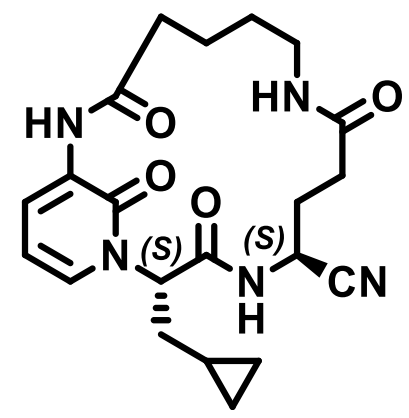

9a-K

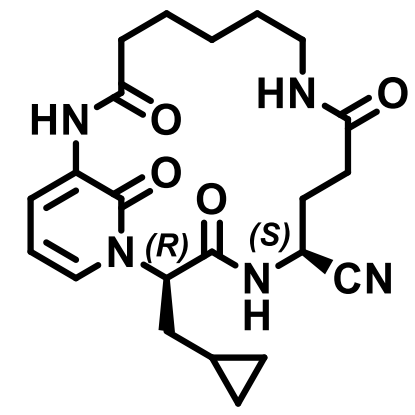

9b-H

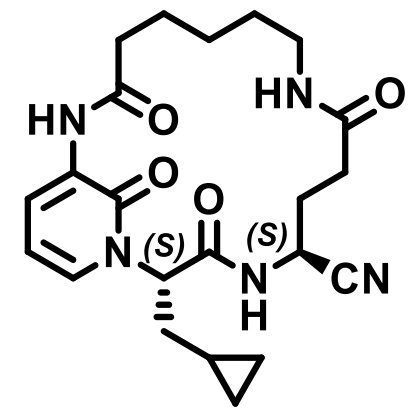

9b-K

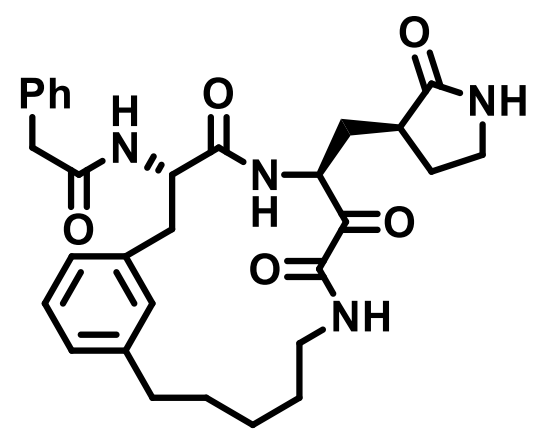

20a

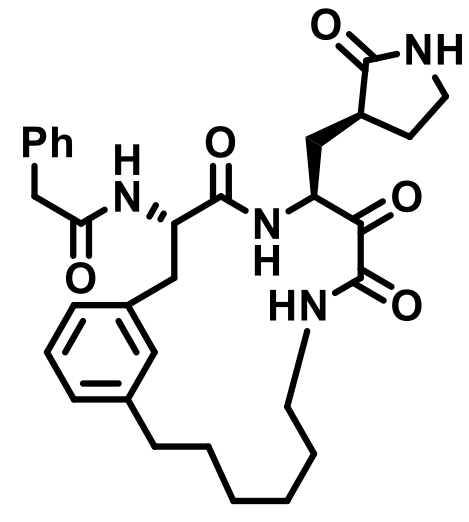

20b

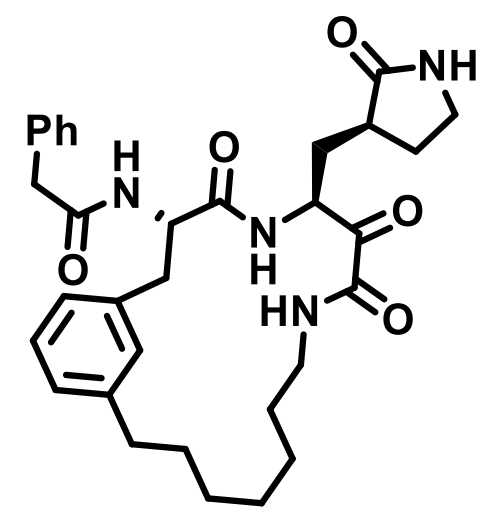

20c

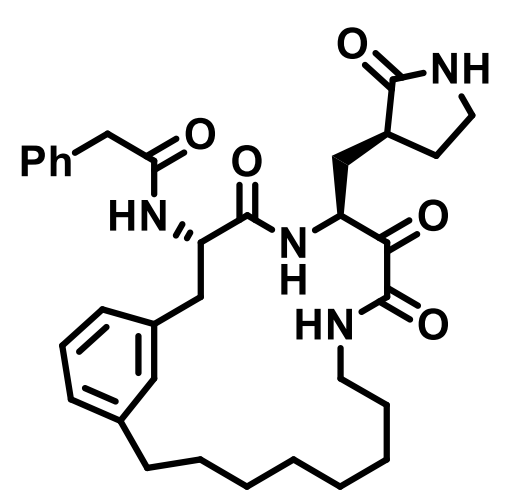

20d

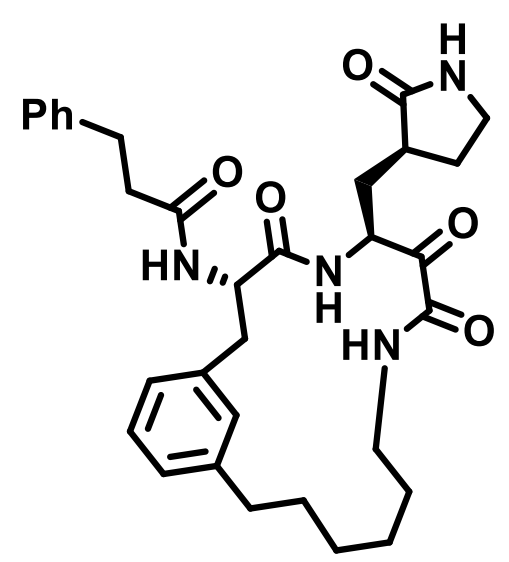

20e

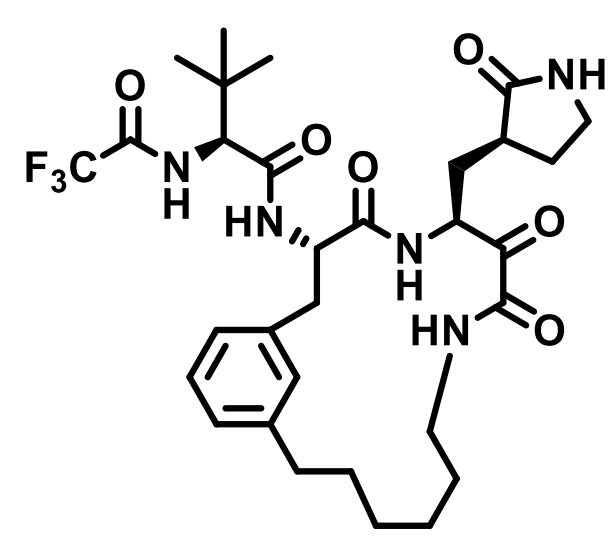

20f

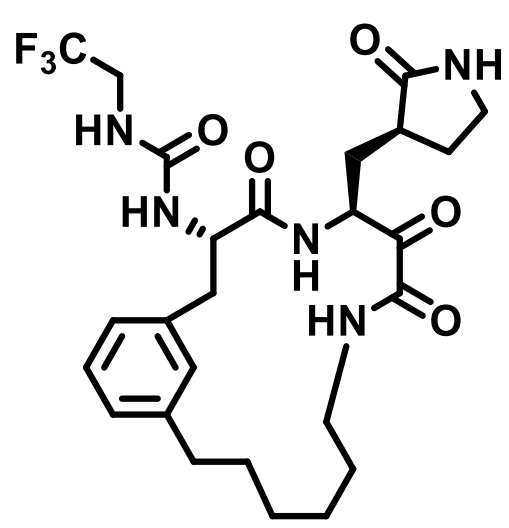

20g

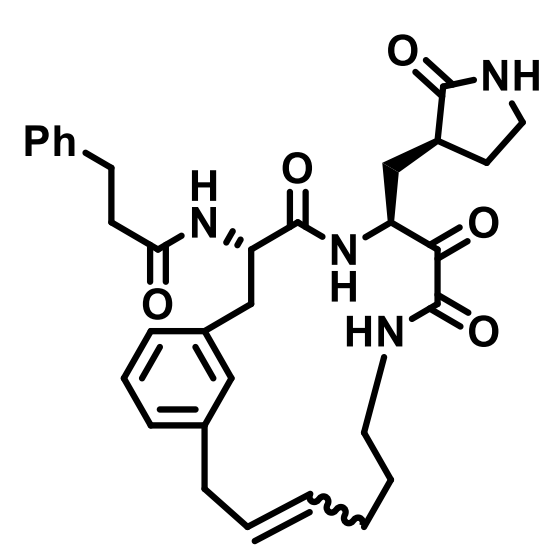

20h

Figure S1. Structures of synthesized final macrocyclic compounds.

36

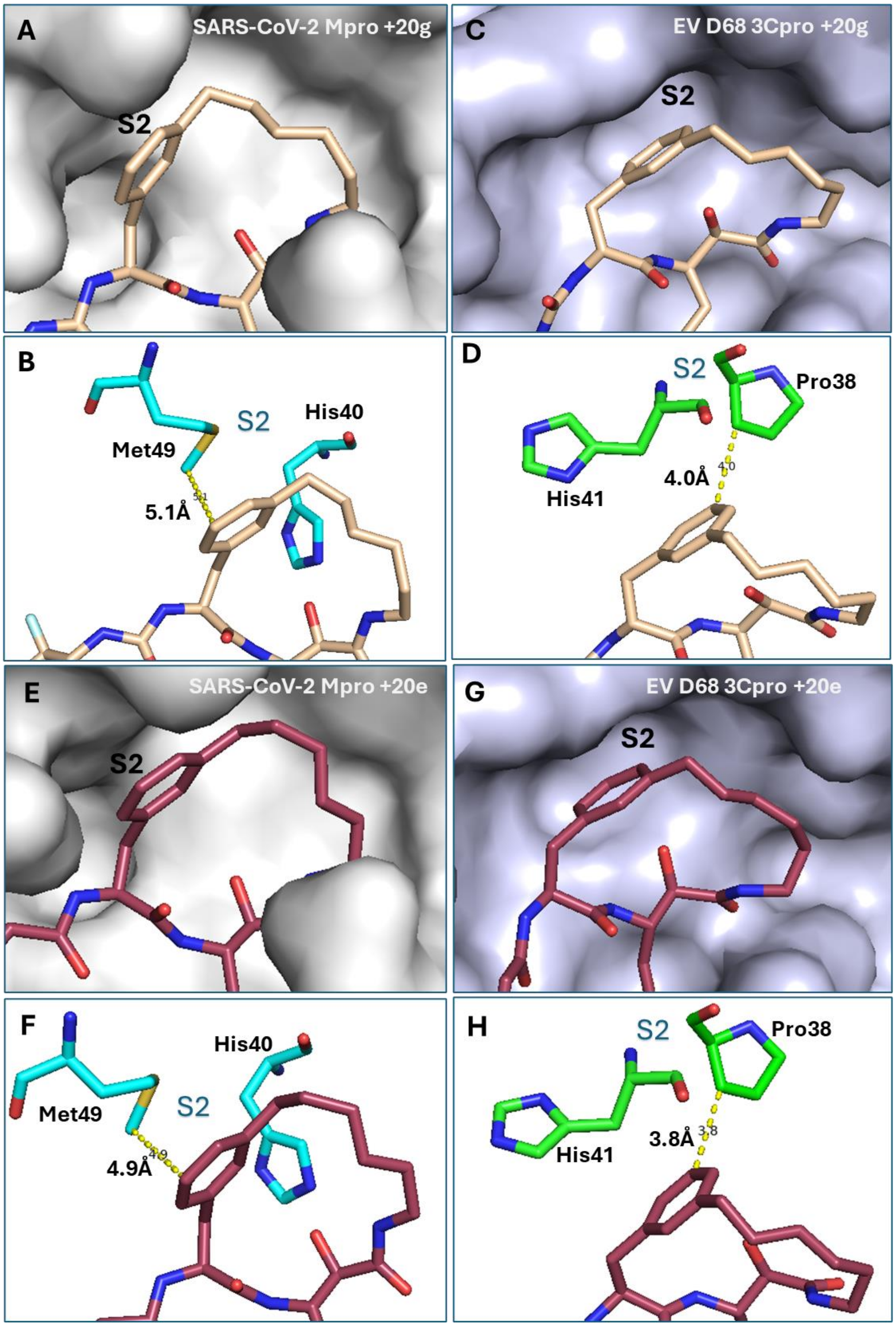

Figure S2. Comparison of the binding of **20e** and **20g** to the S2 subsites of SARS-CoV-2 M^{pro} and EV-D68 $3C^{pro}$. **A** and **B:** M^{pro} + **20g**; **C** and **D:** $3C^{pro}$ + **20g**; **E** and **F:** M^{pro} + **20e**; **G** and **H:** $3C^{pro}$ + **20e**.

37

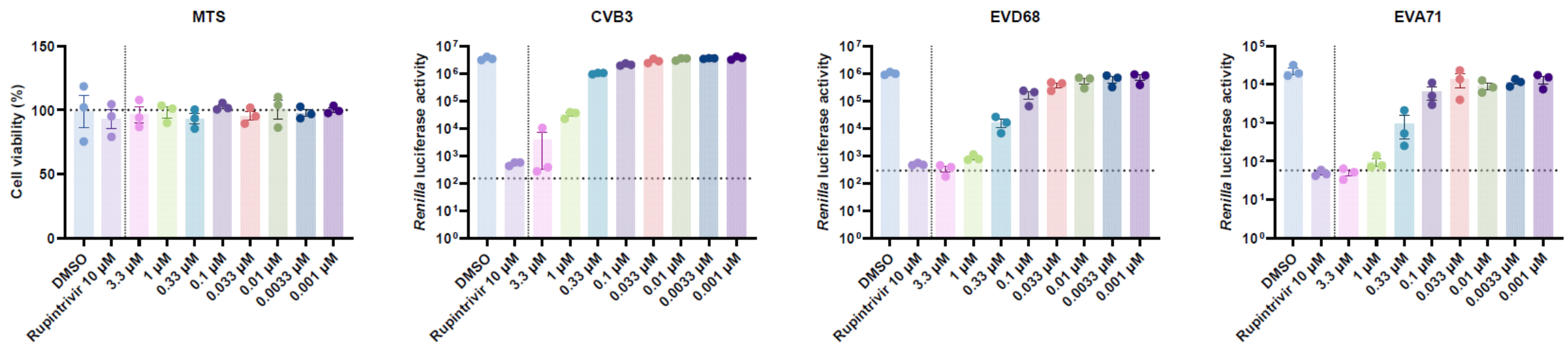

Figure S3. Antiviral effects of **20f** on EV-D68, EV-A71 and Coxsackievirus-B3. Antiviral effects were detected by the luciferase activity of corresponding reporter viruses, and effects on cell viability were measured using the MTS assay with Aqueous One Solution (Promega).

Intermediate synthesis

Preparation of methyl (S)-3-(3-allylphenyl)-2-((tert-butoxycarbonyl)amino)propanoate

To a solution of methyl (S)-3-(3-bromophenyl)-2-((tert-butoxycarbonyl) amino) propanoate (38 g, 0.106 mol) in toluene (230 mL) was added allyltributylstannane (49.5 mL, 53.1 g, 0.159 mol), and $Pd(Ph_3P)_4$ (9.2 g, 0.0079 mmol). The reaction mixture was heated at 100 °C overnight under a nitrogen atmosphere. The reaction mixture was added DCM (1 L), potassium fluoride (150 g) and 3 L of H_2O. Then the mixture was stirred for 1.5 h. The layers were split mad filtered the solids through CELITE. The organic phase was dried over $MgSO_4$, filtered mad concentrated. The concentrate was purified by flash chromatography to give the compound **5** (27 g, yield: 80%).

Table S1: Enzyme inhibitory activity of macrocyclic nitriles against SARS-CoV-2 M^{pro}.

Code	% inhibition at 20 µM	SD
**9a-H**	-6,56%	3,45%
**9a-K**	0,44%	1,48%
**9b-H**	-10,06%	1,85%
**9b-K**	61,96%	3,36%

SD: standard deviation

Table S2: Data collection and refinement statistics.

ID	SARS-Cov-2 M^{pro} + 20f	SARS-Cov-2 M^{pro} + 20g	SARS-Cov-2 M^{pro} + 20h	SARS-Cov-2 M^{pro} + 20a
**PDB code**	**9T5F**	**9SSO**	**9T72**	**9T5E**
**Wavelength (Å)**	1.0332	1.0332	1.0332	1.0332
**Resolution range**	45.97 - 1.68 (1.74 - 1.68)	49.38 - 1.6 (1.657 - 1.6)	48.58 - 1.7 (1.761 - 1.7)	49.3 - 1.69 (1.75 - 1.69)
**Space group**	*P 21 21 21*	*P 21 21 21*	*C 1 2 1*	*P 21 21 21*
**Unit cell**	67.567 100.472 103.406 90 90 90	67.793 100.178 103.791 90 90 90	114.361 53.938 44.709 90 102.038 90	67.666 100.11 103.52 90 90 90
**Total reflections**	1077061 (102358)	2480498 (215646)	200478 (20414)	1073249 (107738)
**Unique reflections**	80677 (7980)	93746 (9284)	29117 (2908)	79250 (7812)
**Multiplicity**	13.4 (12.8)	26.5 (23.2)	6.9 (7.0)	13.5 (13.8)
**Completeness (%)**	95.95 (99.85)	99.94 (99.92)	98.88 (99.66)	99.76 (99.92)
**Mean I/sigma(I)**	14.42 (1.14)	16.50 (1.01)	15.95 (1.43)	13.44 (1.18)
**Wilson B-factor**	20.80	22.80	34.36	22.19
**R-merge**	0.1497 (2.302)	0.1453 (3.39)	0.0536 (1.066)	0.1415 (2.603)
**R-meas**	0.1557 (2.397)	0.1482 (3.465)	0.05801 (1.151)	0.1471 (2.703)
**R-pim**	0.04248 (0.6611)	0.02871 (0.7076)	0.02192 (0.4307)	0.03979 (0.7228)
**CC1/2**	0.999 (0.636)	0.999 (0.567)	0.999 (0.704)	0.999 (0.595)

**CC***	1 (0.882)	1 (0.851)	1 (0.909)	1 (0.864)
**Reflections used in refinement**	77569 (7977)	93737 (9284)	29105 (2908)	79238 (7812)
**Reflections used for R-free**	3837 (386)	4652 (486)	1419 (140)	3903 (369)
**R-work**	0.2343 (0.3585)	0.2004 (0.3428)	0.1947 (0.2942)	0.2129 (0.3484)
**R-free**	0.2561 (0.3696)	0.2290 (0.3624)	0.2350 (0.3263)	0.2388 (0.3533)
**CC(work)**	0.935 (0.458)	0.954 (0.522)	0.960 (0.680)	0.948 (0.481)
**CC(free)**	0.919 (0.351)	0.938 (0.510)	0.940 (0.603)	0.947 (0.395)
**Number of non-hydrogen atoms**	4936	5054	2509	5061
**macromolecules**	4691	4707	2376	4673
**ligands**	90	78	41	78
**solvent**	155	269	92	310
**Protein residues**	607	609	306	605
**RMS(bonds)**	0.009	0.010	0.009	0.009
**RMS(angles)**	1.61	1.65	1.65	1.58
**Ramachandran favored (%)**	97.51	98.18	97.37	98.17
**Ramachandran allowed (%)**	2.49	1.82	2.30	1.66

**Ramachandran outliers (%)**	0.00	0.00	0.33	0.17
**Rotamer outliers (%)**	1.54	1.91	1.14	1.93
**Clashscore**	3.31	2.45	3.59	3.97
**Average B-factor**	29.08	30.20	40.83	30.32
**macromolecules**	29.05	29.93	40.45	29.49
**ligands**	33.56	33.35	61.35	69.07
**solvent**	27.49	34.06	41.64	33.12

Table S3. Data collection and refinement statistics.

ID	SARS-Cov-2 M^{pro} + 9b-K	SARS-Cov-2 M^{pro} + 20b	SARS-Cov-2 M^{pro} + 20e	D68-$3C^{pro}$ + 20e	D68-$3C^{pro}$ + 20g
**PDB code**	**9SSN**	**9T52**	**9T55**	**29CT**	**29DA**
**Wavelength (Å)**	1.0332	1.0332	1.0332	1.0332	1.0332
**Resolution range**	43.2 - 1.5 (1.554 - 1.5)	46.05 - 1.76 (1.823 - 1.76)	48.11 - 1.75 (1.813 - 1.75)	46.98 - 1.91 (1.978 - 1.91)	48.68 - 2.08 (2.154 - 2.08)
**Space group**	*C 1 2 1*	*P 1 21 1*	*C 1 2 1*	*P 31 2 1*	*P 31 2 1*
**Unit cell**	98.093 80.382 51.635 90 114.341 90	67.252 123.422 71.582 90 104.923 90	113.367 53.438 44.486 90 102.913 90	56.43 56.43 170.57 90 90 120	56.21 56.21 170.66 90 90 120
**Total reflections**	222486 (15932)	1547402 (156260)	177251 (18147)	250987 (25535)	192664 (20034)

**Unique reflections**	56677 (4745)	110393 (11066)	25986 (2584)	25274 (2358)	19582 (1930)
**Multiplicity**	3.9 (3.4)	14.0 (14.1)	6.8 (7.0)	9.9 (10.5)	9.8 (10.4)
**Completeness (%)**	97.05 (81.42)	99.05 (99.70)	98.65 (98.97)	99.47 (96.44)	99.87 (99.90)
**Mean I/sigma(I)**	28.70 (4.29)	16.32 (1.65)	14.21 (2.62)	11.53 (0.32)	15.07 (0.86)
**Wilson B-factor**	17.45	25.33	22.09	55.88	55.56
**R-merge**	0.02324 (0.2268)	0.1053 (1.879)	0.07987 (0.7228)	0.08552 (11.1)	0.08814 (2.435)
**R-meas**	0.02678 (0.2704)	0.1093 (1.949)	0.08666 (0.781)	0.09055 (11.67)	0.09323 (2.563)
**R-pim**	0.01308 (0.1433)	0.02886 (0.5131)	0.0332 (0.2926)	0.0292 (3.566)	0.02991 (0.7916)
**CC1/2**	1 (0.976)	0.999 (0.75)	0.998 (0.893)	0.999 (0.323)	0.999 (0.674)
**CC***	1 (0.994)	1 (0.926)	1 (0.971)	1 (0.699)	1 (0.898)
**Reflections used in refinement**	56671 (4745)	110383 (11066)	25964 (2584)	25199 (2358)	19580 (1931)
**Reflections used for R-free**	2850 (243)	5645 (562)	1306 (114)	1261 (119)	980 (97)
**R-work**	0.1849 (0.2583)	0.1843 (0.3191)	0.1847 (0.2613)	0.2337 (0.6230)	0.2113 (0.4095)
**R-free**	0.2101 (0.2456)	0.2191 (0.3598)	0.2325 (0.3066)	0.2713 (0.6134)	0.2395 (0.4348)
**CC (work)**	0.953 (0.560)	0.958 (0.574)	0.958 (0.653)	0.904 (0.198)	0.923 (0.420)
**CC (free)**	0.943 (0.606)	0.949 (0.517)	0.930 (0.459)	0.884 (0.046)	0.939 (0.395)
**Number of non-hydrogen atoms**	2618	10193	2557	1526	1566
**Macromolecules**	2335	9400	2358	1469	1469
**ligands**	31	160	41	41	39
**solvent**	252	633	158	16	58
**Protein residues**	301	1216	305	189	189
**RMS (bonds)**	0.012	0.008	0.009	0.010	0.009

**RMS (angles)**	1.81	1.52	1.63	1.97	1.90
**Ramachandran favored (%)**	98.33	98.68	98.68	95.70	95.70
**Ramachandran allowed (%)**	1.67	1.24	0.99	4.30	4.30
**Ramachandran outliers (%)**	0.00	0.08	0.33	0.00	0.00
**Rotamer outliers (%)**	1.92	1.82	0.76	5.70	4.43
**Clashscore**	2.37	2.83	3.82	4.06	3.73
**Average B-factor**	22.81	32.00	27.49	78.97	64.17
**Macromolecules**	21.98	31.52	26.91	78.40	63.19
**ligands**	21.45	36.58	42.88	98.77	95.87
**solvent**	30.70	38.03	32.13	80.50	67.52

[a] The highest resolution shell is shown in parentheses.

[b] $R_{merge} = \sum_{hkl}\sum_{i=1}^{n}\left|I_i(hkl) - \bar{I}(hkl)\right| / \sum_{hkl}\sum_{i=1}^{n} I_i(hkl)$

[c] $R_{pim} = \sum_{hkl}\sqrt{1/(n-1)}\sum_{i=1}^{n}\left|I_i(hkl) - \bar{I}(hkl)\right| / \sum_{hkl}\sum_{i=1}^{n} I_i(hkl)$[1]

[d] $CC_{1/2}$ is the correlation coefficient determined by two random half data sets.[2]

[e] $R_{cryst} = \sum_{hkl}|F_o(hkl) - F_c(hkl)| / \sum_{hkl}|F_o(hkl)|$

[f] R_{free} was calculated for a test set of reflections (5%) omitted from the refinement.

[g] Clashscore is defined as the number of clashes calculated for the model per 1000 atoms (including hydrogens) of the model. Hydrogens were added by MolProbity.[3]

Table S4. Mass transitions for cellular accumulation and ADME assessments.[a)]

ID	Q1 [Da]	Q3 [Da]	DP [volts]	CE [volts]	CXP [volts]
**caffeine**	195.0	138.0	80.0	25.0	14.0
		110.0	80.0	31.0	18.0
**20b**	547.3	384.2	26.0	43.0	16.0
		528.9	26.0	25.0	28.0
**20e**	561.3	384.1	1.0	33.0	20.0
		366.1	1.0	43.0	12.0
**20f**	638.3	384.2	1.0	51.0	22.0
		620.5	1.0	31.0	32.0
**20g**	554.3	411.1	86.0	27.0	24.0
		384.1	86.0	29.0	42.0
	576.3	451.2	256.0	45.0	20.0
		558.2	256.0	45.0	26.0
**20h**	559.2	481.4	101.0	27.0	2.0
		382.2	101.0	35.0	2.0

a) Measurements on an AB SCIEX QTrap 6500plus mass spectrometer.

Table S5. Mass transitions for cellular accumulation experiments.[a)]

ID	Q1 [Da]	Q3 [Da]	DP [volts]	CE [volts]	CXP [volts]
**caffeine**	195.1	100.9	10.0	12.0	10.0
		116.9	10.0	10.0	7.0
**20b**	545.3	448.2	-10.0	-26.0	-42.0
		312.2	-10.0	-56.0	-43.0
		330.2	-10.0	-34.0	-43.0
**20e**	559.3	462.2	-10.0	-32.0	-43.0
		42.0	-10.0	-138.0	-19.0
		312.2	-10.0	-53.0	-19.0
**20f**	636.3	155.1	-10.0	-52.0	-14.0
		469.3	-10.0	-39.0	-44.0
		98.1	-10.0	-107.0	-10.0
**20g**	552.3	312.2	-10.0	-50.0	-23.0
		330.2	-10.0	-34.0	-10.0
		427.2	-10.0	-25.0	-6.0
**20h**	557.3	460.2	-10.0	-28.0	-36.0
		539.3	-10.0	-28.0	-36.0
		283.1	-10.0	-50.0	-36.0

a) Measurements on an AB SCIEX Triple QuadTM 7500 mass spectrometer.

Table S6. Cellular accumulation data.

	$K_{p[37\ °C]}$ [a)]	$K_{p[4\ °C]}$ [a)]	$K_{p,final}$ [b)]
**20b**	3.98 ± 2.1	1.56 ± 0.3	2.42
**20e**	6.53 ± 1.3	2.96 ± 1.1	3.57
**20f**	3.10 ± 1.6	1.43 ± 0.4	1.67
**20g**	2.61 ± 0.9	1.76 ± 1.8	0.85
**20h**	5.64 ± 2.7	2.14 ± 0.2	3.50

a) mean of two to four biological replicate measurements with three technical replicates each ± SD

b) $K_{p,final} = K_{p[37\ °C]} - K_{p[4\ °C]}$

Table S7. Enzymatic inhibition of $3C^{pro}$ of EV-A71 by macrocycles **20b** and **20e.**

Cmpd	EV-A71 $3C^{pro}$ IC_{50}[1] (µM)
**20b**	6.9 ± 1.7
**20e**	4.4 ± 0.5

[1] ± Standard deviation (SD), n = 3.

Table S8. Inhibition of human cathepsins by selected macrocycles at concentrations of 1 and 10 µM**.**

	Cathepsin L		Cathepsin B		Cathepsin K	
	10 µM	1 µM	10 µM	1 µM	10 µM	1 µM
**Cmpd**						
**20b**	86	75	7	2	47	8
**20e**	84	70	-4	-9	43	7
**20h**	96	87	94	48	89	57

Figure S4. ^{1}H-NMR and ^{13}C-NMR spectra

^{1}H-NMR (700 MHz, CD_3OD) of 9a-H.

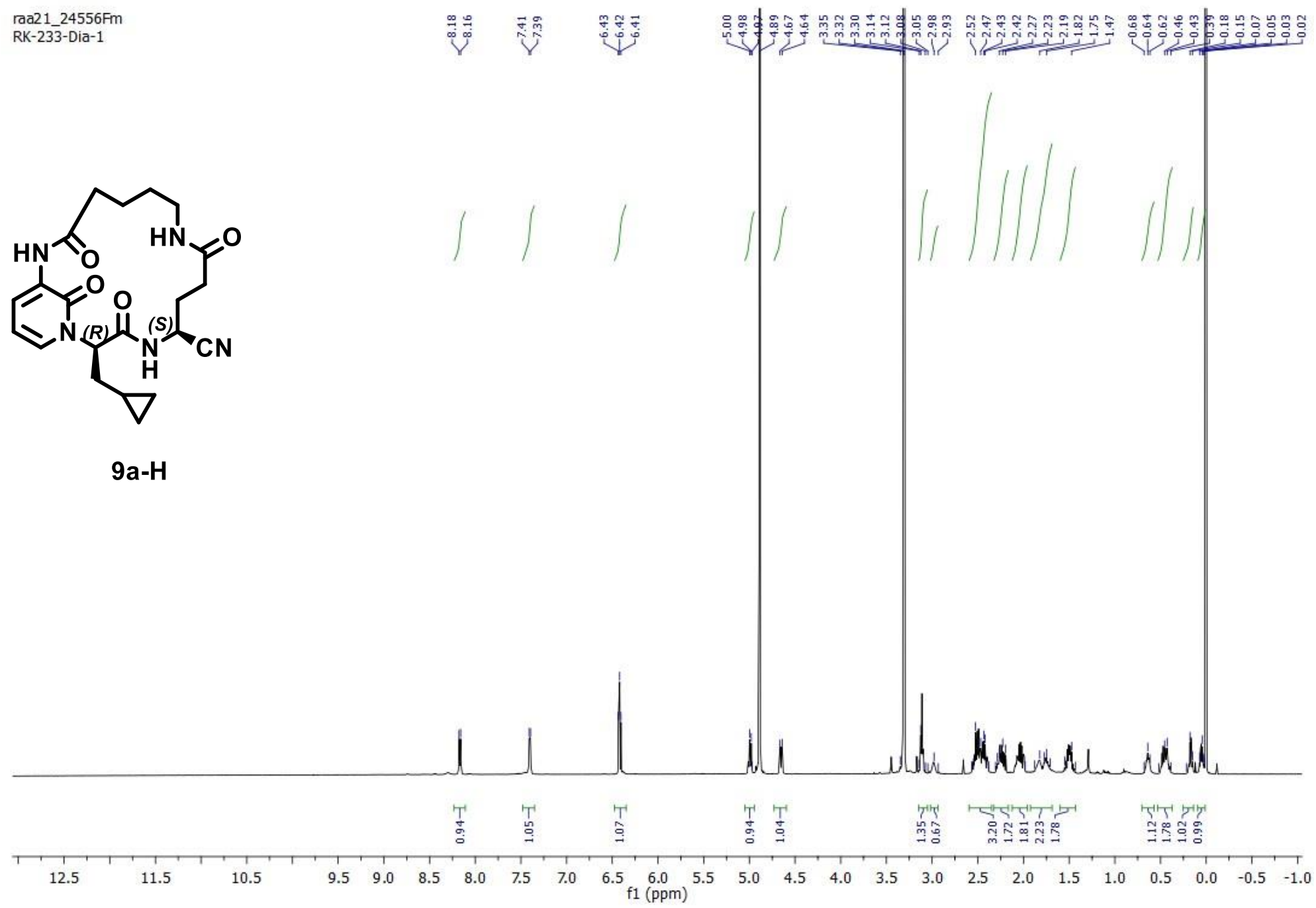

^{13}C-NMR (125 MHz, CD_3OD) of 9a-H.

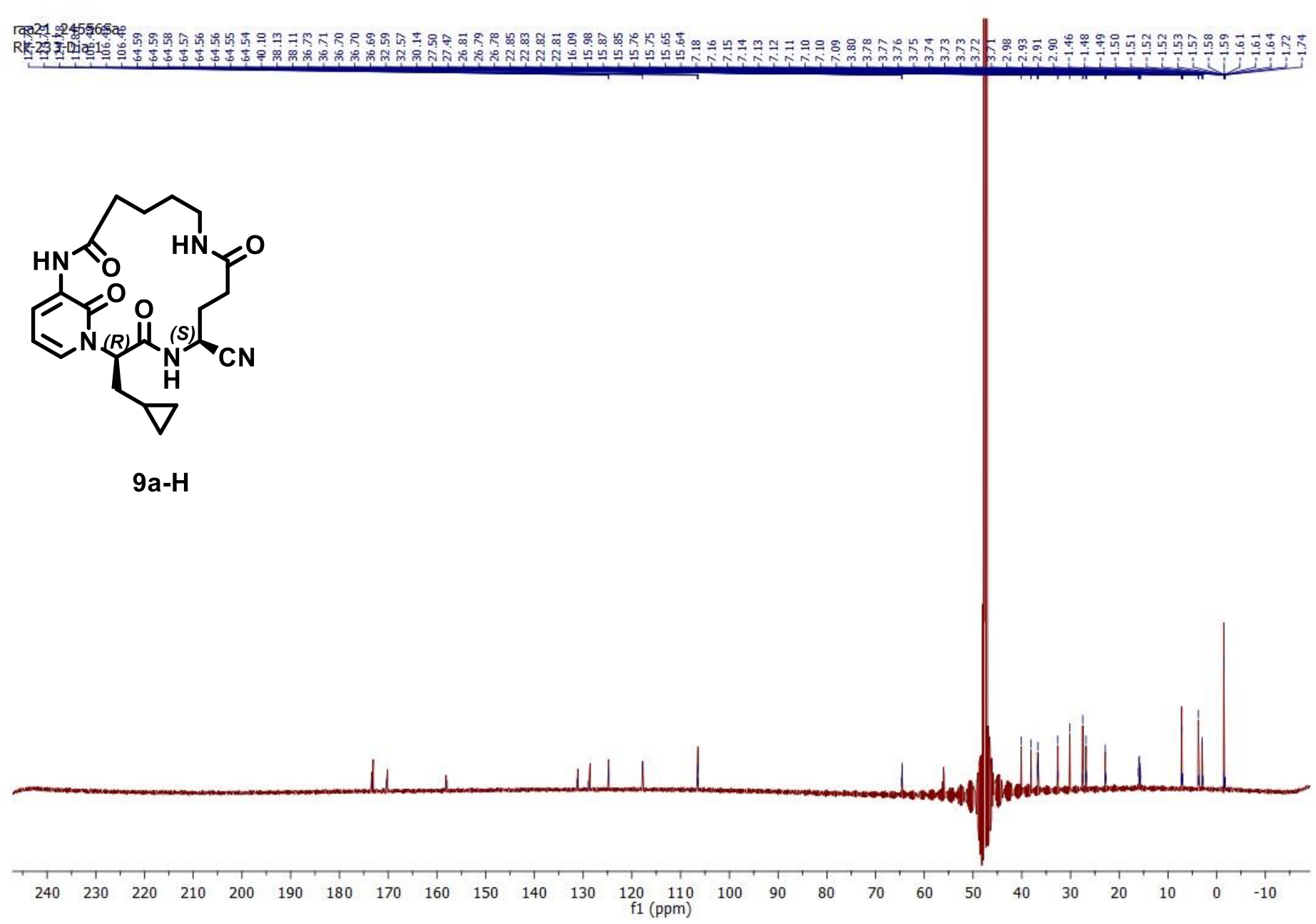

^{1}H-NMR (700 MHz, CD_3OD) of 9a-K.

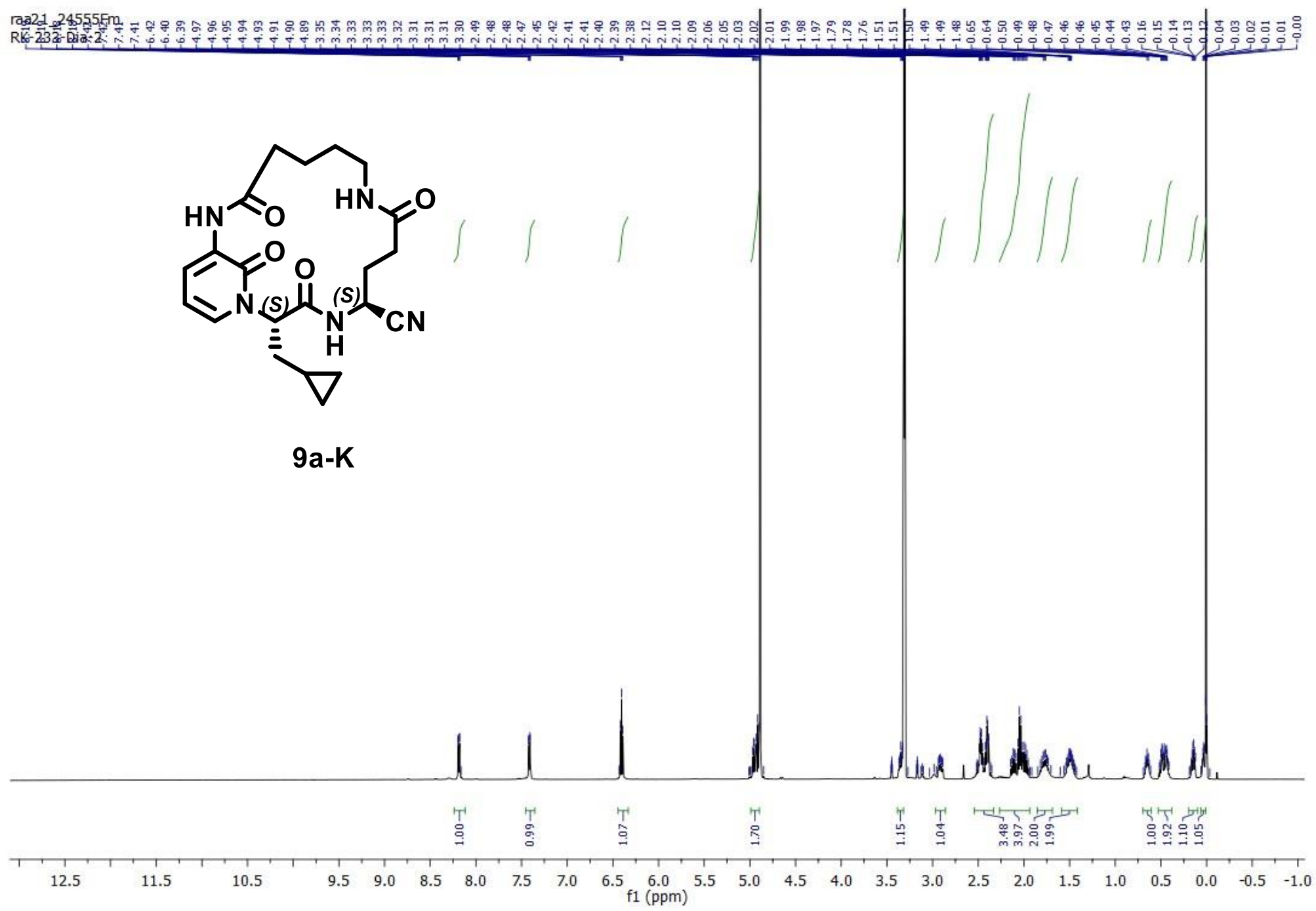

^{13}C-NMR (125 MHz, CD_3OD) of 9a-K.

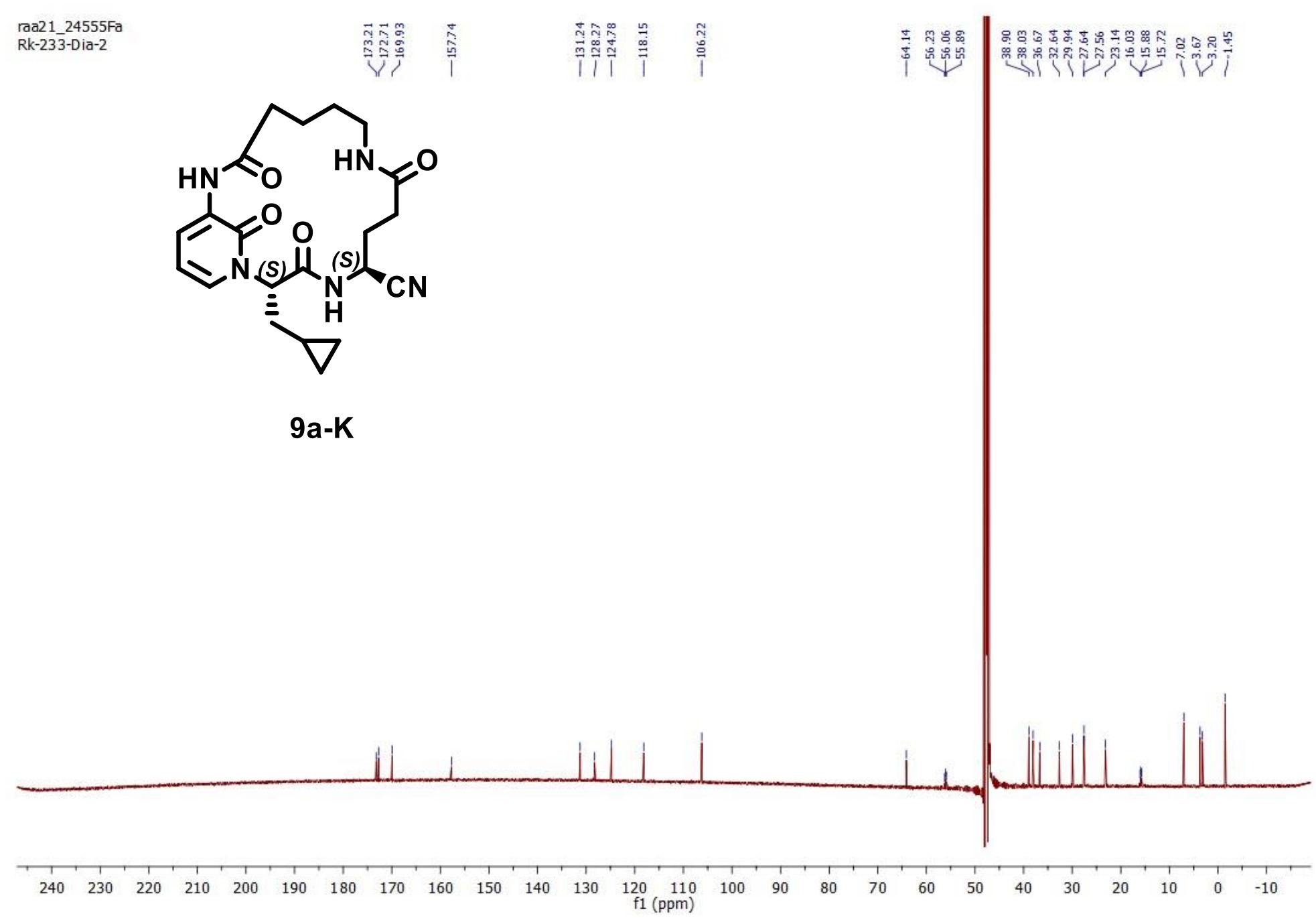

^{1}H-NMR (700 MHz, CD_3OD) of 9b-H.

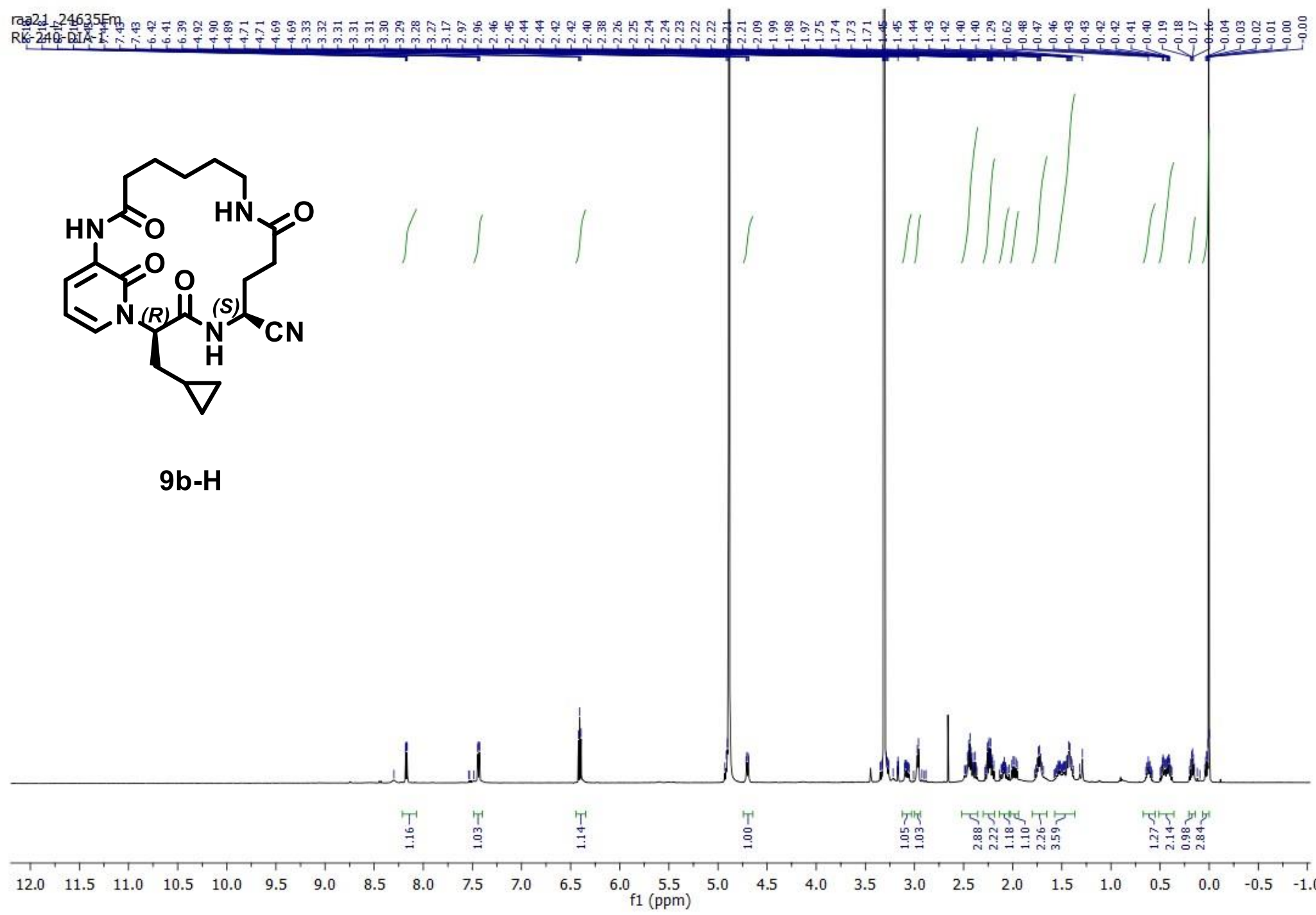

^{13}C-NMR (125 MHz, CD_3OD) of 9b-H.

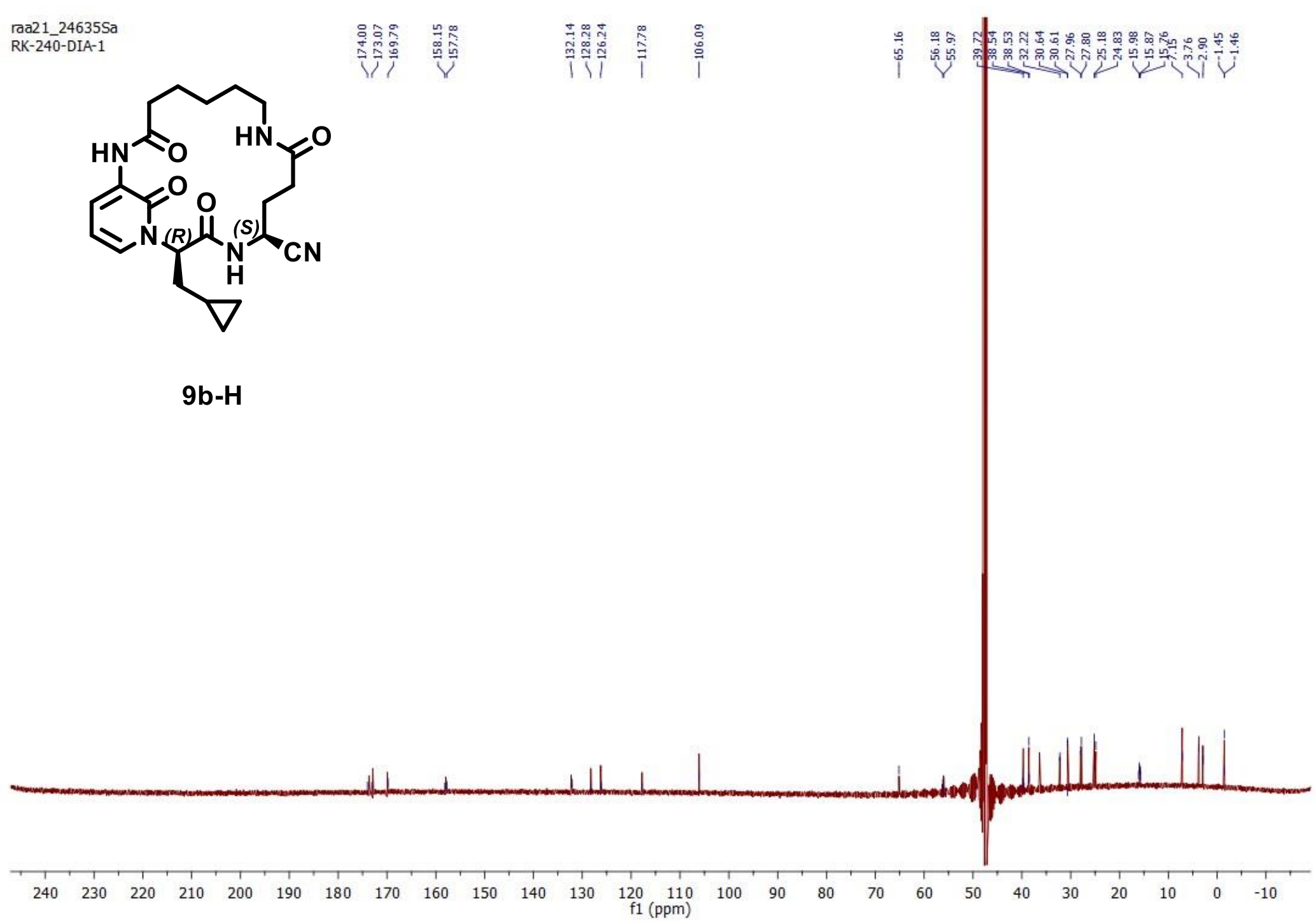

^{1}H-NMR (700 MHz, CD_3OD) of 9b-K.

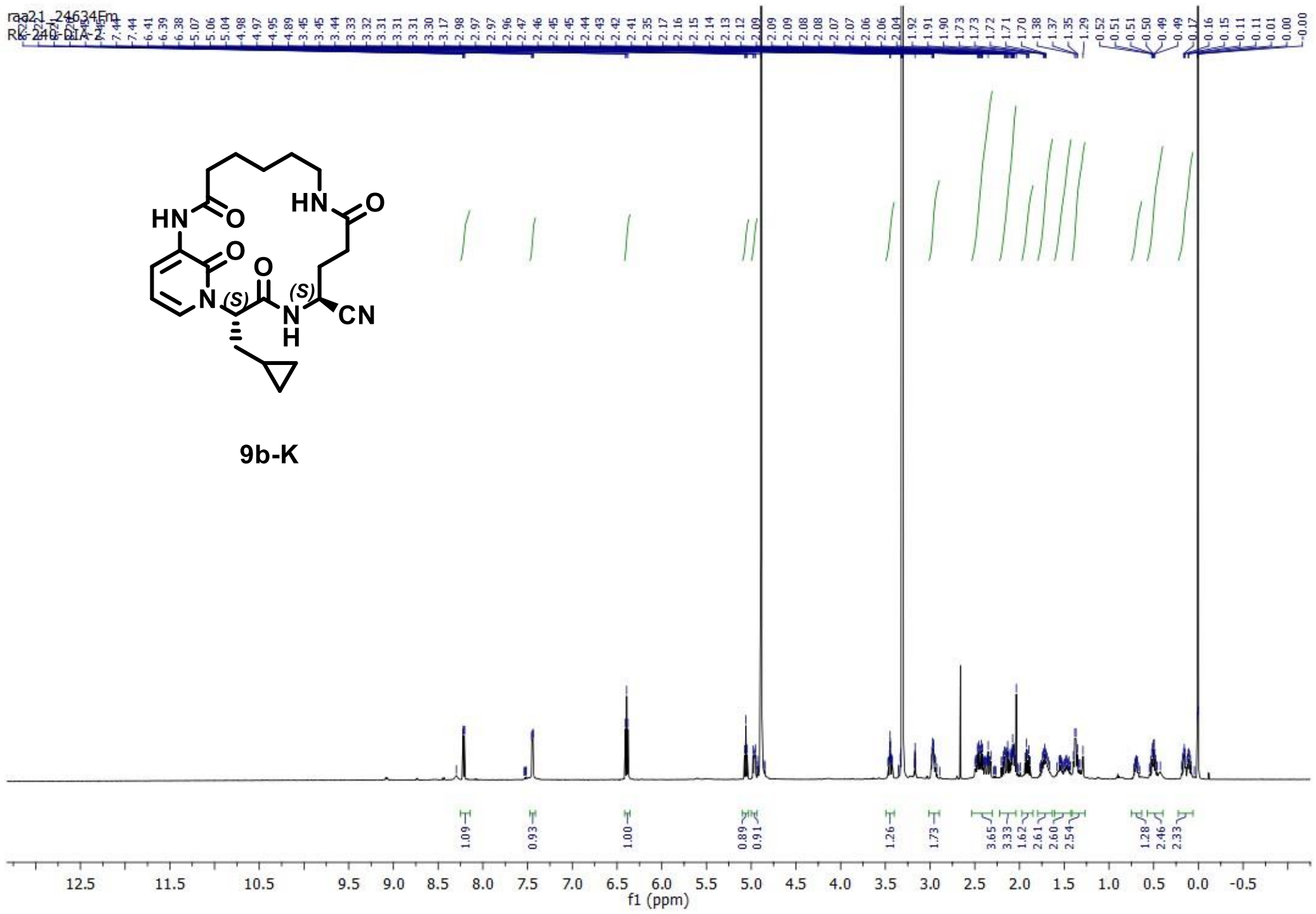

^{13}C-NMR (125 MHz, CD_3OD) of 9b-K.

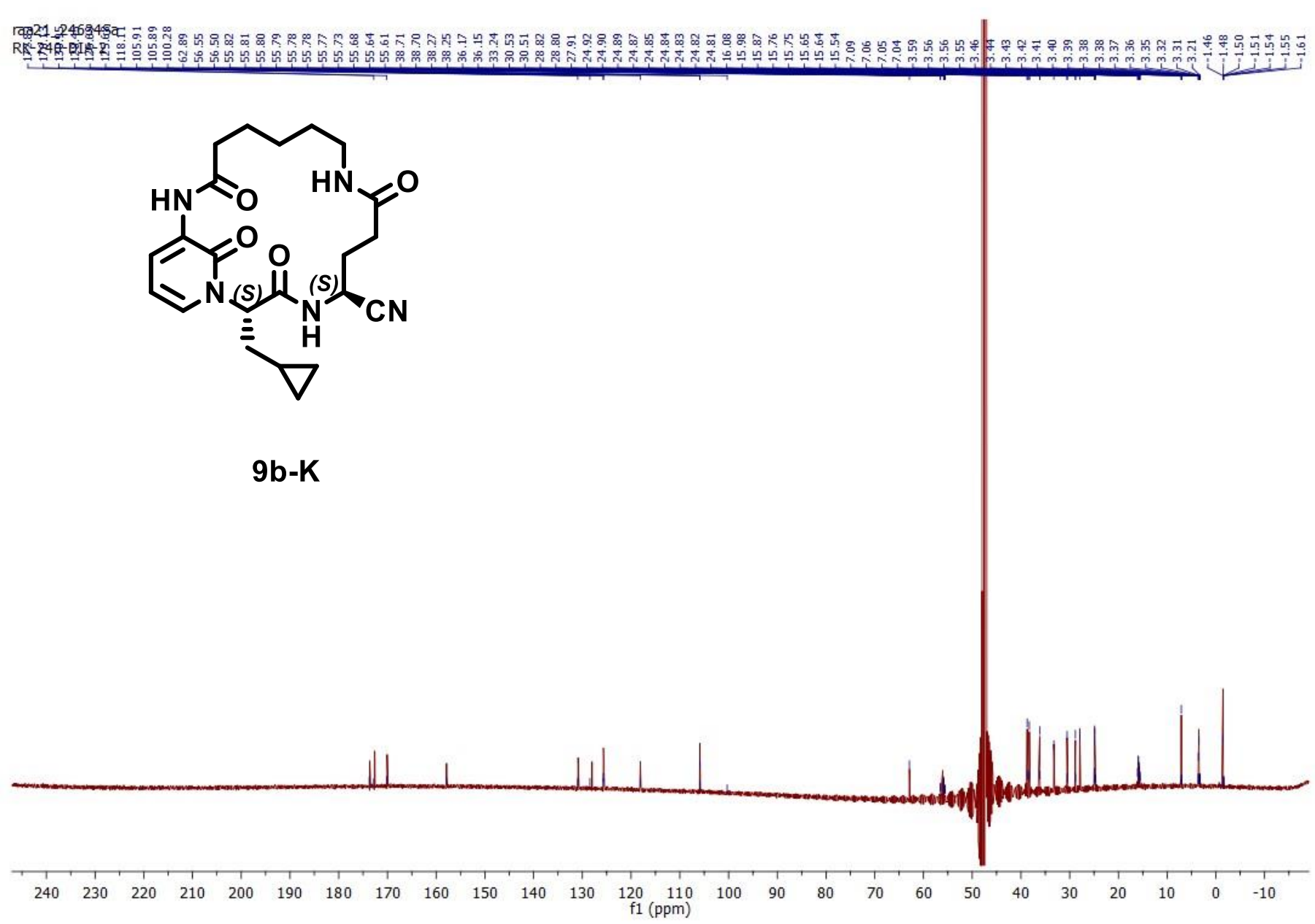

Zoomed-in regions of ^{1}H-NMR spectra of 9a-H & 9a-K; 9b-H & 9b-K in CD_3OD.

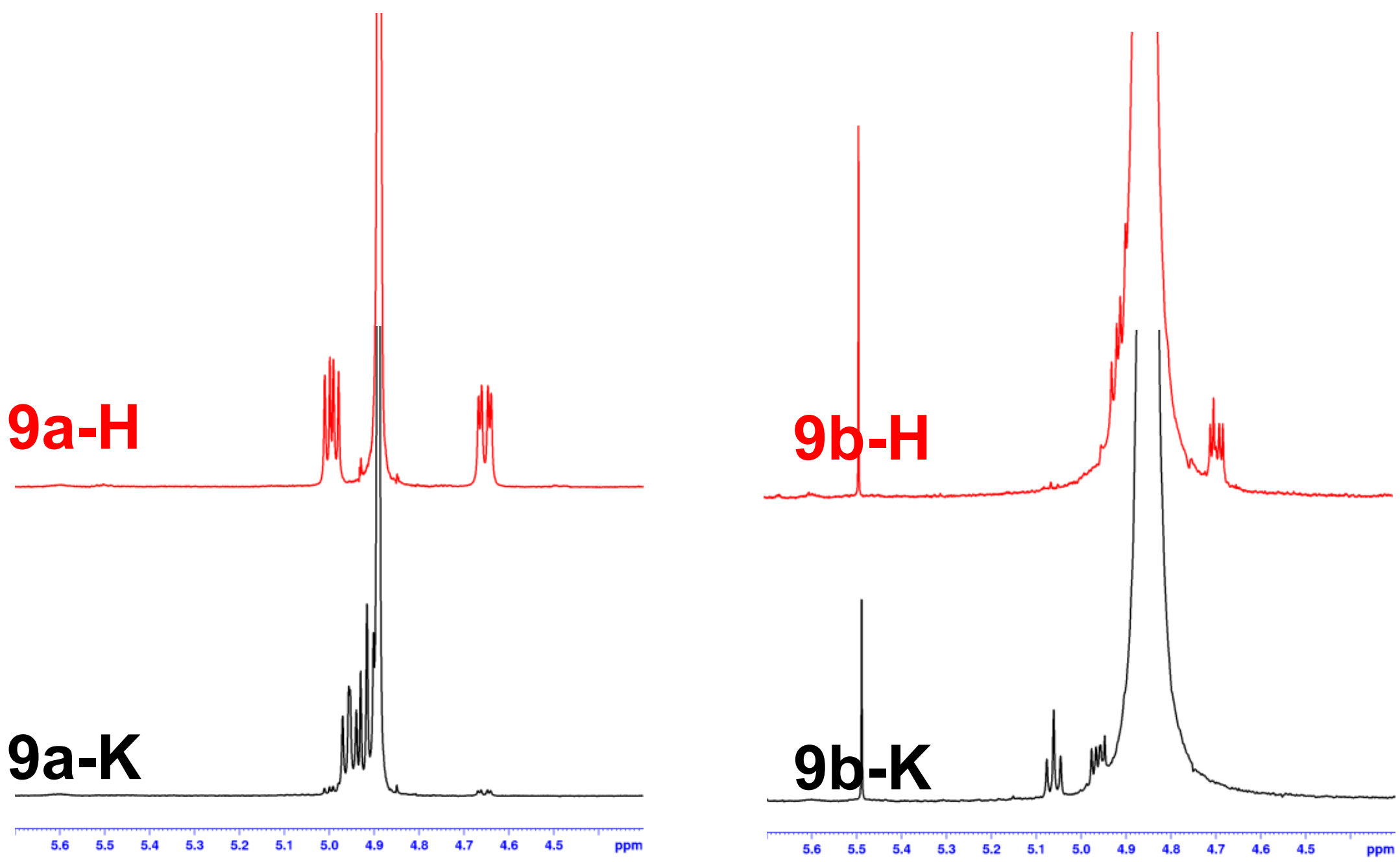

^{1}H-NMR (700 MHz, DMSO-d_6) of 20a.

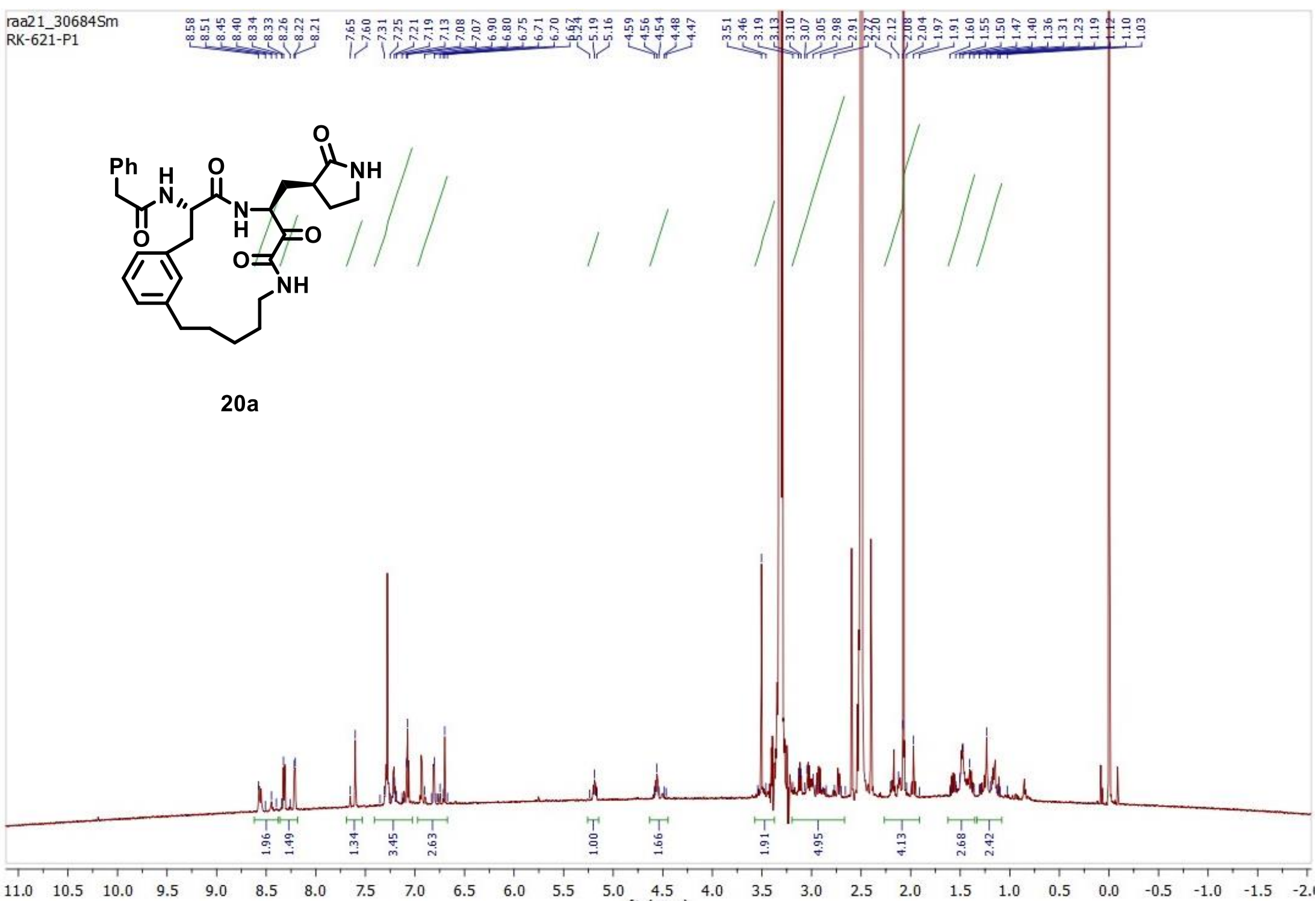

^{13}C-NMR (176 MHz, DMSO-d_6, DEPT-135) of 20a.

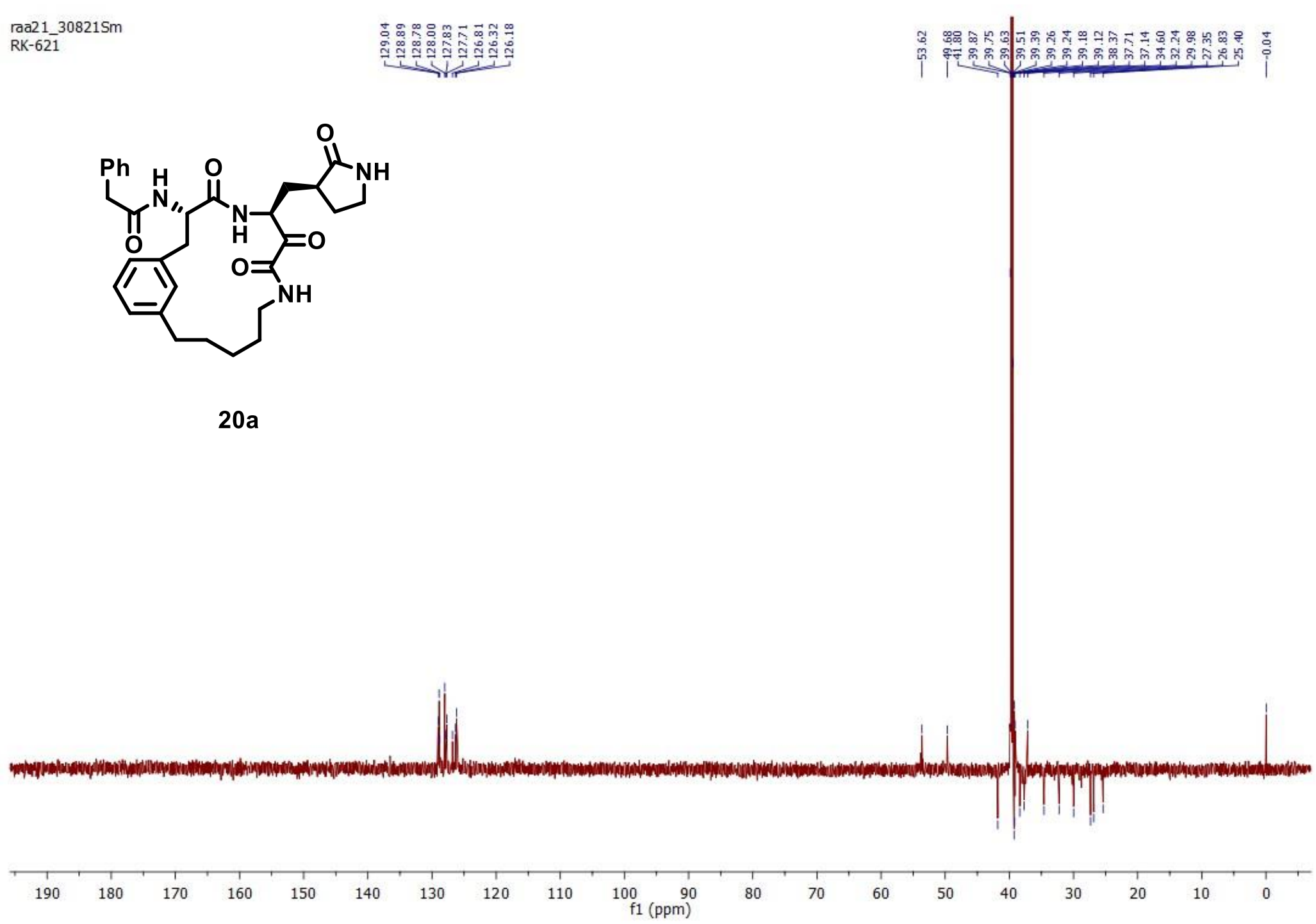

^{1}H-NMR (700 MHz, DMSO-d_6) of 20b.

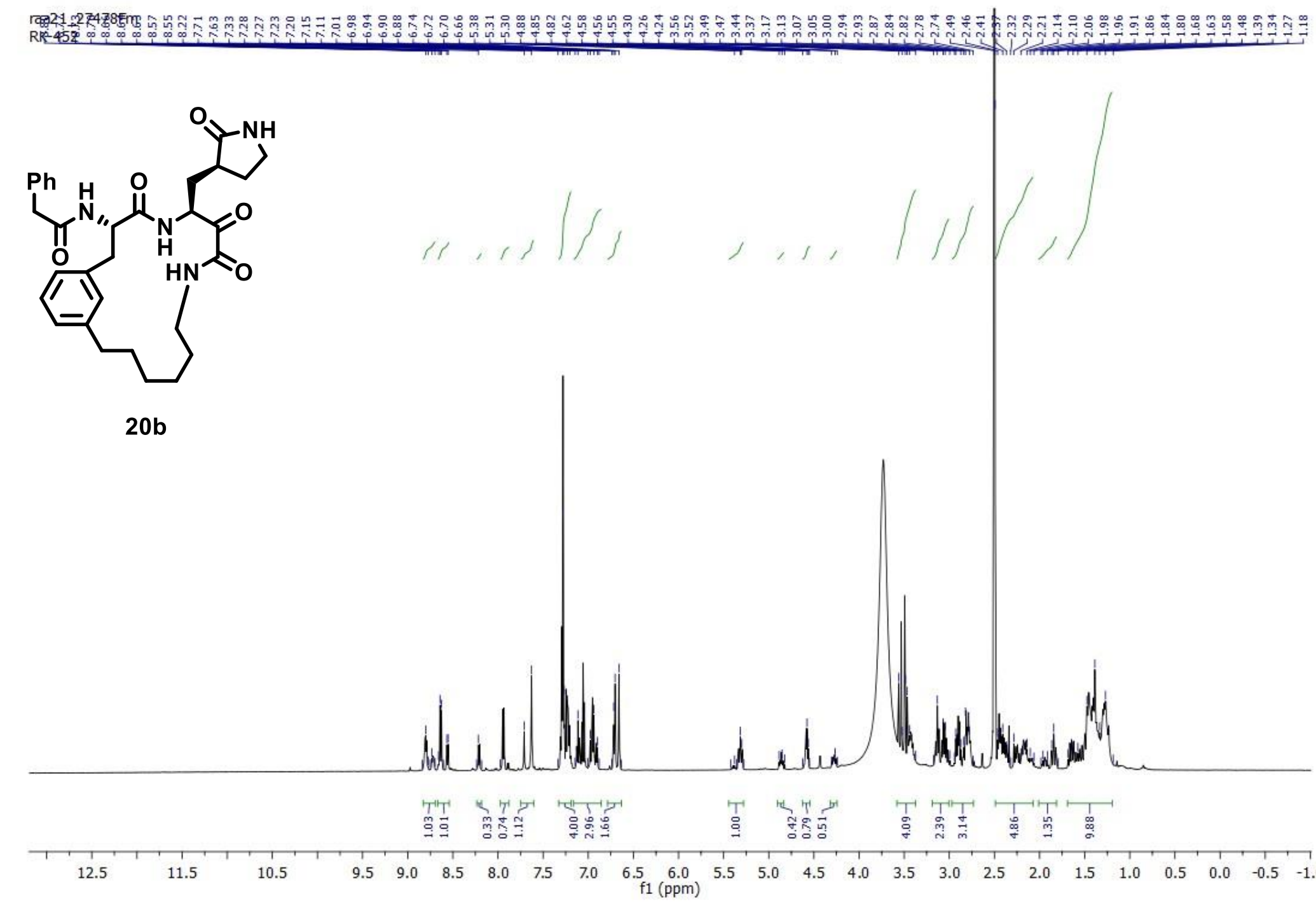

^{13}C-NMR (125 MHz, DMSO-d_6) of 20b.

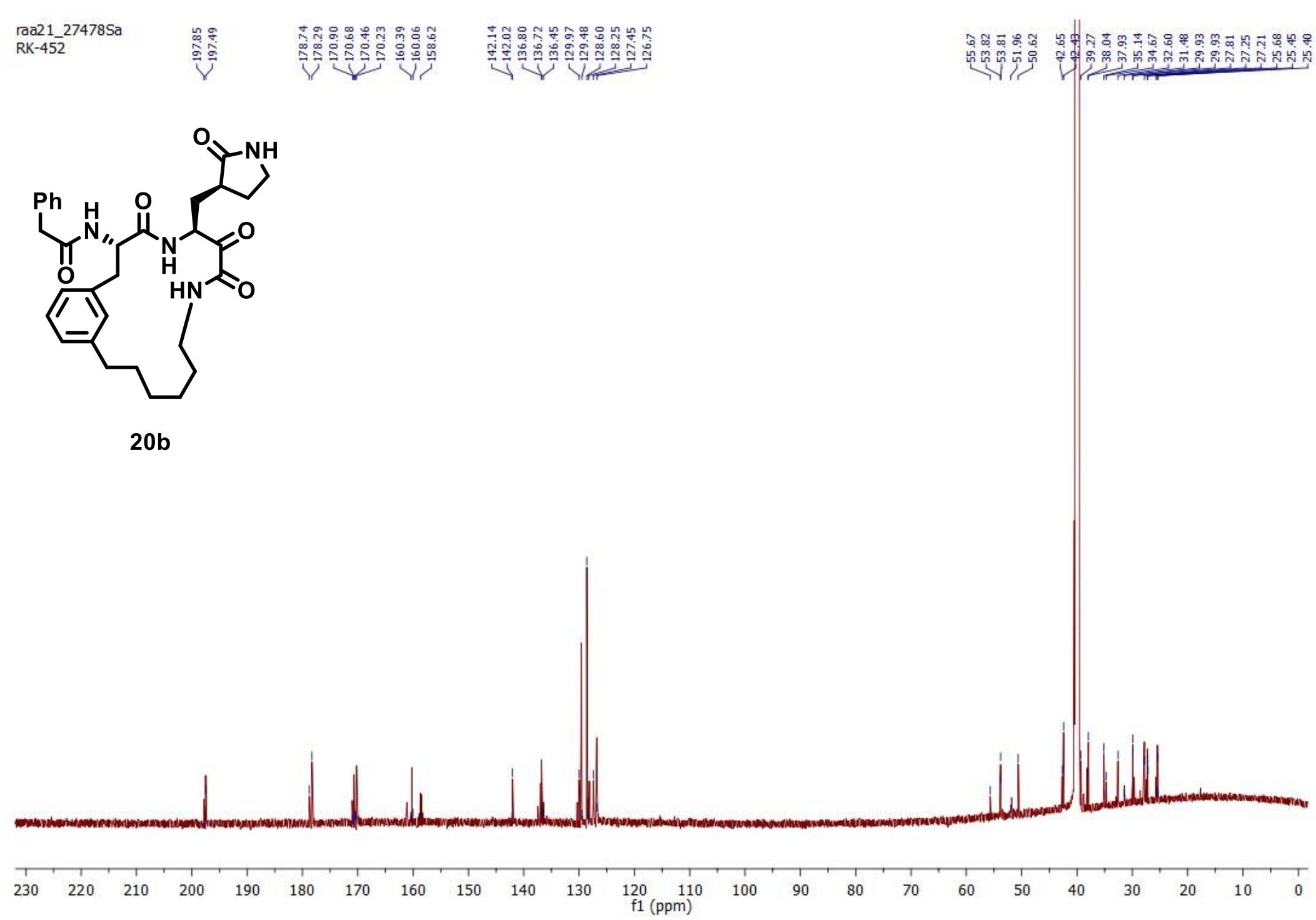

^{1}H-NMR (700 MHz, DMSO-d_6) of 20c.

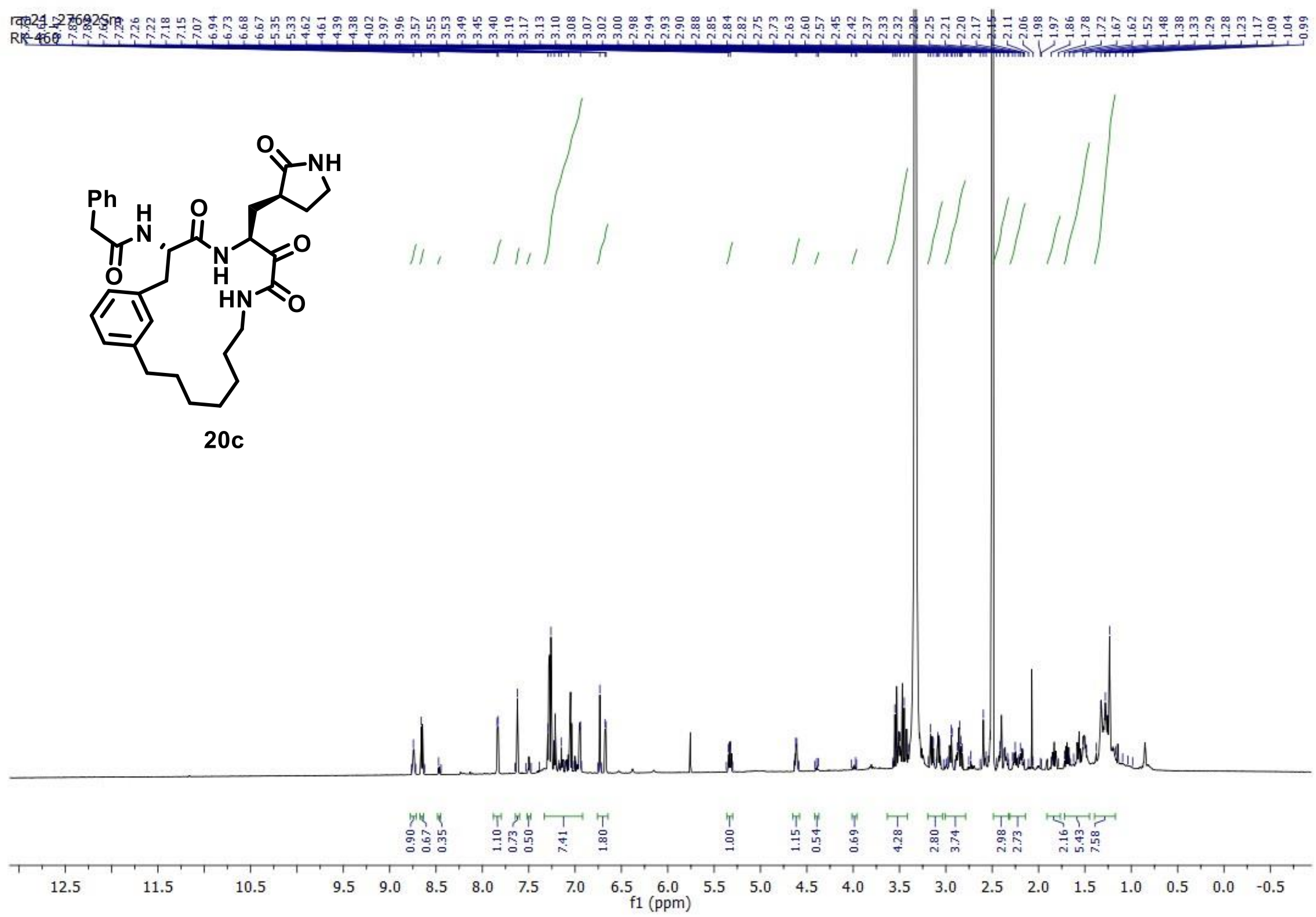

^{13}C-NMR (125 MHz, DMSO-d_6, DEPT-Q) of 20c.

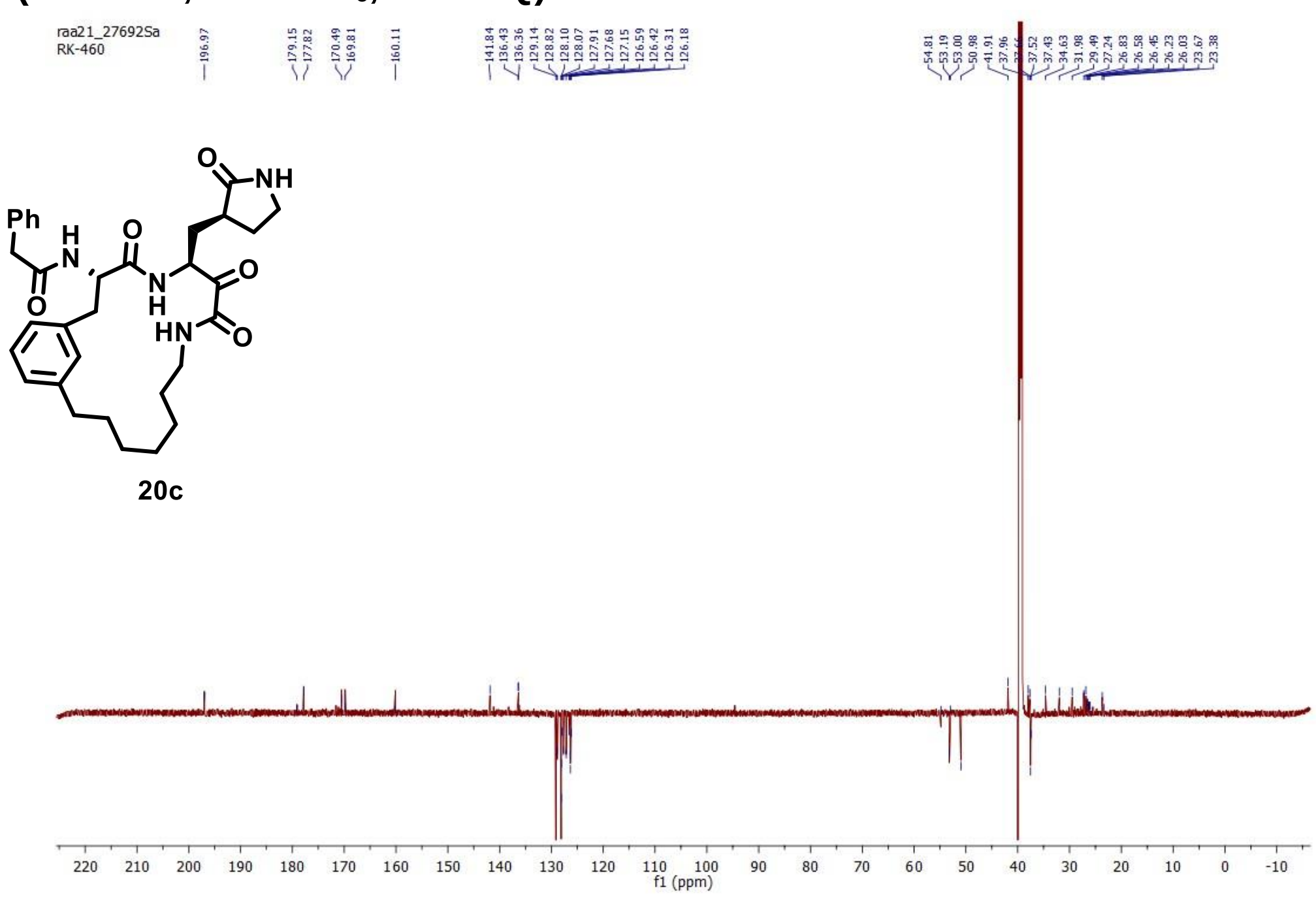

^{1}H-NMR (700 MHz, DMSO-d_6) of 20d.

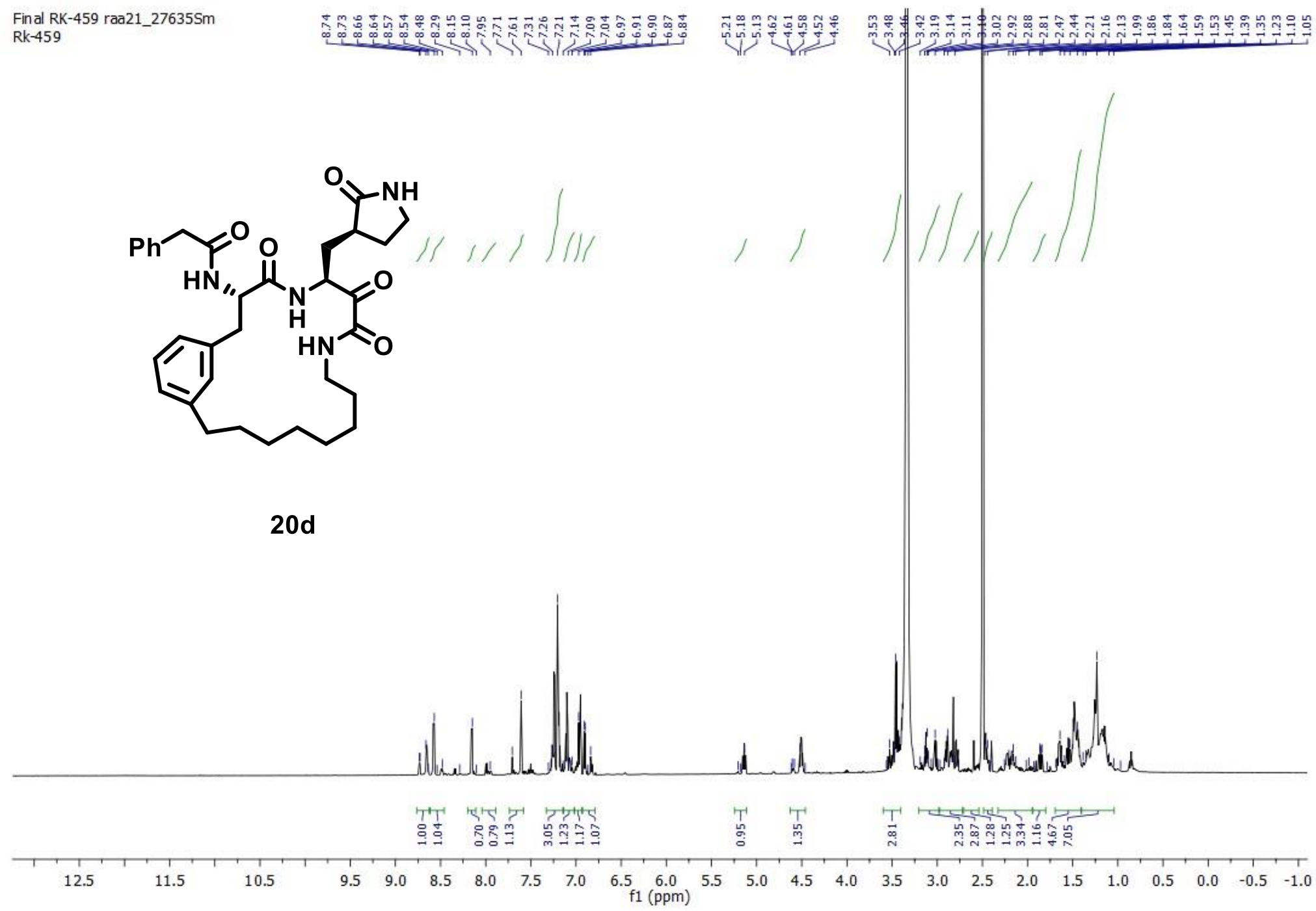

^{13}C-NMR (125 MHz, DMSO-d_6) of 20d.

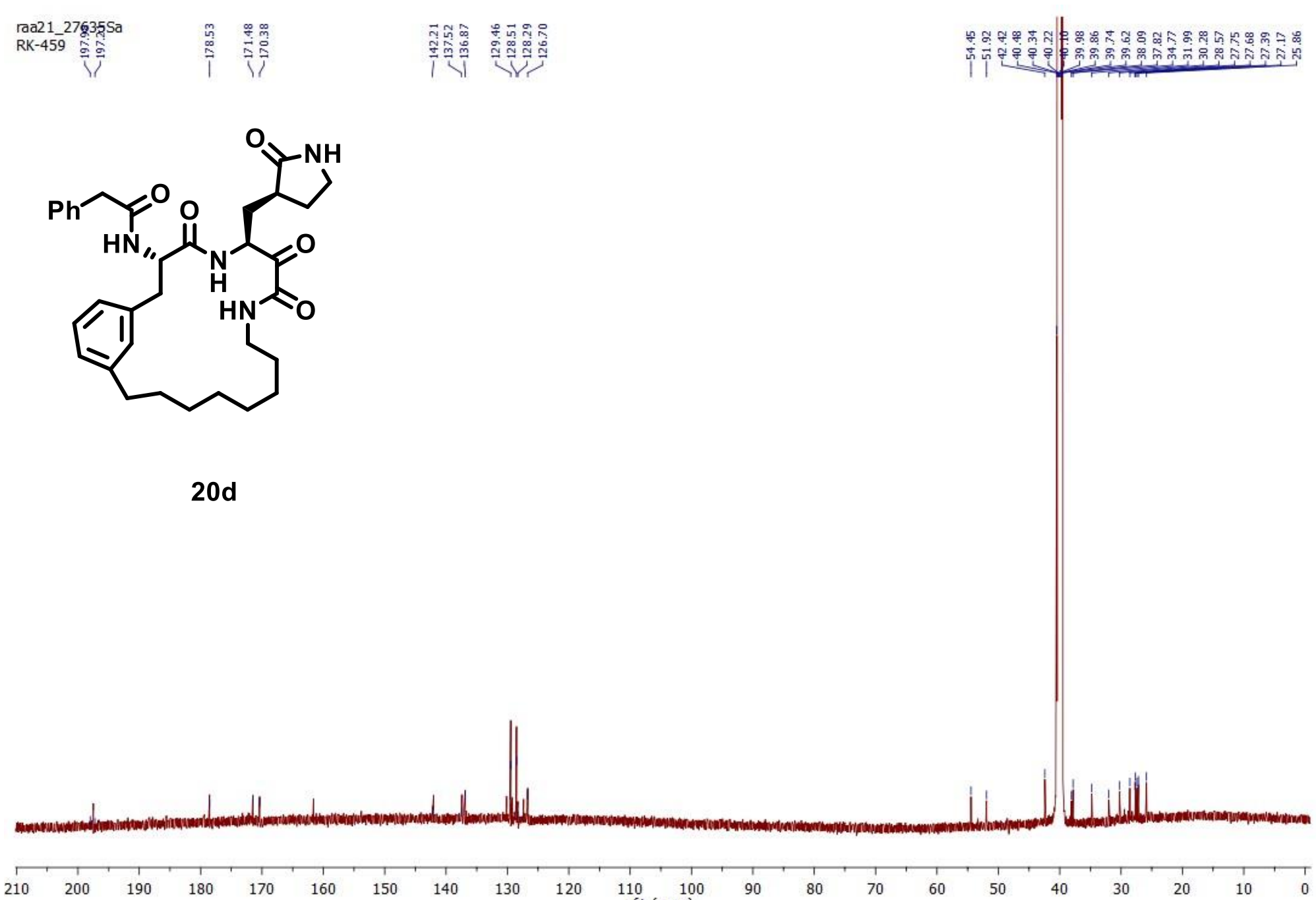

^{1}H-NMR (700 MHz, DMSO-d_6) of 20e.

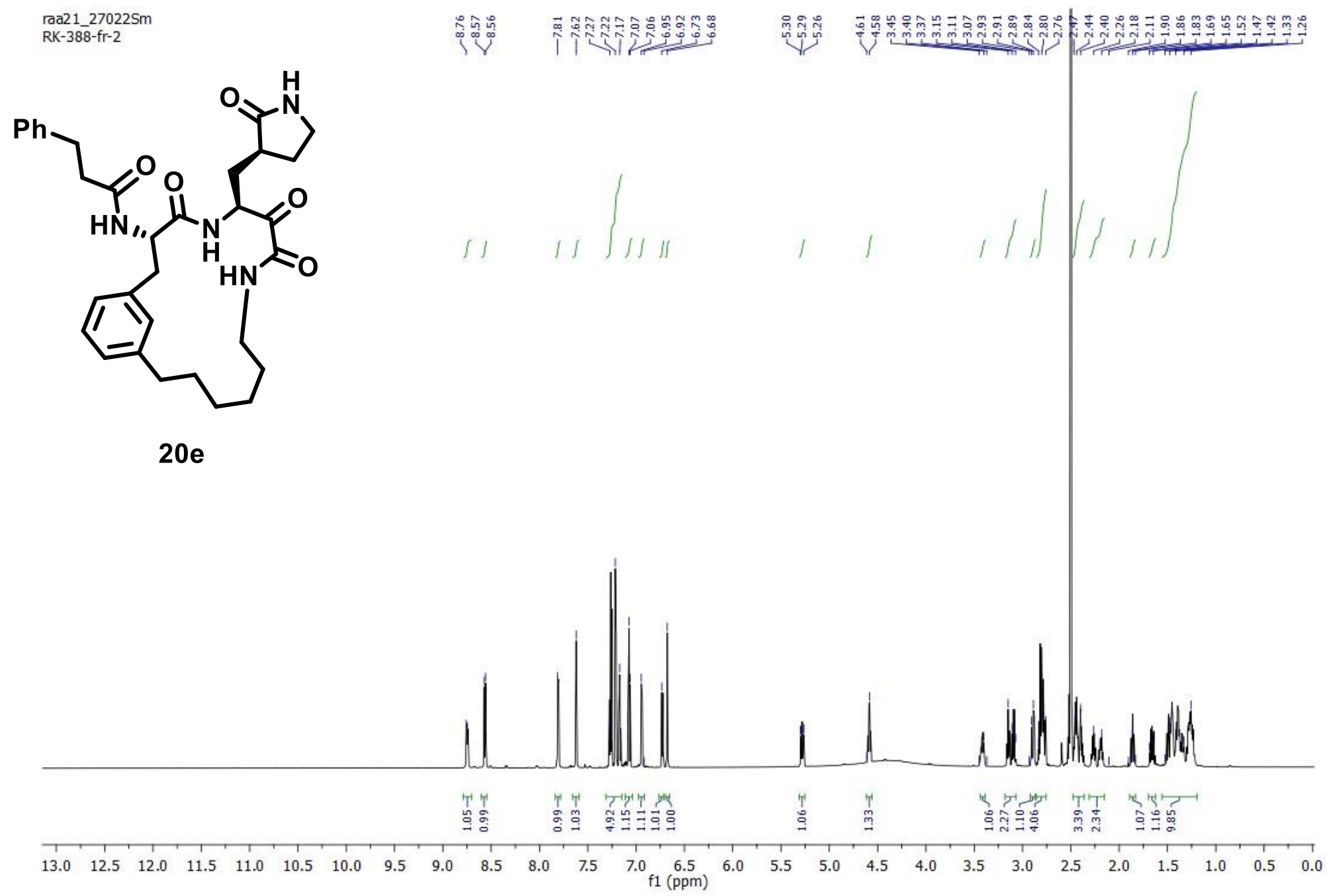

^{13}C-NMR (125 MHz, DMSO-d_6) of 20e.

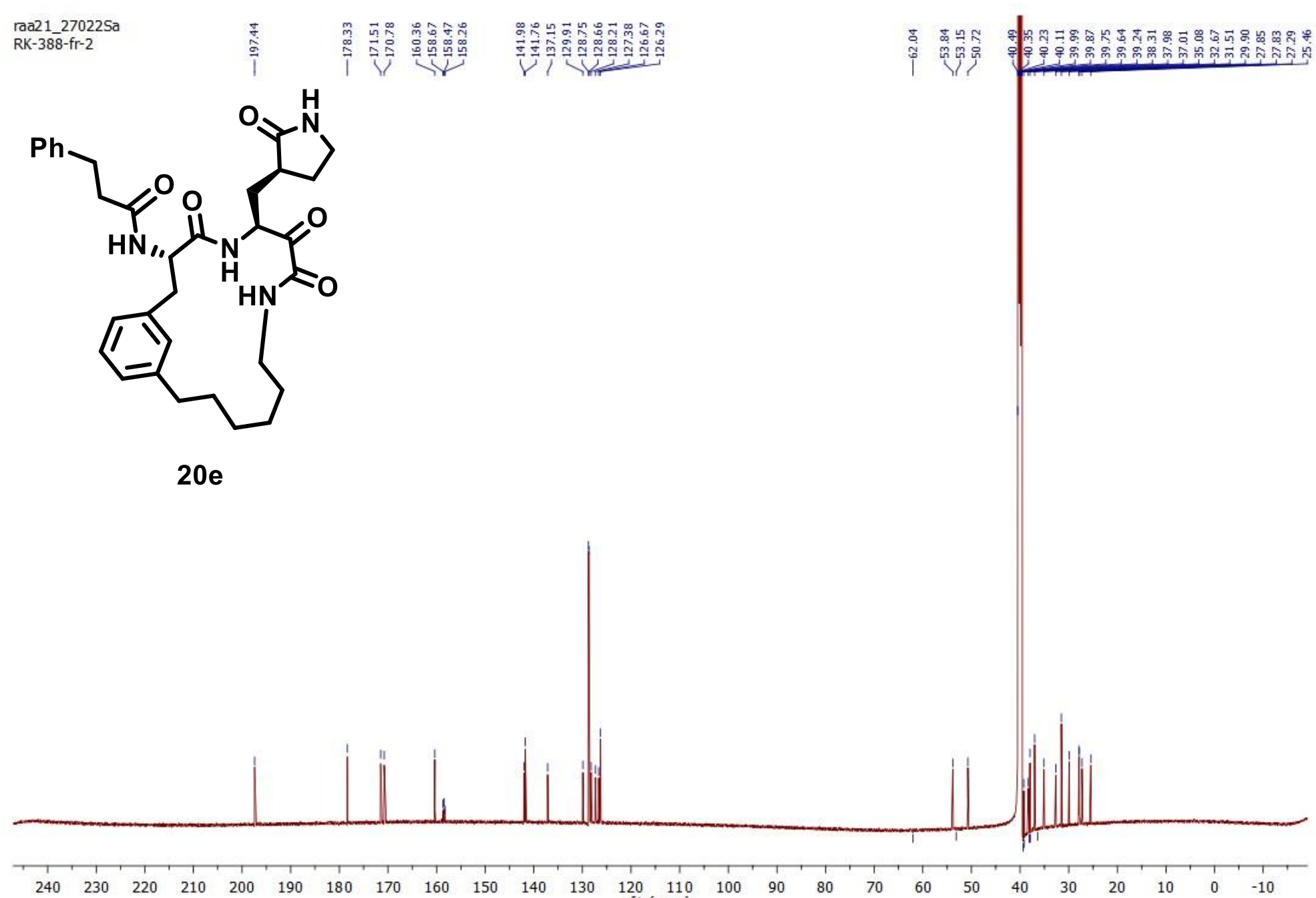

^{1}H-NMR (700 MHz, DMSO-d_6) of 20f.

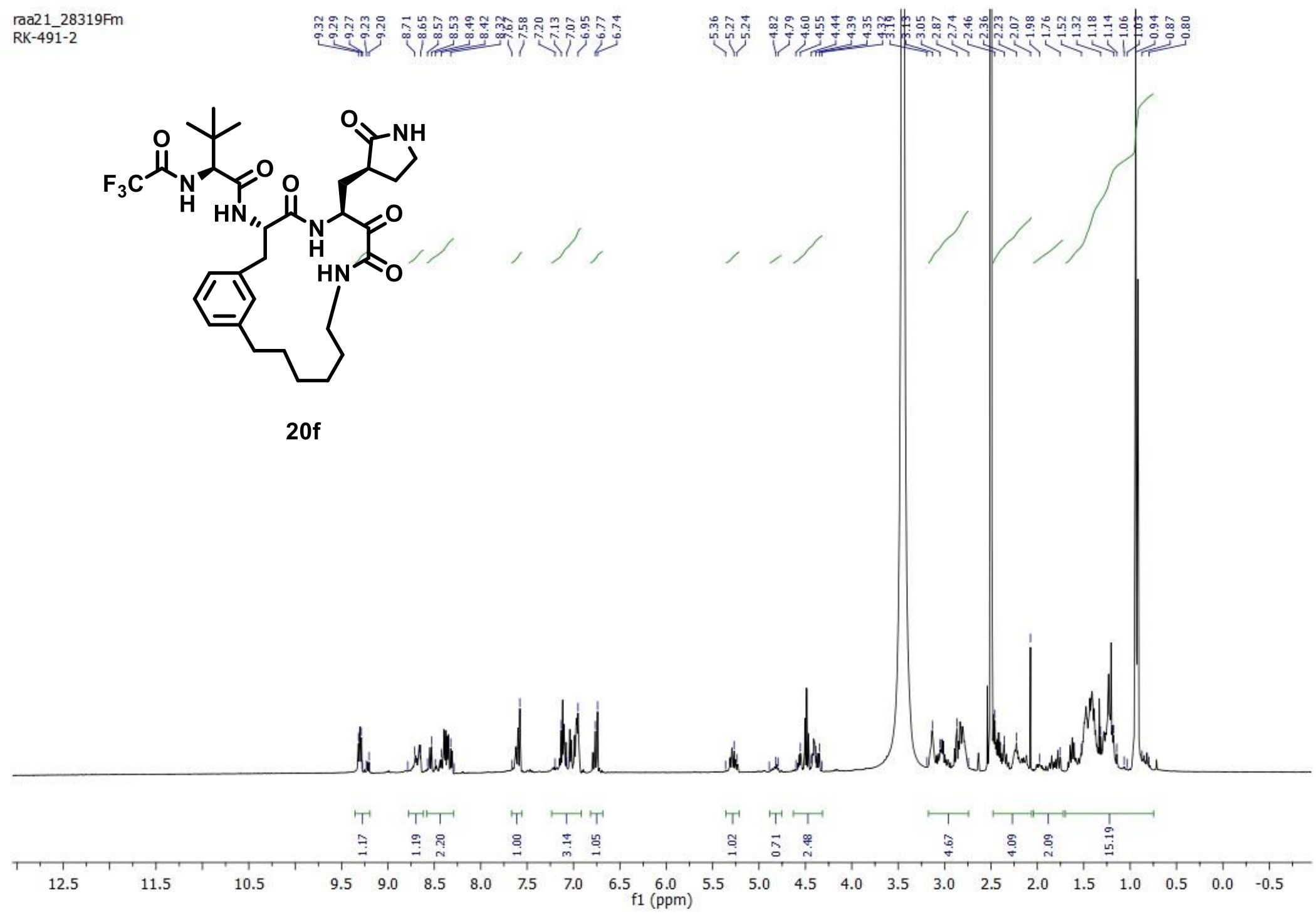

^{13}C-NMR (125 MHz, DMSO-d_6) of 20f.

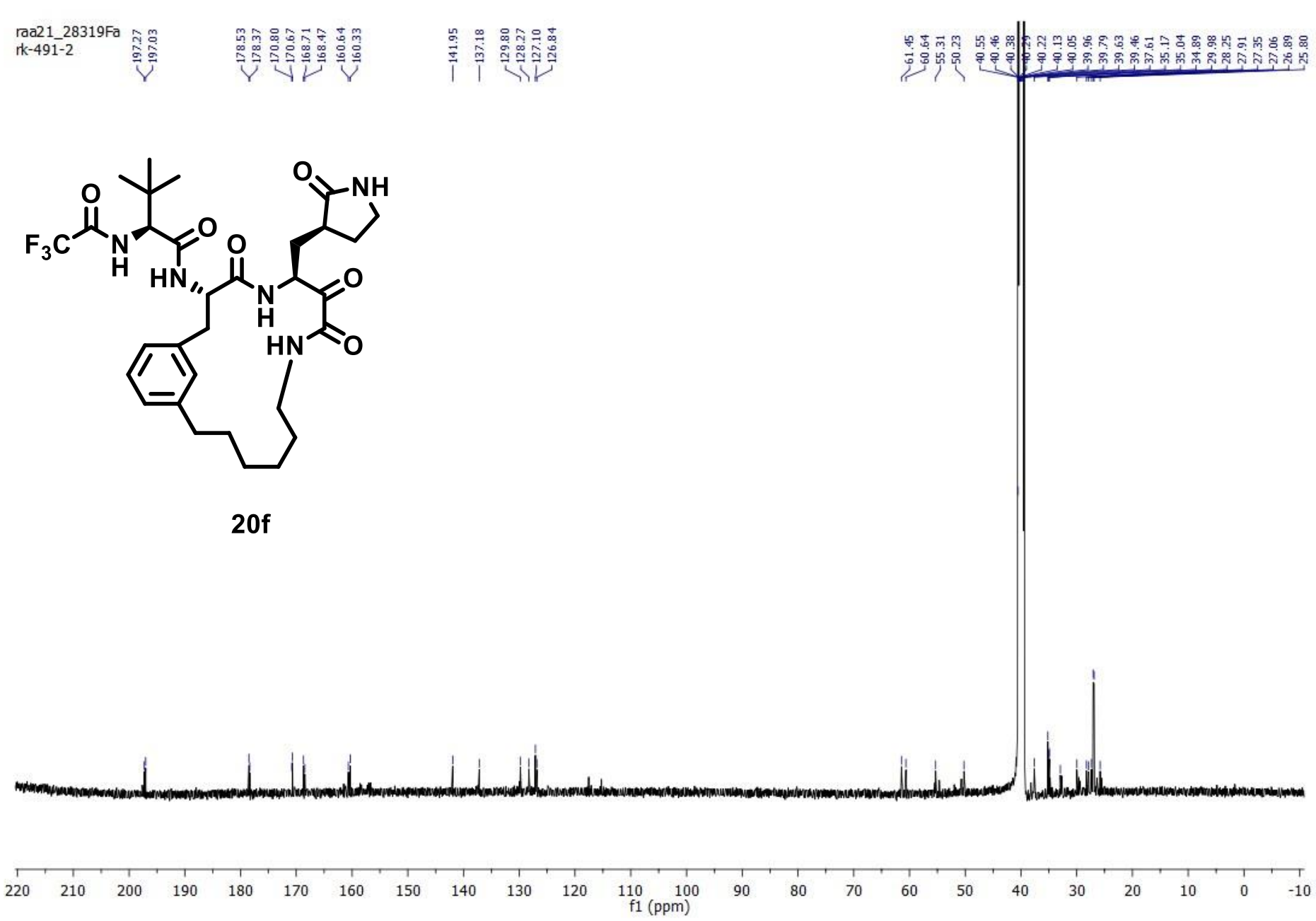

^{1}H-NMR (700 MHz, DMSO-d_6) of 20g.

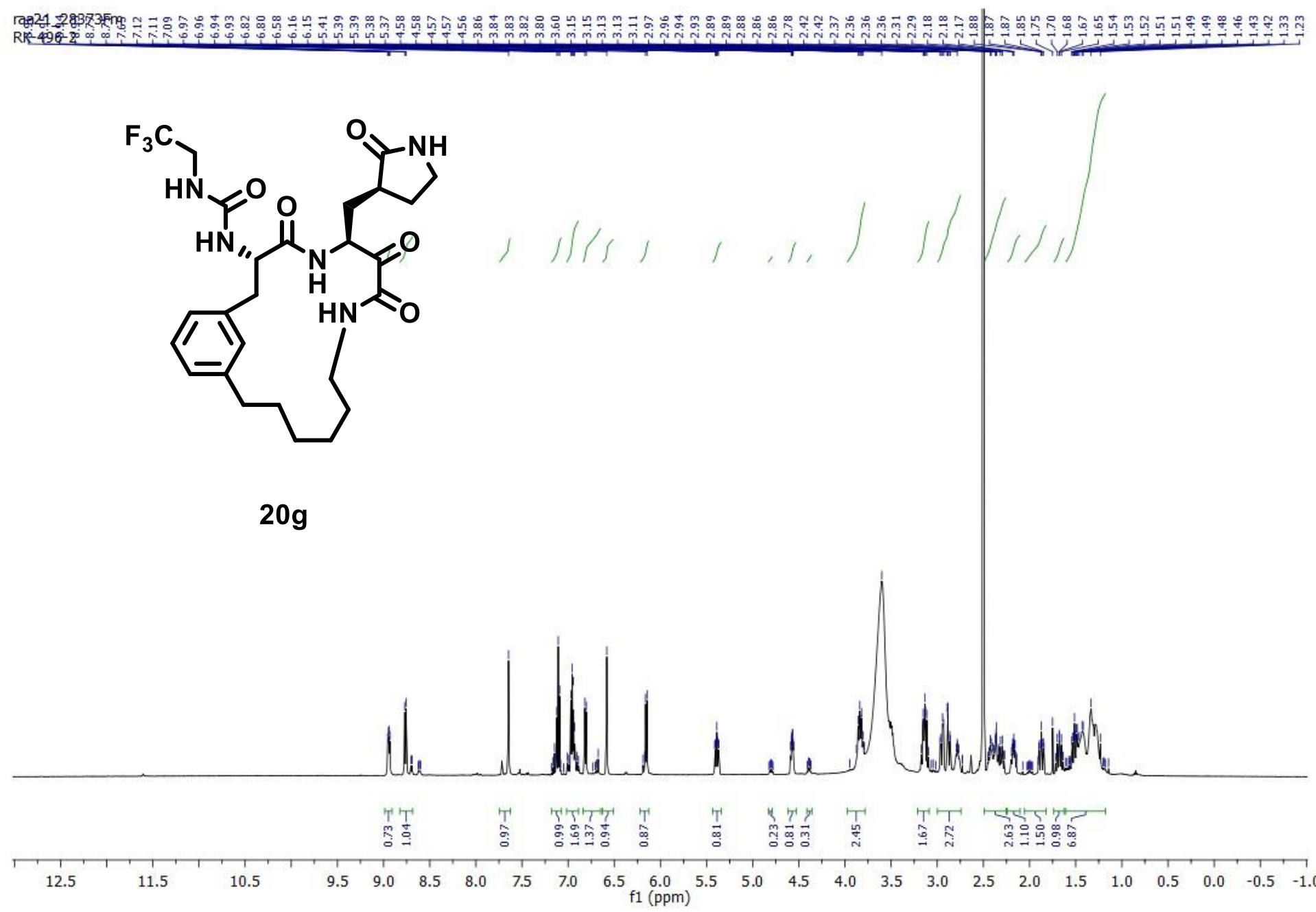

^{13}C-NMR (125 MHz, DMSO-d_6) of 20g.

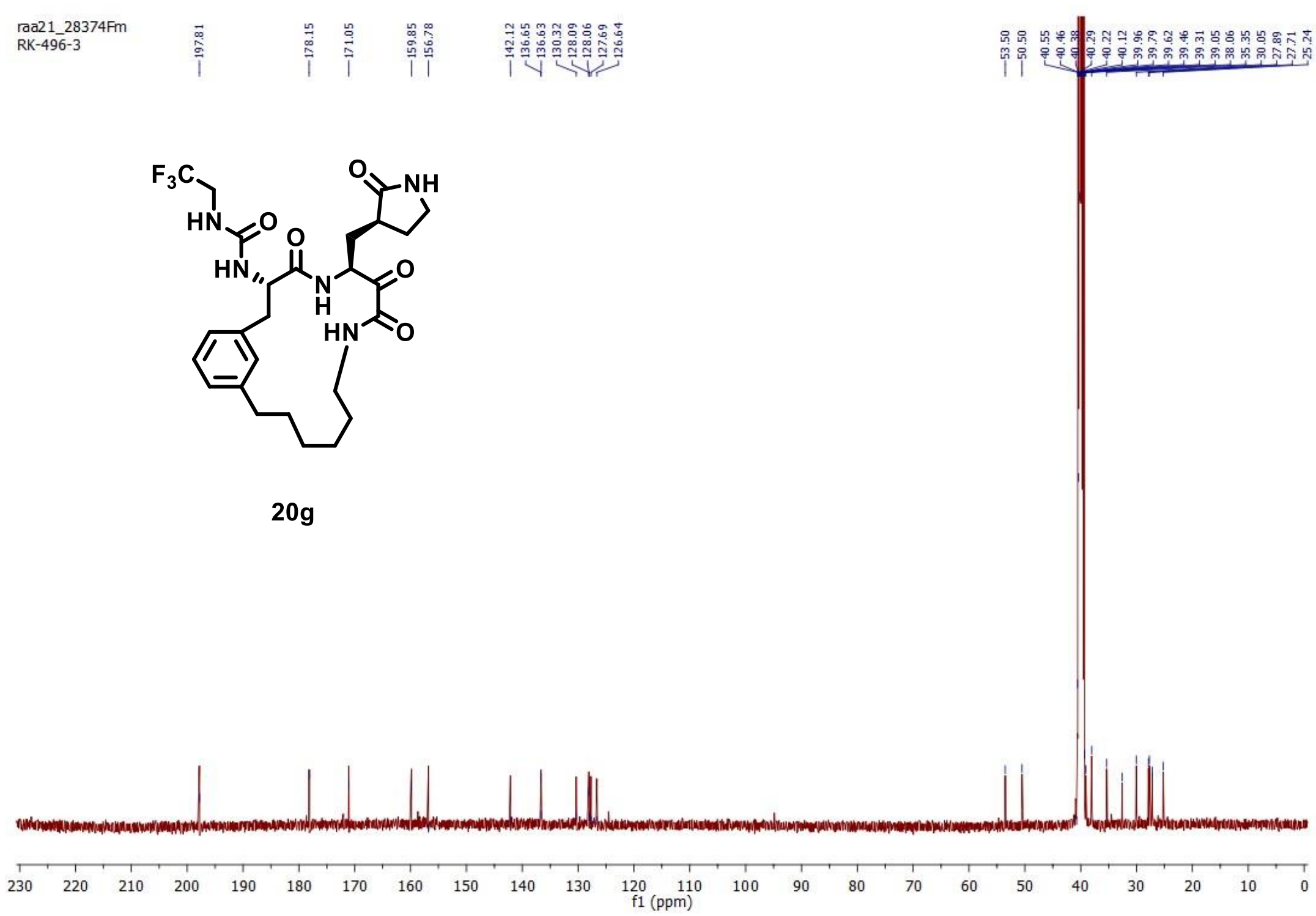

^{1}H-NMR (700 MHz, DMSO-*d*$_6$) of 20h.

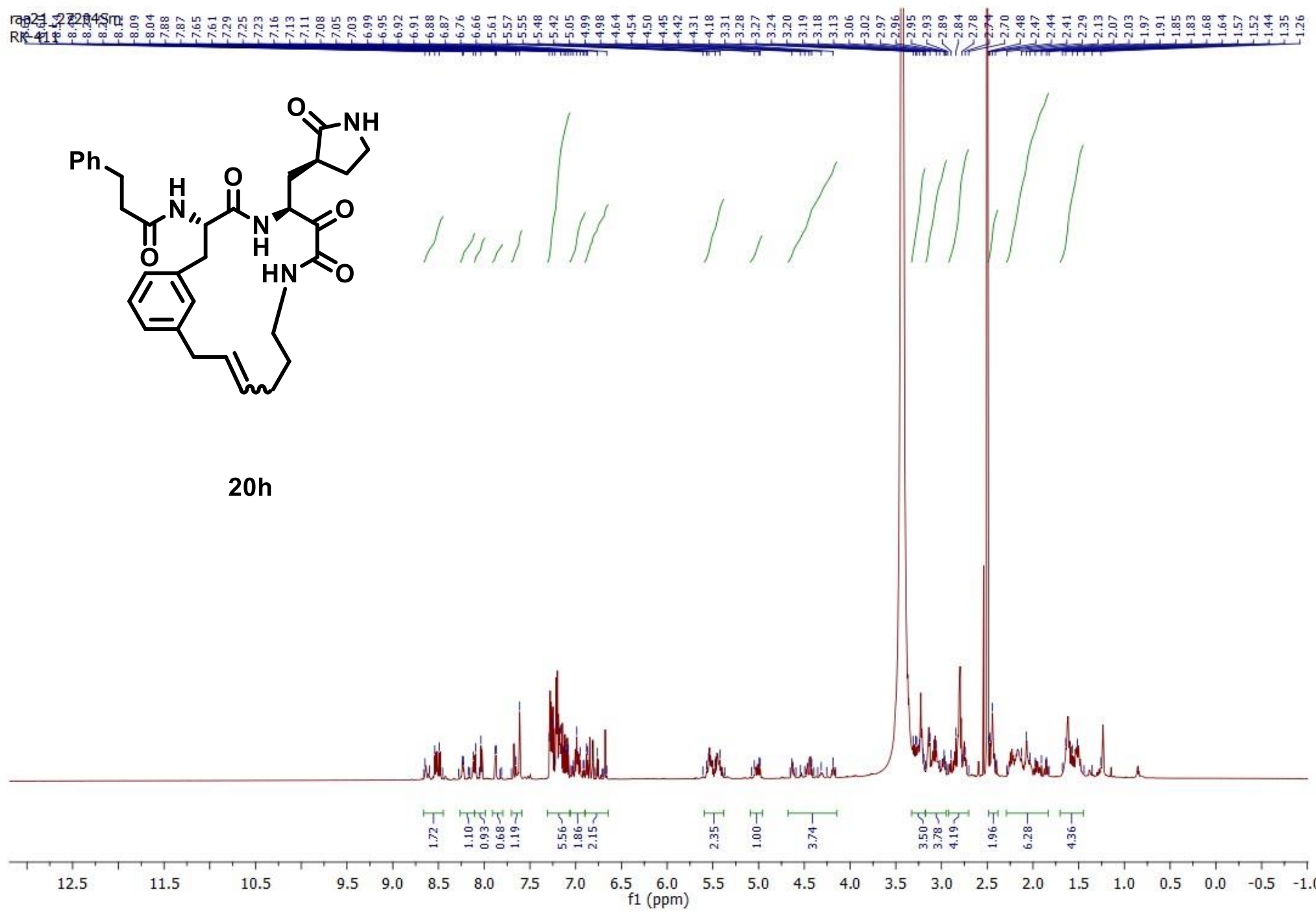

^{13}C-NMR (176 MHz, DMSO-*d*$_6$, DEPT-135, 298 K) of 20h.

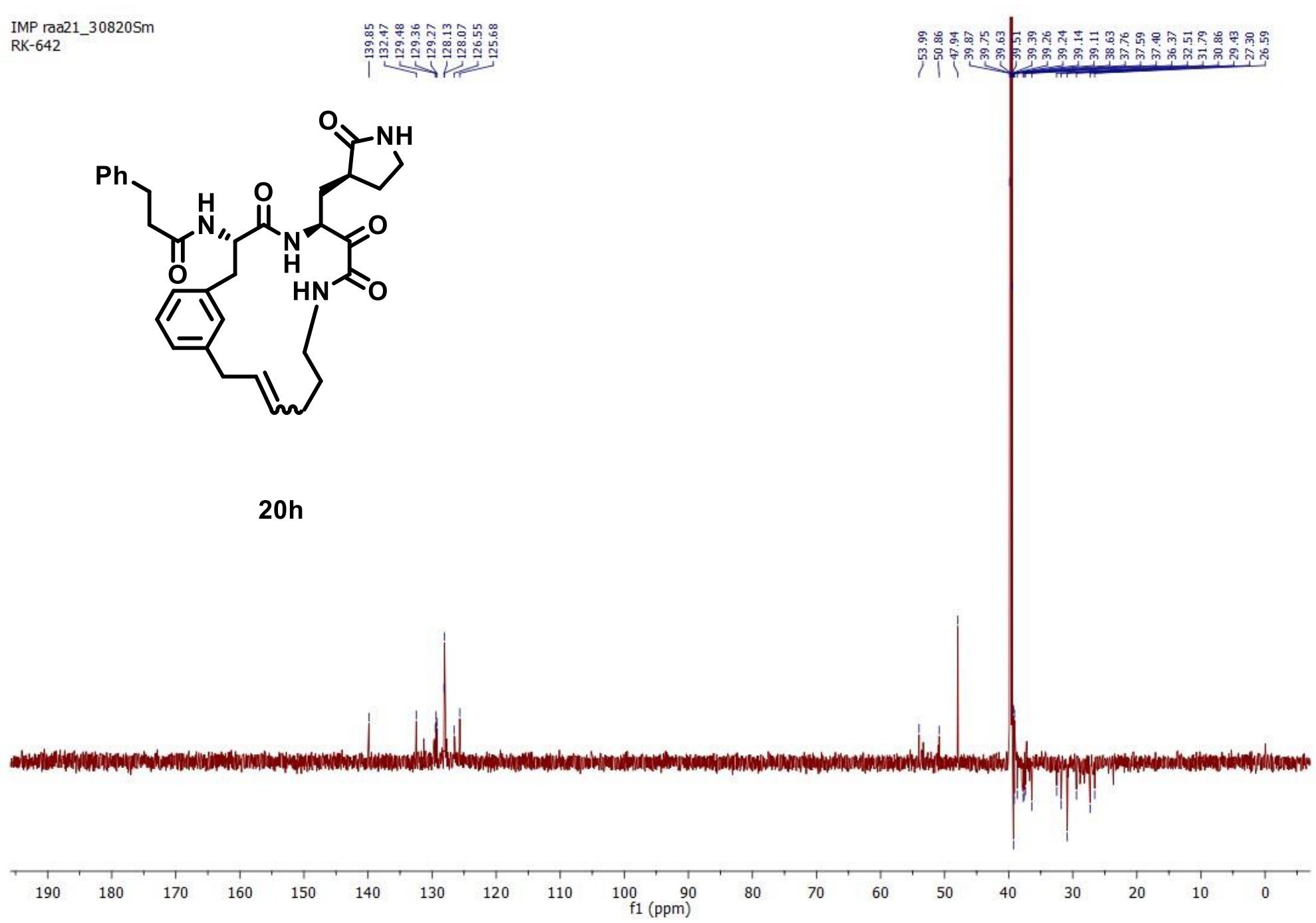

Figure S5. HPLC traces

Compound 9a-h

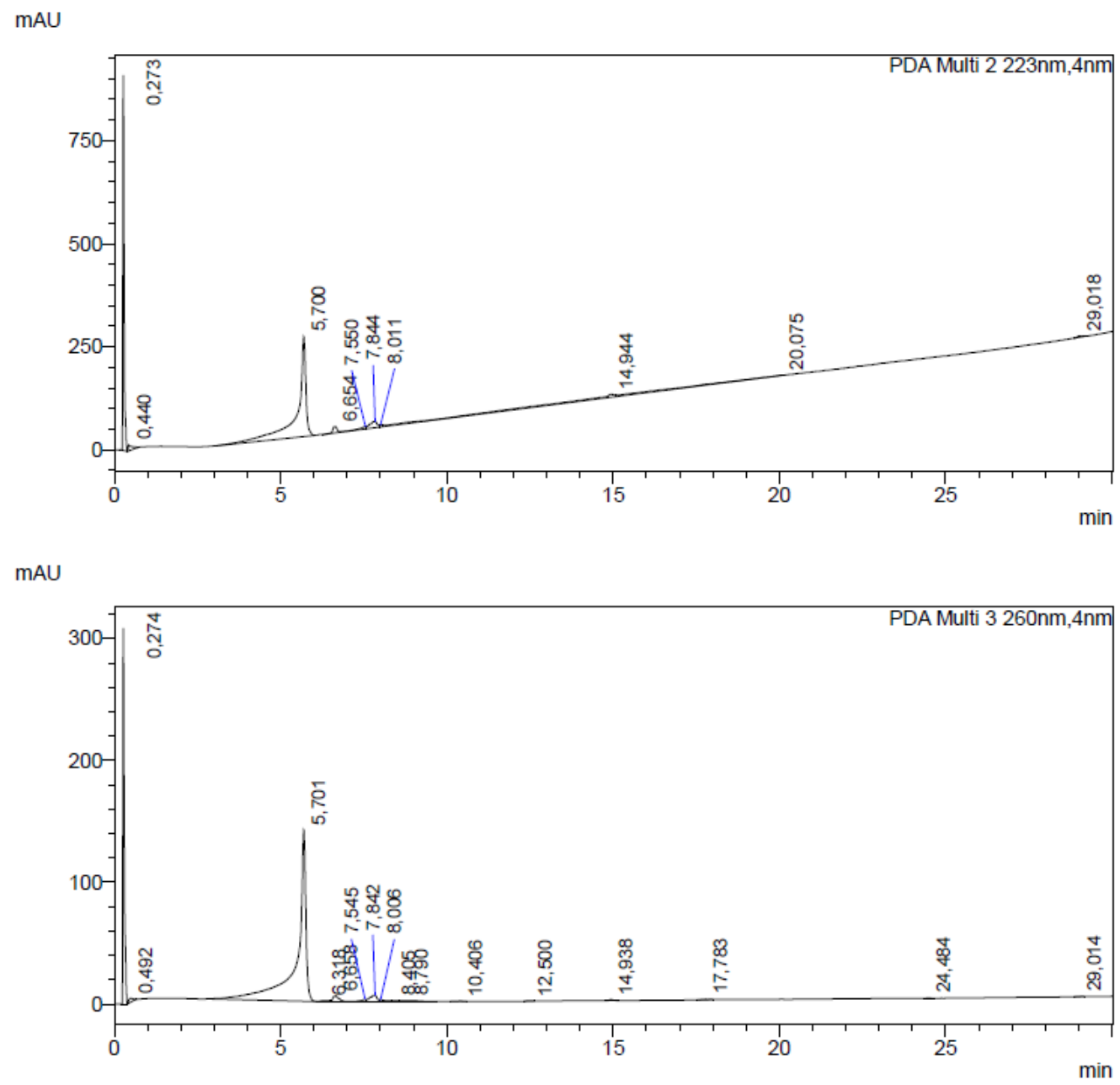

Compound 9a-K

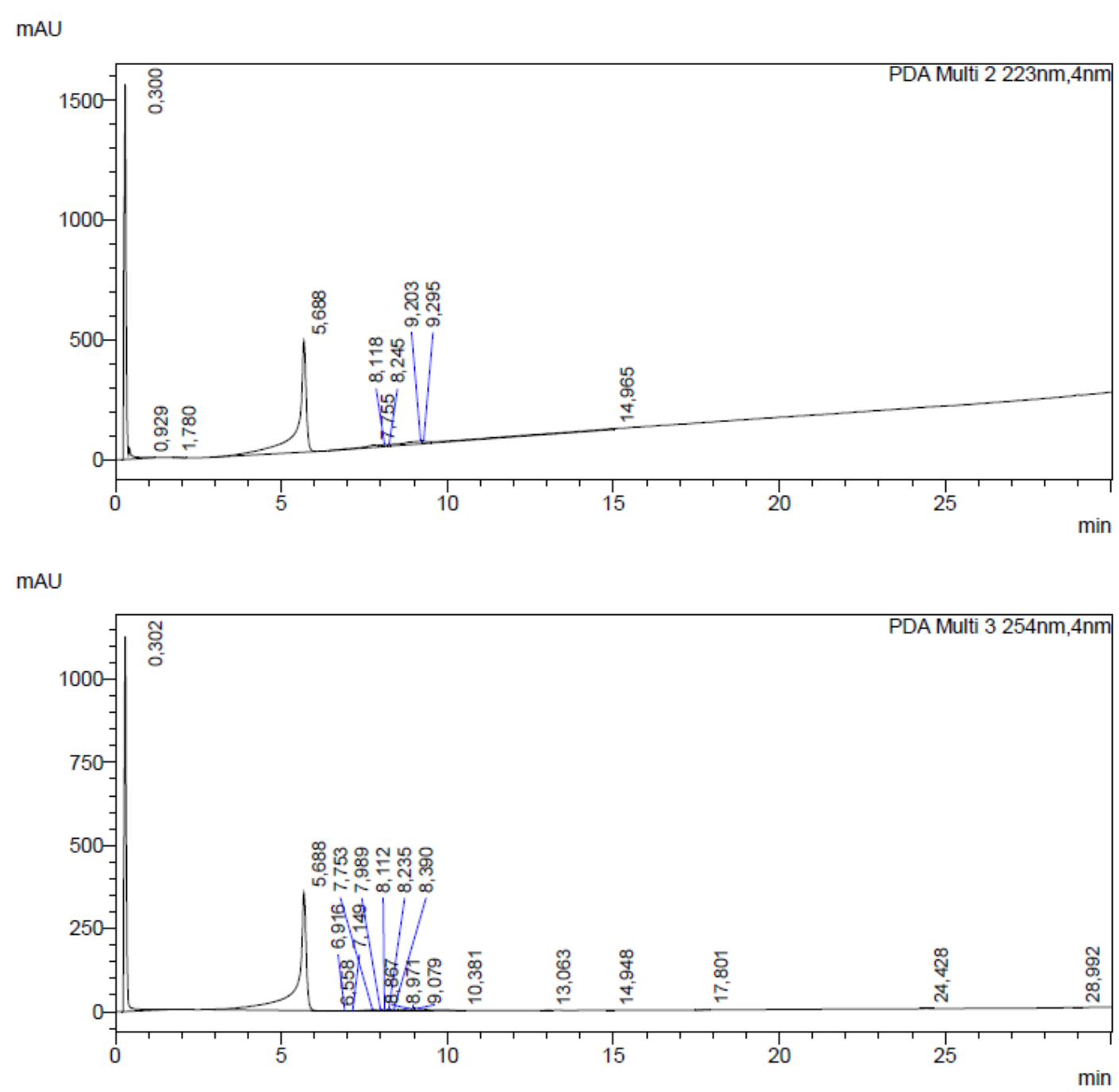

Compound 9b-K

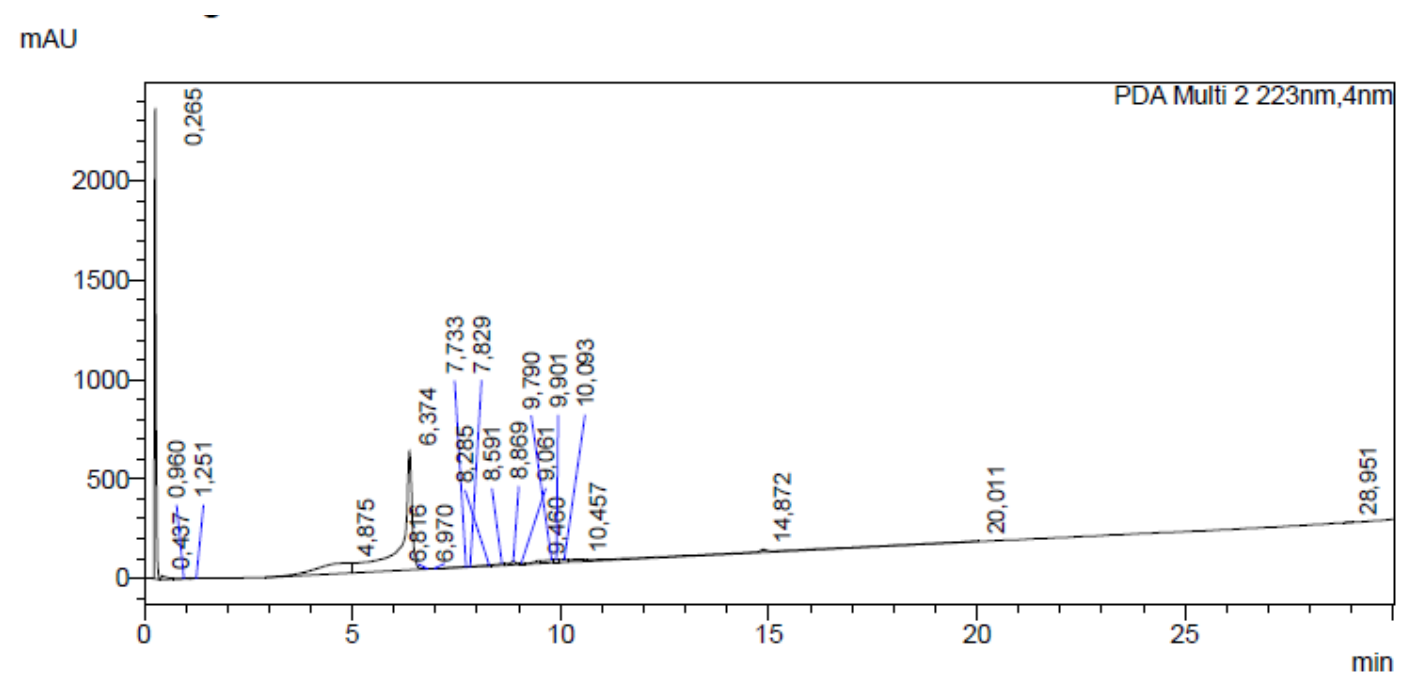

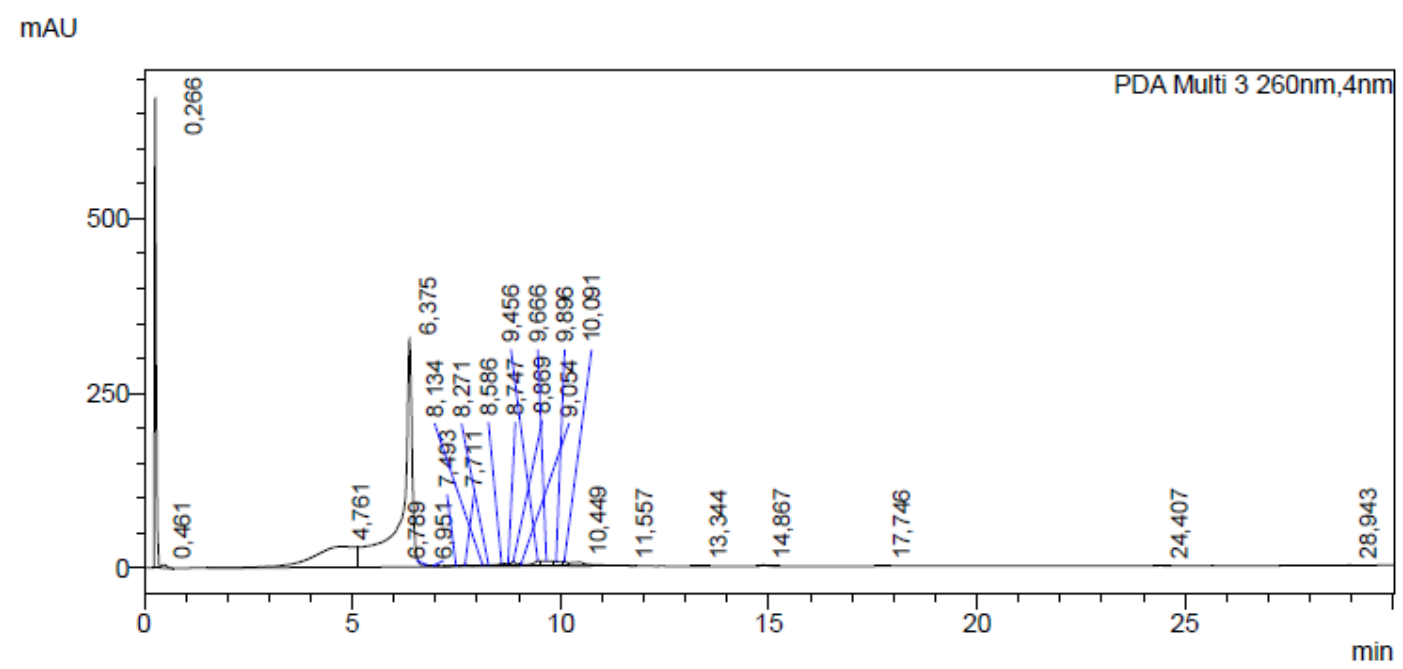

Compound 20a

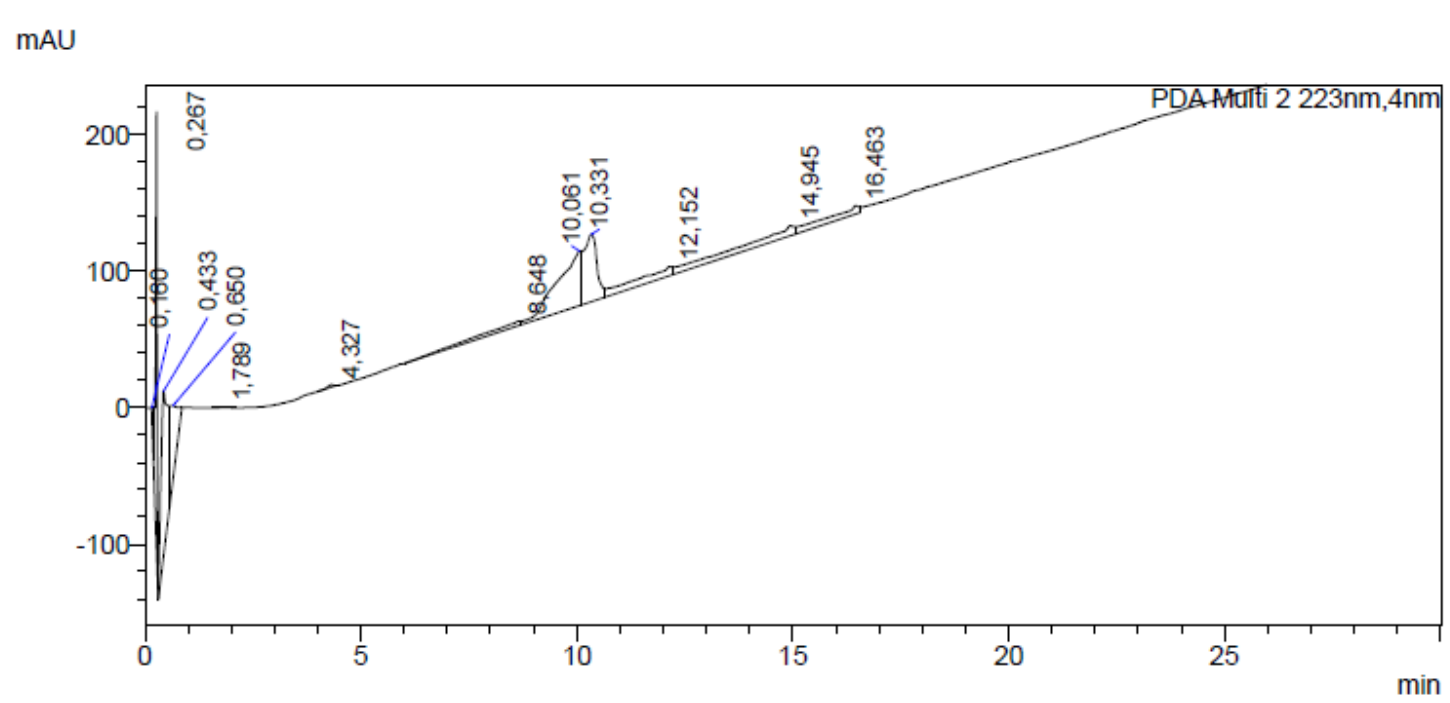

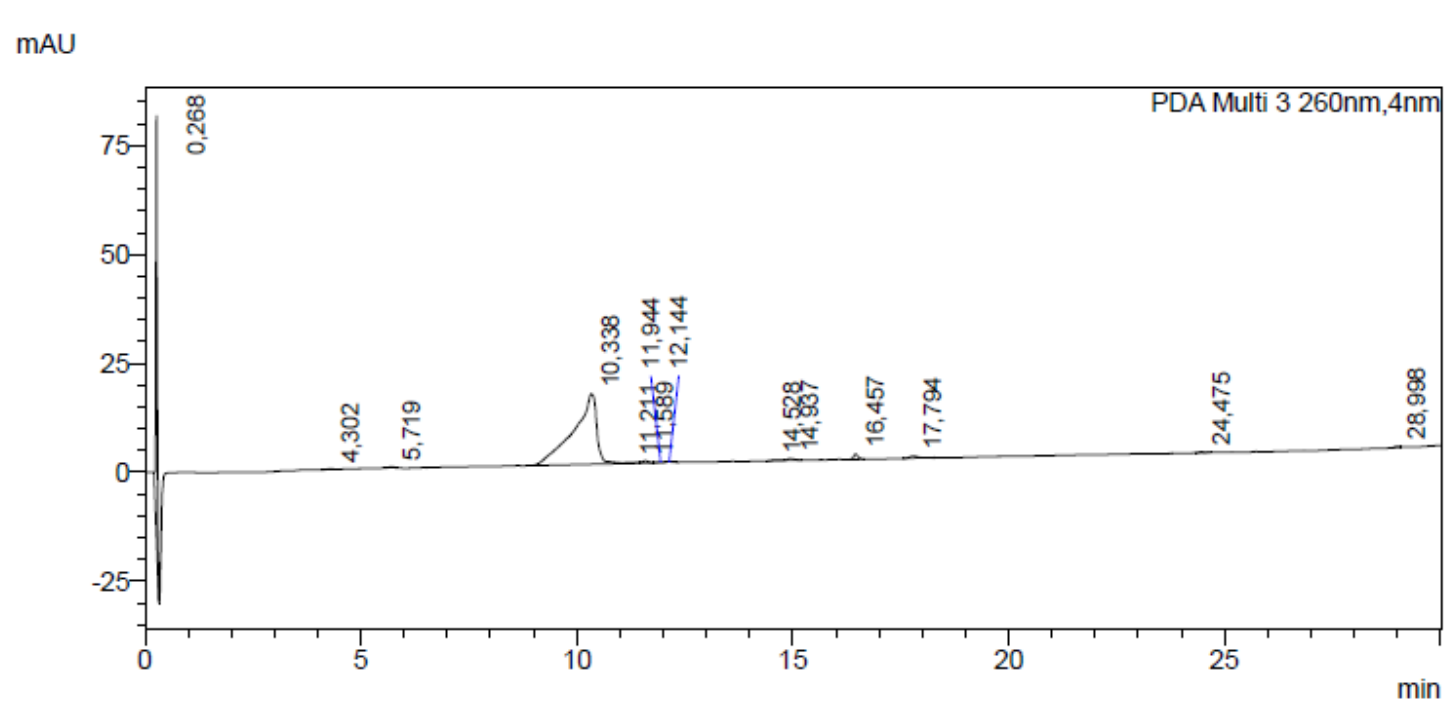

Compound 20b

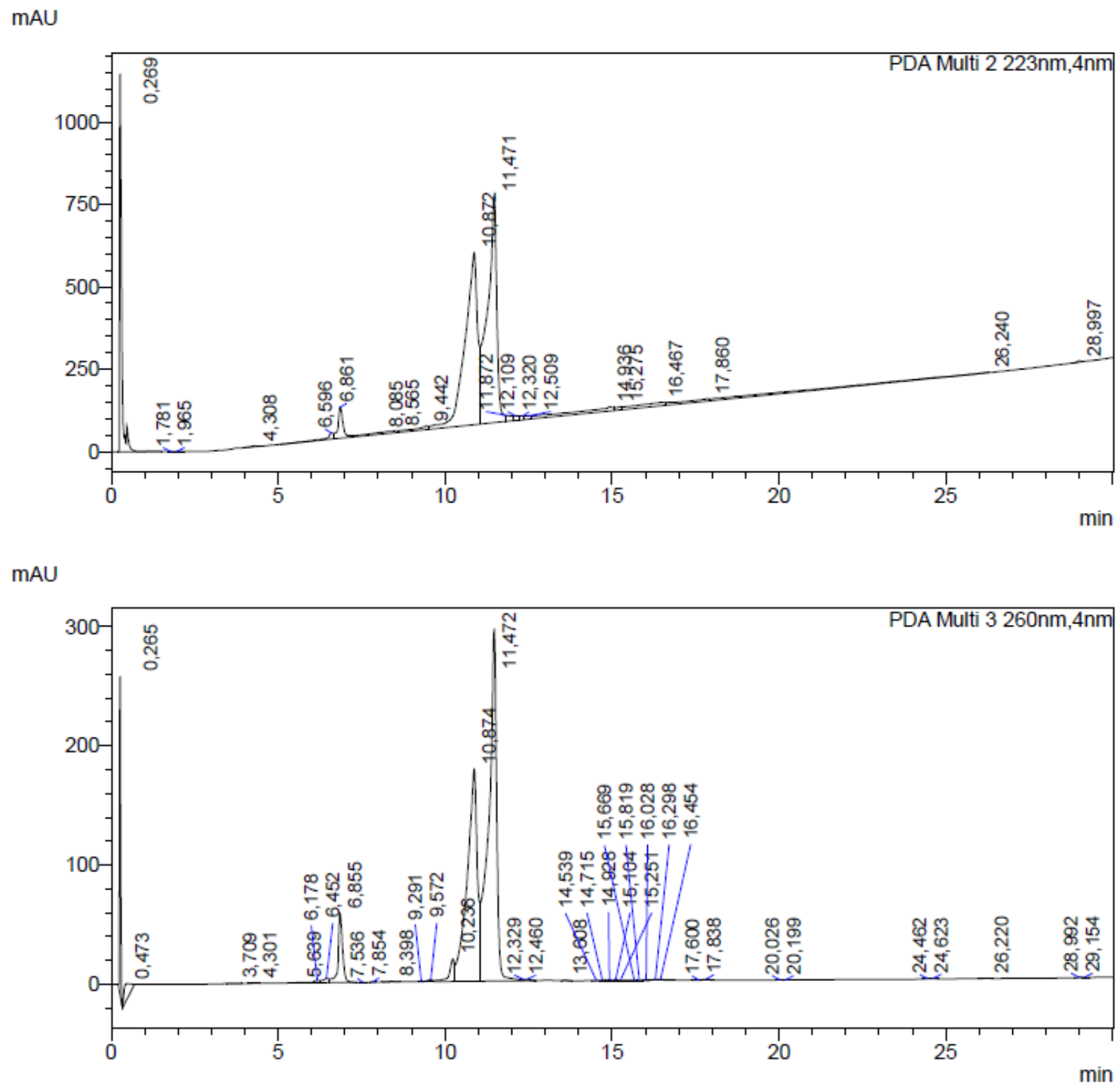

Compound 20c

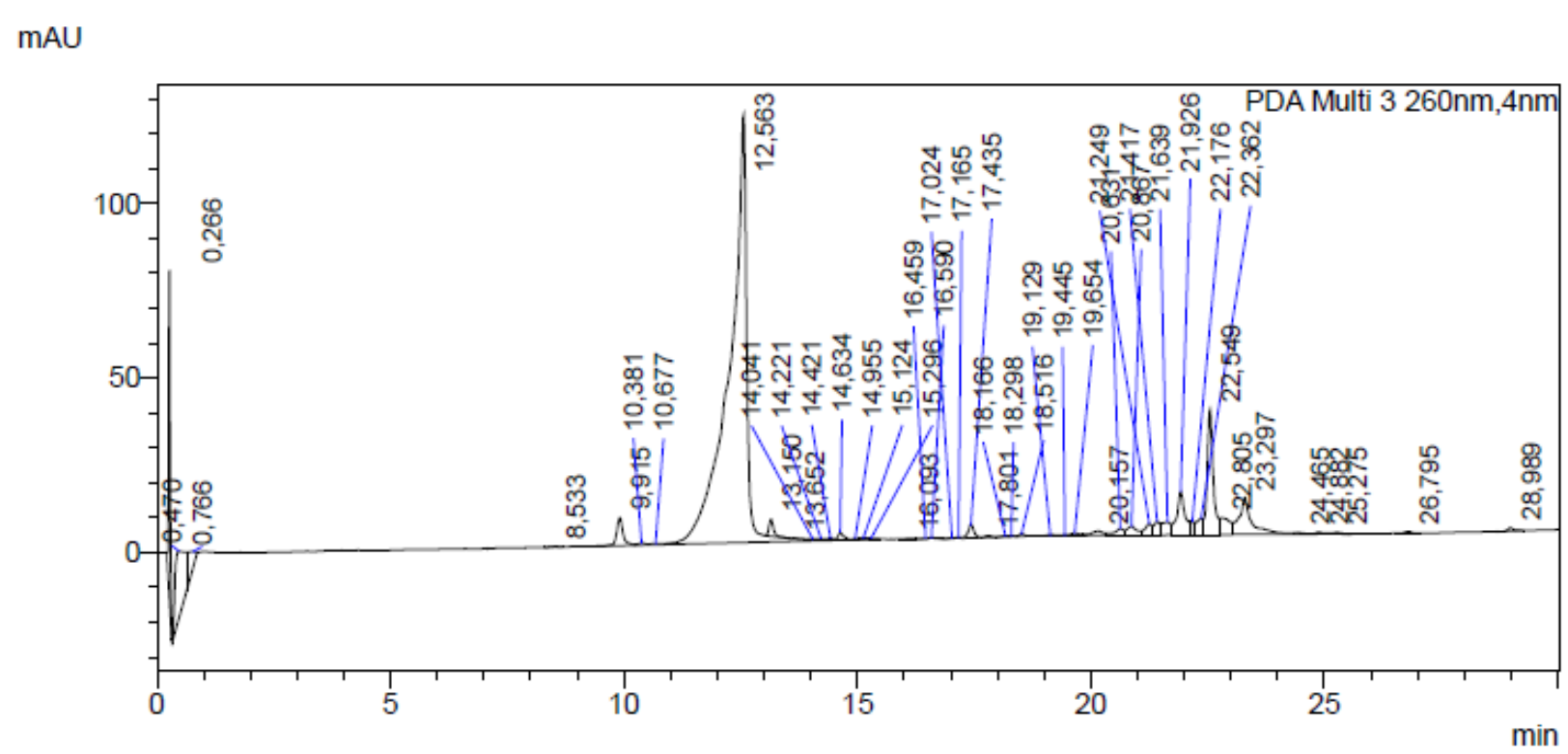

Compound 20d

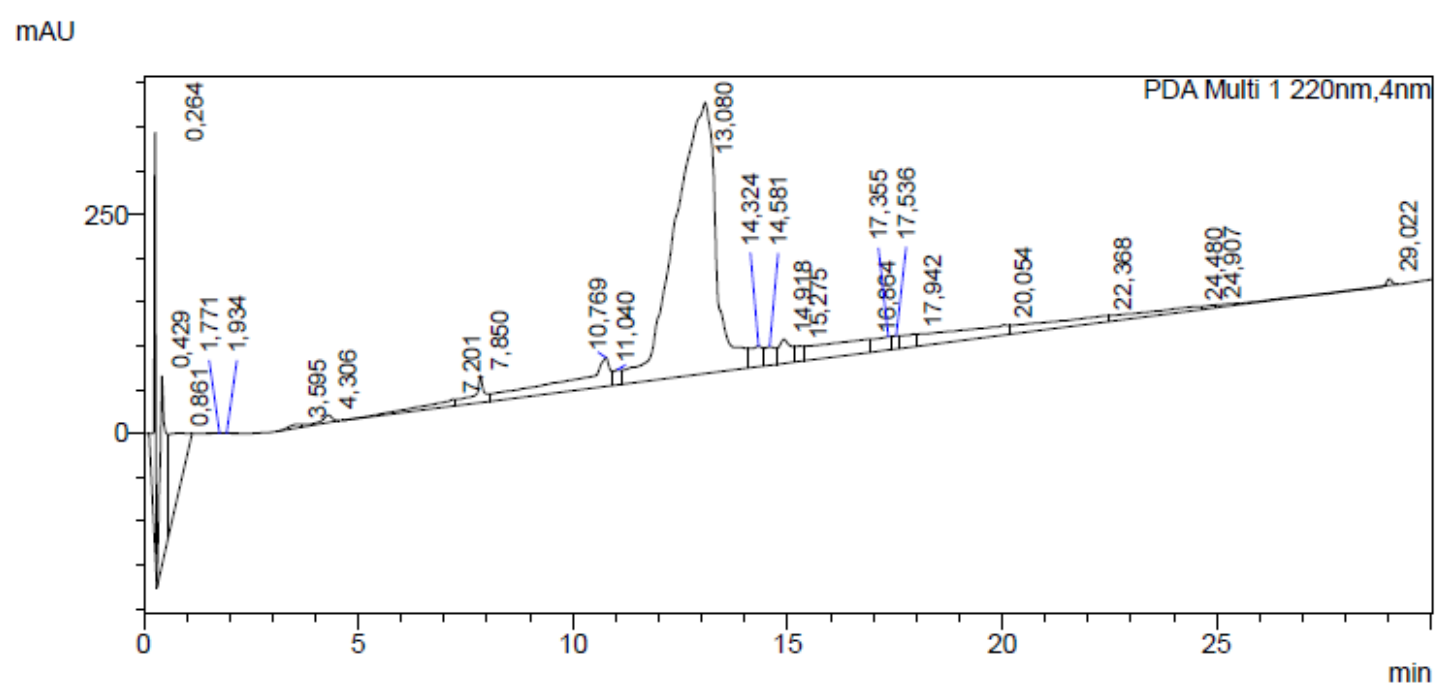

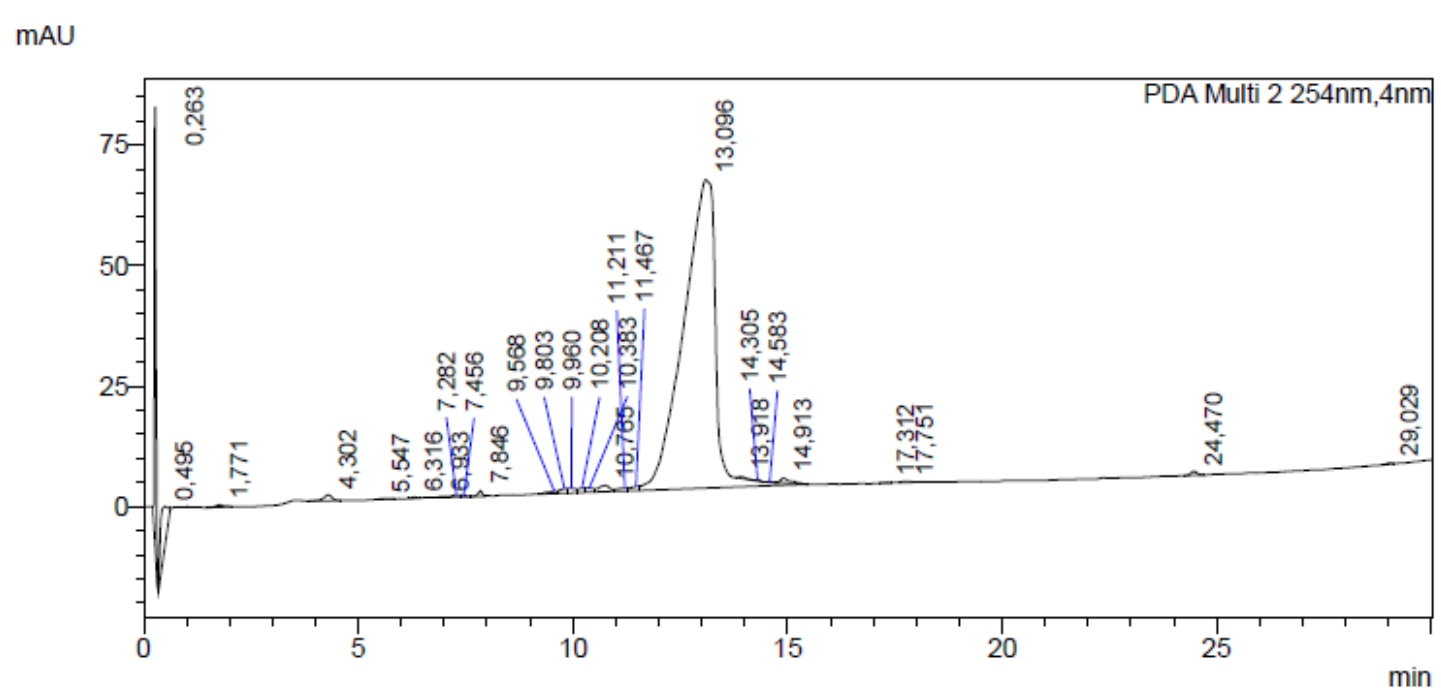

Compound 20e

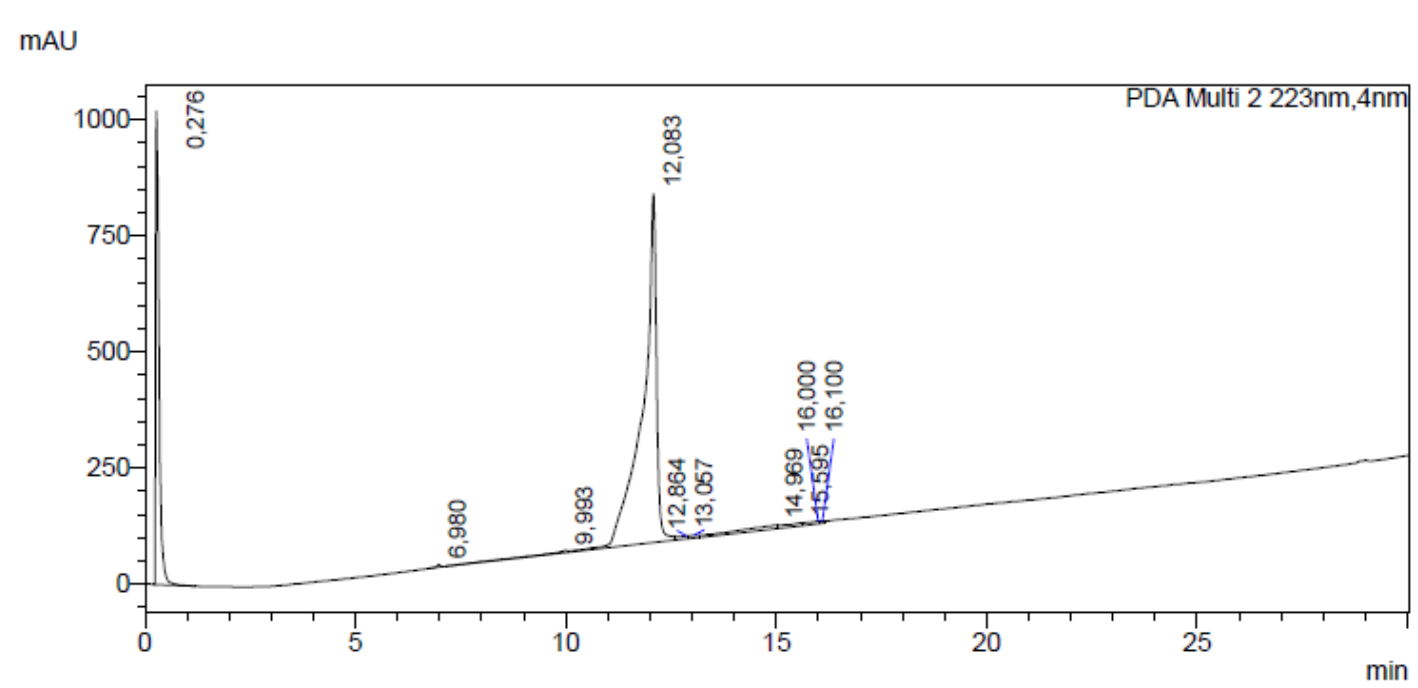

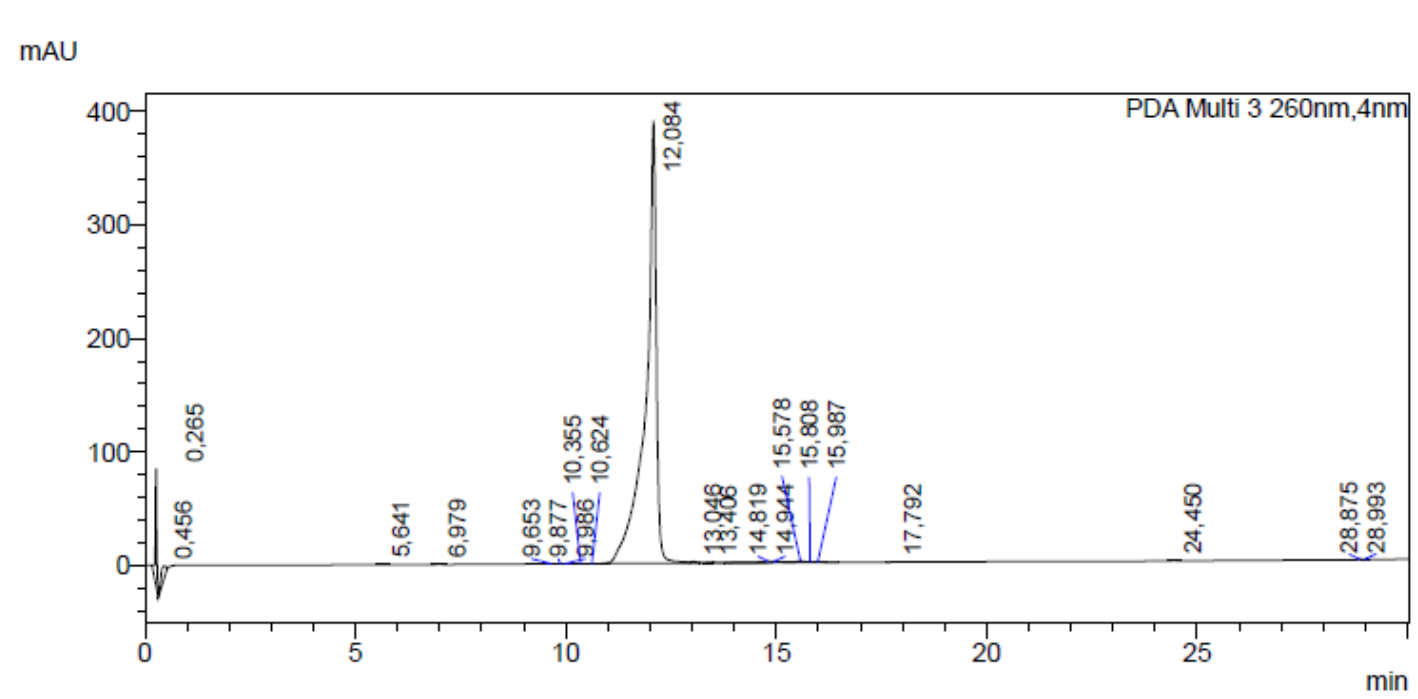

Compound 20f

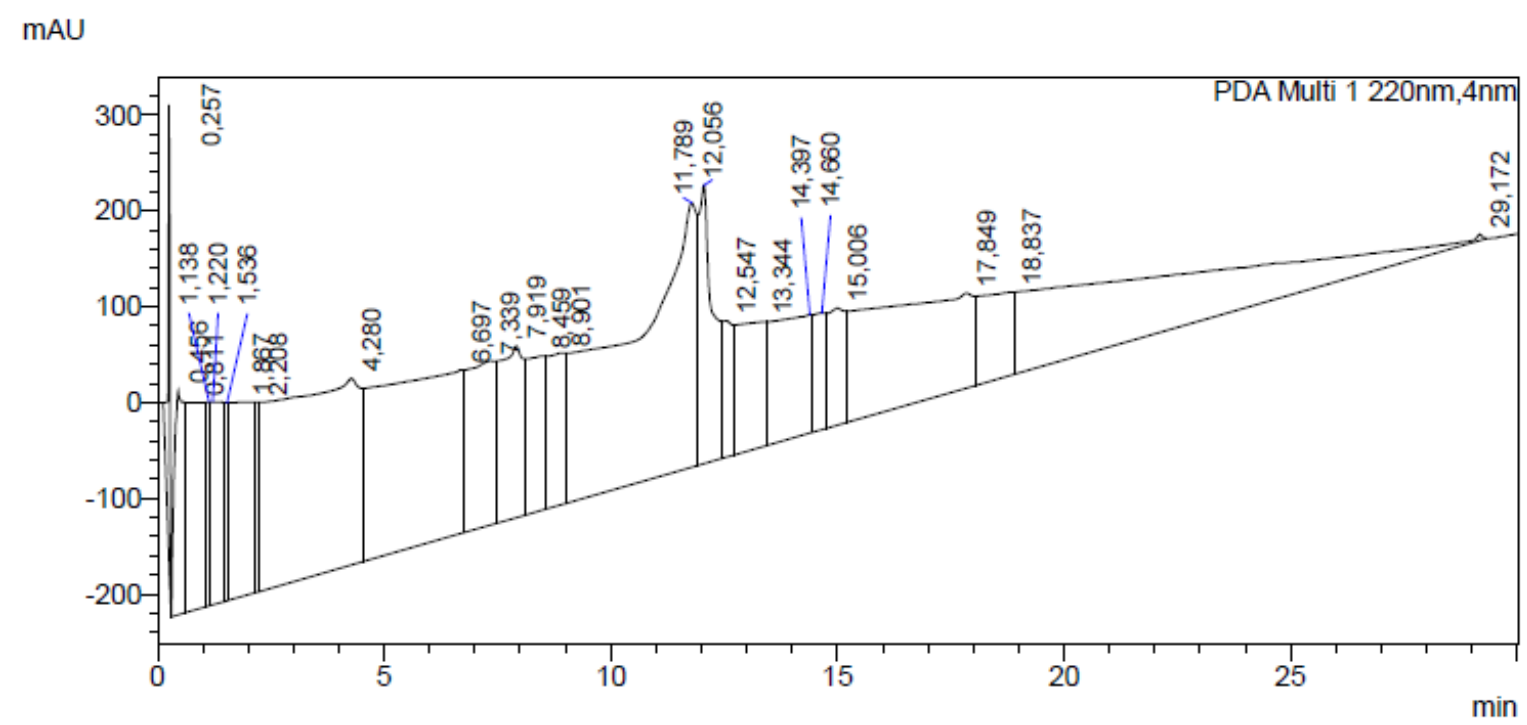

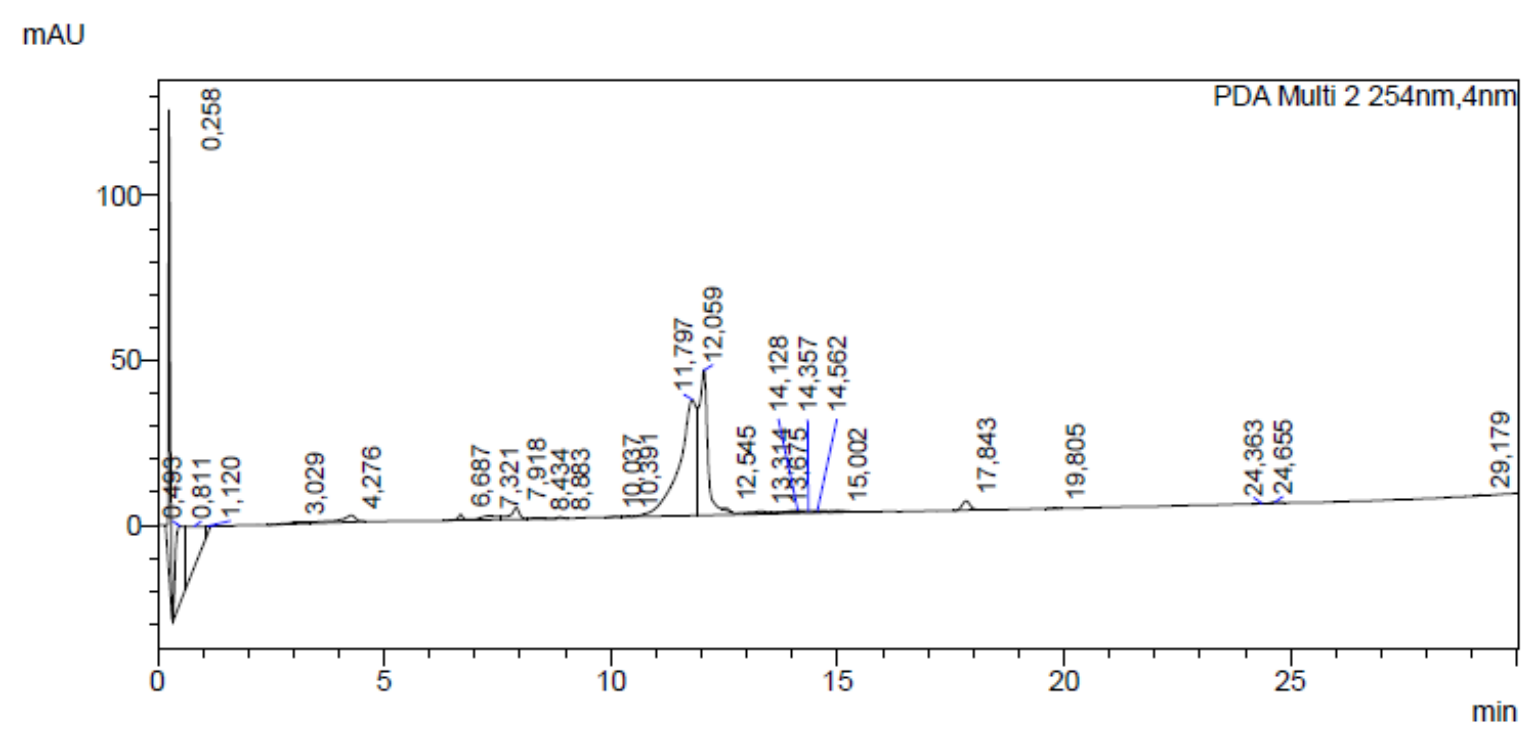

Compound 20g

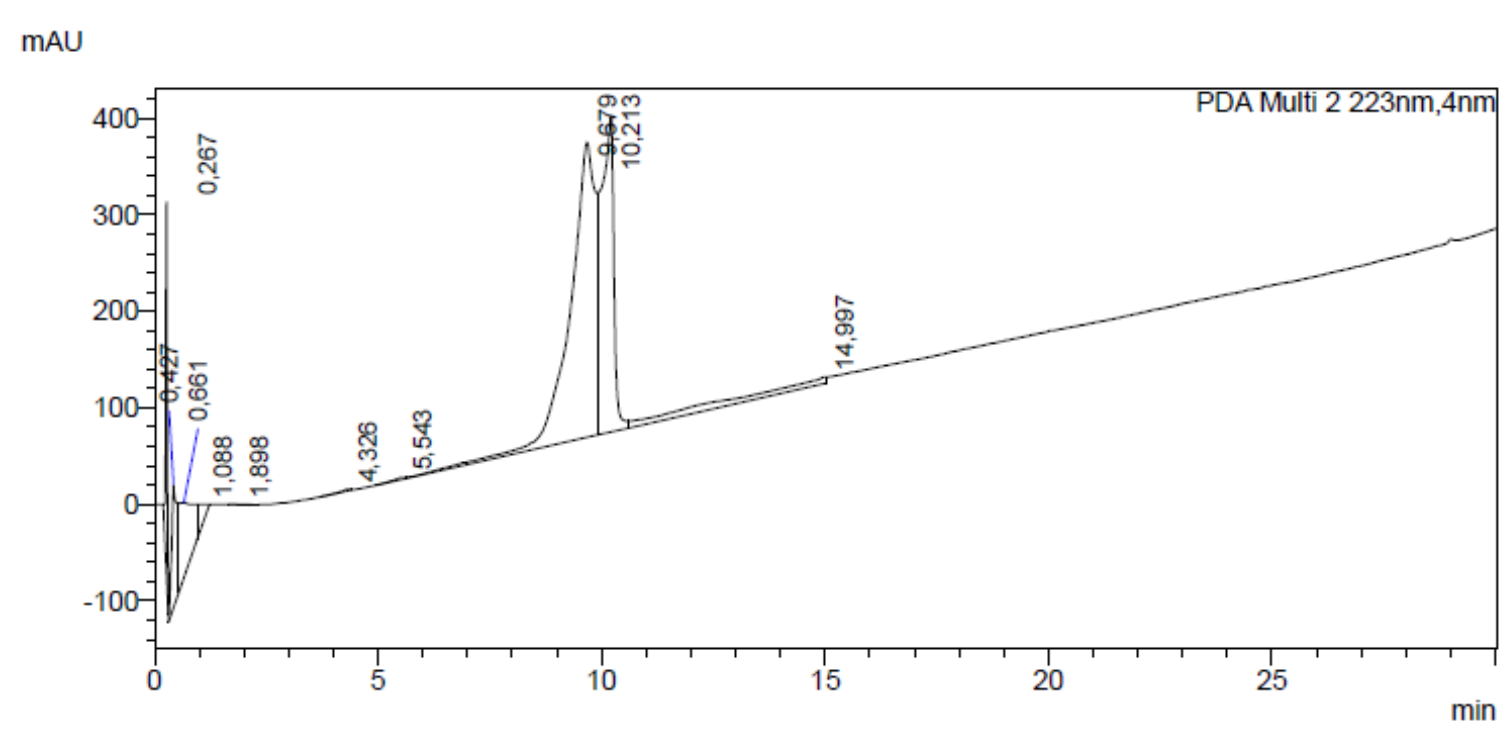

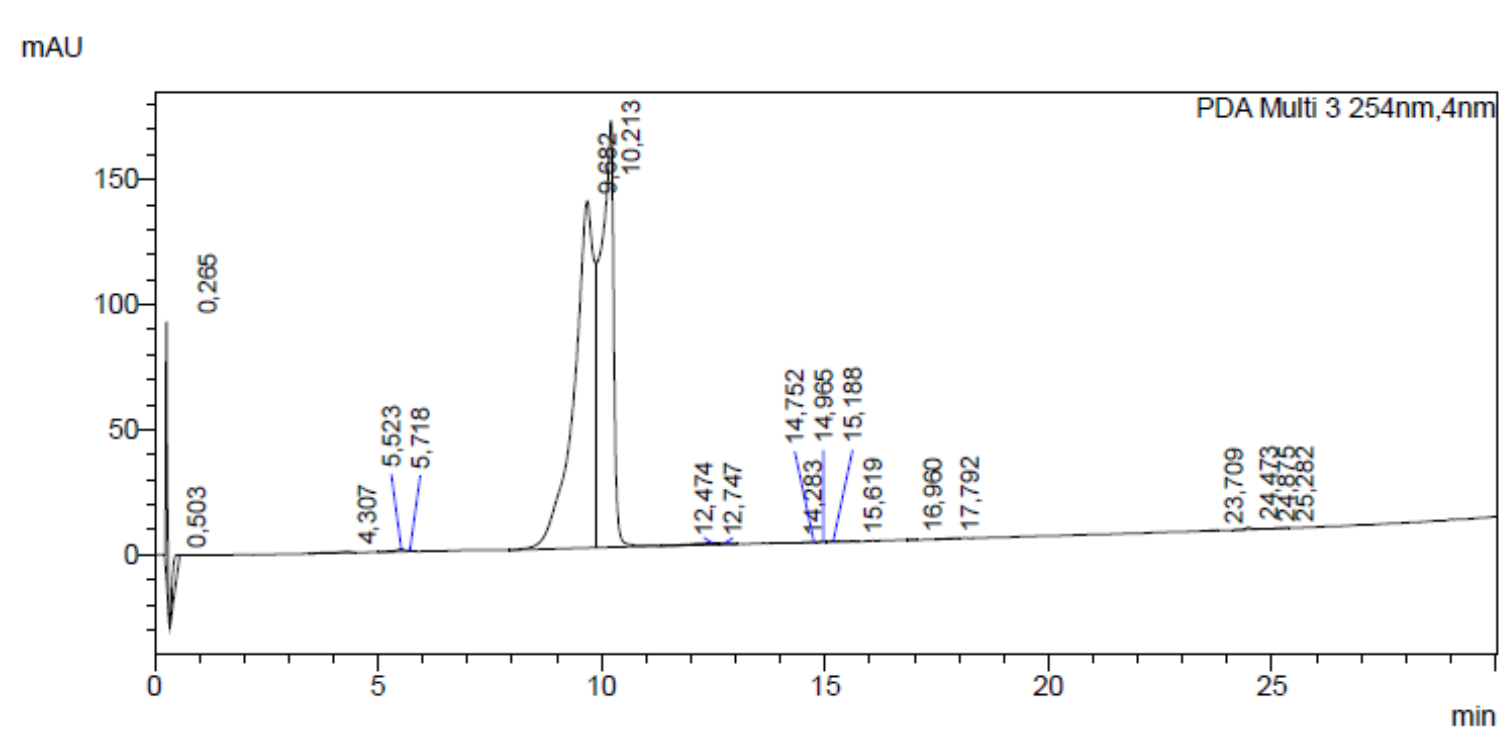

Compound 20h

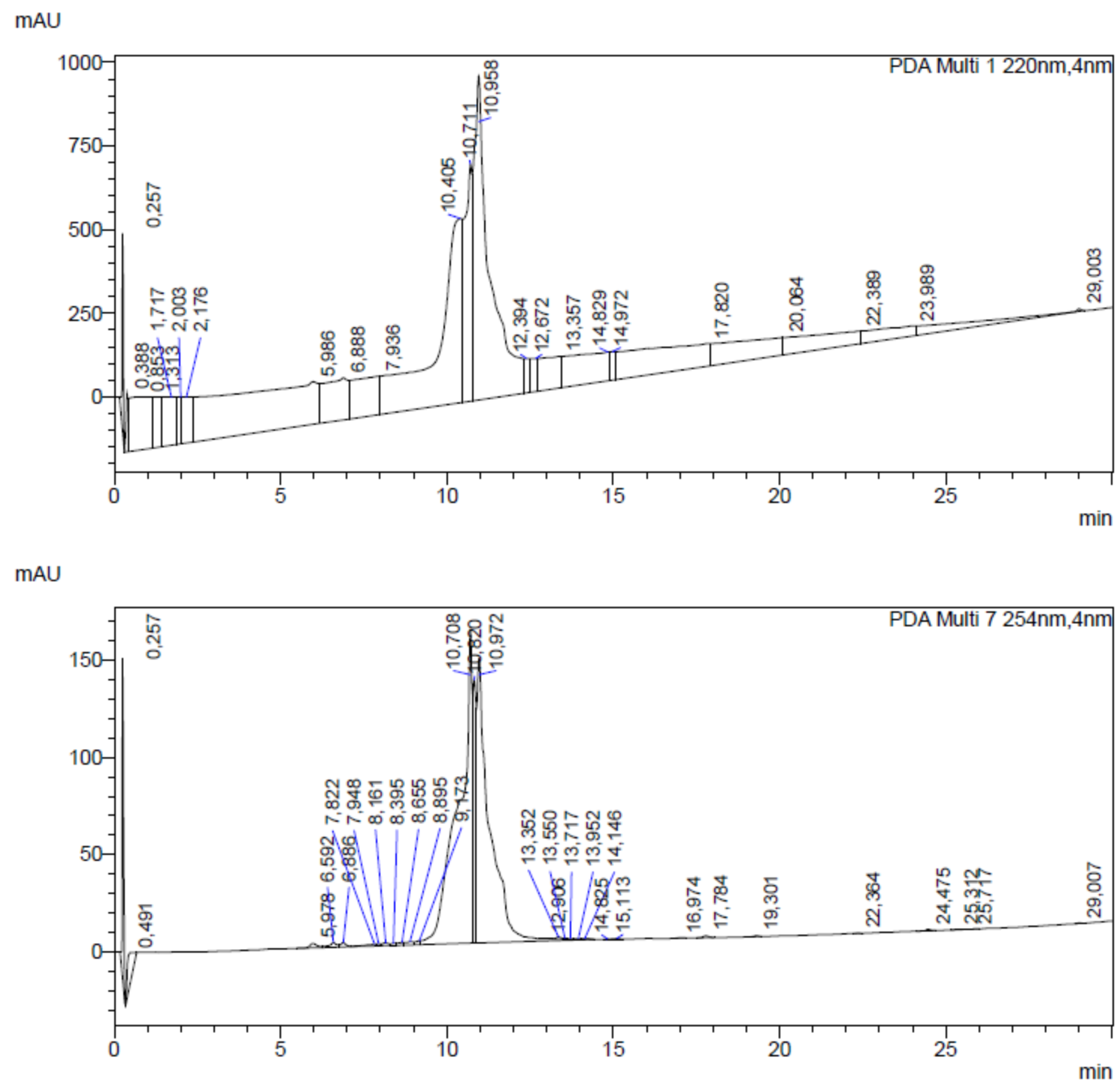

Supplementary References

1. Weiss, M.S. & Hilgenfeld, R. On the use of the merging R factor as a quality indicator for X-ray data. *J. Appl. Cryst.* **30**, 203–205 (1997).
2. Karplus, P.A. & Diederichs, K. Linking crystallographic model and data quality. *Science* **336**, 1030–1033 (2012).
3. Chen, V.B. et al. MolProbity: all-atom structure validation for macromolecular crystallography. *Acta Crystallogr. Sec. D.* **66**, 12–21 (2010).
